# Accurate Prediction of HOMO–LUMO Gap Using DFT Functional and Application to Next‐Generation Organic Telluro[n]Helicenes Materials

**DOI:** 10.1002/jcc.70175

**Published:** 2025-07-02

**Authors:** Rahul Kumar, Rahul Kar, Dilip K. Maity

**Affiliations:** ^1^ Homi Bhabha National Institute, Training School Complex Mumbai India; ^2^ Bhabha Atomic Research Centre Mumbai India; ^3^ Department of Chemistry Dibrugarh University Dibrugarh India

**Keywords:** accurate HOMO–LUMO energies, dimerization study, helicenes, next‐generation organic materials, statistical error analysis, tellurophene, UV–visible spectra

## Abstract

The present work purposes and establishes an accurate prediction of HOMO–LUMO energies of thiophene‐, selenophene‐, and tellurophene‐based helicenes using 15 different DFT methodologies. DFT functionals used in this work are PBE, PBE0, B3LYP, B3LYP‐D, B3LYP‐D3, M06, MN15, HSE06, LC‐BLYP, CAM‐B3LYP, LC‐ωPBE, ωΒ97XD and B2PLYP. DFT HOMO–LUMO gaps are compared with the fundamental gaps calculated at the CCSD(T) level of theory. The LANL2DZ basis set is used for tellurium atoms, and the 6–311++G(d,p) basis set is used for other elements. Statistical error analysis suggests that the HOMO–LUMO energy gaps can be accurately obtained using ωB97XD functional, with geometry optimization performed at the same theoretical level. However, geometry optimization using the B3LYP functional, followed by single‐point energy calculation with the ωB97XD functional, provides a more cost‐effective method with similar accuracy for energy gap prediction. HOMO–LUMO gaps of telluro[n]helicenes ([n]TeH) are redshifted compared with their S‐ and Se‐analogs. Tellurophene‐based helicenes ([n]TeH) systems are easy to oxidize in contrast to their S‐ and Se‐analogs. Dimerization studies have found that substituted [7]TeH^•+^ is more stable in dichloromethane than its S‐ and Se‐analogs. The CAM‐B3LYP and ωΒ97XD functionals are used in conjunction with the TDDFT procedure to explore the excited states of [n]TeH radical cations. These radical cation systems showed better absorption in the infrared range than S‐ and Se‐systems. Overall, our benchmarking studies lead to an accurate prediction of HOMO–LUMO gaps of [*n*]TeH. Further, this study demonstrates the potential of Te‐based helical structures to create versatile next‐generation organic materials.

## Graphical abstract

Precise prediction of HOMO–LUMO energy gaps for thiophene‐, selenophene‐, and tellurophene‐based helicenes utilizing 15DFT methodologies in comparison to the CCSD(T) method is presented. The ωB97XD functional has been determined to be the best for gap prediction. Telluro[n]helicenes ([n]TeH) have enhanced infrared absorption compared with their sulfur and selenium counterparts. The radical cations are expected to be non‐dimerizing in DCM solvent. Benchmarking studies precisely predict the HOMO–LUMO gaps, underscoring Te‐based helicenes as viable contenders for next‐generation organic materials.

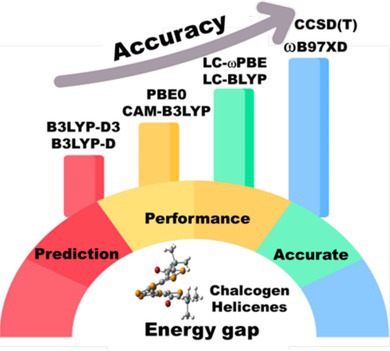

## 1 Introduction

Several optical devices use thiophene/selenophene‐conjugated oligomers and polymers because they have the appropriate energy gap, optical properties, and solar cell efficiency [1, 2, 3, 4, 5]. These systems with extended π‐conjugations with rigid and planar structures are most common [6, 7, 8, 9, 10, 11]; however, flexible and helical ones are less common [3, 4, 5, 12, 13, 14, 15]. Flexible systems adopt nonplanar helix‐type structures for ortho‐fused aromatic rings due to steric hindrance at terminal positions, for instance, thia[7]helicene [12]. Generally, adding tellurium atoms instead of S/Se atoms can make the electronic properties better suitable for device applications. Further, adding the tellurophene motif to a Te‐conjugated framework could make more advanced Te‐based organic materials. For instance, substituting Te atoms for S‐atoms lowers the energy gap in polythiophene from 2.0 to 1.5 eV [16, 17]. Tellurophene‐based conjugated systems may have improved photon absorption due to strong interactions with tellurium. Tellurophene‐based conjugated systems hold significant potential as materials for the development of photovoltaic devices. However, synthetic routes for tellurophene analogs are underdeveloped, limiting the establishment of their structure–property relationships. Te‐atom has multiple stable oxidation states; thus, tellurophene‐based systems are sensitive to the oxidizing environment [17, 18]. Furthermore, the experimentalist faces challenges in determining the energy gaps, which are crucial before embarking on costly synthesis routes for these systems. In this regard, prior knowledge of HOMO–LUMO gaps in such systems can accelerate the process for finding suitable candidates for photovoltaic applications. Accurate prediction of HOMO–LUMO gaps in such systems becomes challenging with longer chain lengths, even for computational chemists. This is because several DFT functionals can produce inaccurate results. Many reports have suggested the use of conventional functionals, mostly B3LYP, within the Kohn–Sham density functional theory [19, 20]. B3LYP functional has become an obvious choice due to its ability to handle large molecules at a reasonable computational cost. But common DFT functions like B3LYP struggle with self‐interaction and fail to make sufficient long‐range corrections, potentially leading to inaccurate predictions in organic photovoltaics. In general, functionals such as B3LYP‐D and B3LYP‐D3 can show a reasonable improvement compared with the B3LYP functional in terms of predicting the accurate energy gap. This is due to the incorporation of dispersion interactions in B3LYP‐D and B3LYP‐D3 functionals. Furthermore, long‐range corrected functionals M06 and MN15 are better choices for determining the HOMO–LUMO gap compared with the B3LYP functional. However, they incorporate a moderate to high percentage of HF exchange. Recently, range‐separated density functionals have gained attention in reproducing orbital energies in comparison to conventional ones [21, 22, 23, 24, 25, 26]. Different computational studies suggest different functionals for the accurate prediction of the HOMO–LUMO gap. Recent studies have revealed that the ωB97X‐V functional can replicate experimental energy gaps for various oligomers used in organic solar cells. Further, an optimally tuned ωB97XD functional, tuned to satisfy the Koopmans' theorem at the HOMO and LUMO energy levels, can offer better accuracy in HOMO–LUMO gap predictions. But the ωB97XD functional is computationally expensive and often encounters convergence problems for large systems [27, 28, 29, 30]. The double‐hybrid functional B2PLYP [31] can be highly effective, but computationally expensive in predicting electronic properties of conjugated systems. HSE06 [32, 33] functional is highly effective for materials requiring precise band gap estimations, such as thiophene‐ and selenophene‐based systems. However, benchmarking can be misleading when experimental energy gaps are not available. In the absence of experimental results, the values of energy gaps at the CCSD(T) (also known as the ‘gold standard method of quantum chemistry’) method are considered very accurate. Thus, a comprehensive study that involves benchmarking of DFT functionals for energy gaps is always of prime importance in such systems.

Dimerization of monomer aromatic units in solution and solid state, as well as poor solubility with increasing chain length, are the main concerns for these types of conjugated materials. Dimerization in oligothiophene, oligopyrrole, and pleiadiene radical cations is also observed only in solid state or concentrated solutions at low temperatures [34, 35, 36, 37]. Chemists have utilized sterically controlled annelation techniques with bicyclo[2.2.2]octene units to overcome the dimerization [38, 39]. Further, the use of large substituents and solubilizing substituents like trimethylsilyl (‐TMS) and triisopropylsilyl (‐TIPS) at terminal positions is also suggested to render the process of dimerization. Inspired by previous strategy, an extraordinarily stable thia[7]helicene system (~11 h) at room temperature was reported by Rajca et al. [12]. In addition, a very stable thia[7]helicene radical cation is reported. Exceptional stability in these kinds of systems is suggested based on their helical shape and bulky terminal substitutions [40]. A theoretical model study suggests that п‐interaction in monomer units decreases in thia[7]helicene radical cation due to ‐TMS and ‐Br substitutions at terminal positions in dichloromethane (DCM) solvent [6]. Recently, our investigation of thia[7]helicene and selno[7]helicene radical cations also suggests the use of ‐TMS and ‐Br substitutions at terminal positions in DCM solvent for enhanced stability compared with their unsubstituted analogs [13, 14].

The research focuses on benchmarking HOMO–LUMO gaps from different DFT methodologies, advancing computational techniques for predicting HOMO–LUMO gaps for thiophene[*n*]helicenes, seleno[n]helicenes, and telluro[n]helicenes ([n]TeH). After the establishment of the DFT method for accurate prediction of HOMO–LUMO gaps in these helicenes, the structural and energetic properties of neutral and radical cations, a dimerization study on unsubstituted and substituted telluro[7]helicene radical cations, and the calculated UV–visible spectra for [n]TeH are further reported. The study also compares results with existing computational and experimental studies on electronic features of thiophene‐ and selenophene‐based analogs. This benchmarking study for the HOMO–LUMO gap, comprehensive DFT‐based analysis on substituted [n]TeH, and comparison with their S‐ and Se‐counterparts will inspire synthetic chemists and experimentalists to investigate these systems for potential device applications.

## 2 Theoretical Methods

Twelve density functionals are used for benchmarking HOMO–LUMO energies, which are BLYP, B3LYP [41, 42], M06 [43], MN15 [44], PBE0 [45], B3LYP‐D [46], B3LYP‐D3 [47], LC‐BLYP [48], CAM‐B3LYP [49], LC‐ωPBE [50], ωB97XD [51], and B2PLYP [31]. Literature suggests that the Los Alamos ECP, LANL2DZ [52, 53] basis set accurately predicts structural features of tellurophene‐based systems [19, 54], which is used for Te atoms and 6–311++G(d,p) for other elements at present. The electronic properties for Te‐based helicene systems calculated using the def2‐TZVP [55] basis set in conjunction with DFT methods can produce qualitatively satisfactory results, but are computationally expensive. However, the LANL2DZ basis set offers computational efficiency for larger Te‐based helicene systems without compromising much on the accuracy of HOMO–LUMO energy gaps. First, geometries of neutral Thia[n]helicenes, *n* = 1–5, Seleno[n]helicenes, *n* = 1–4 and [n]TeH, *n* = 1–4 systems are optimized at the CCSD/6–311++G(d,p) level, then the CCSD(T) energies were computed for each of the neutral, cationic, and anionic species. To the best of our knowledge, the experimental ionization energies and electron affinities of such systems are not documented in the literature. The ionization energy (*IE*) is calculated from the CCSD(T) energy difference of cationic and neutral species $\left(\mathrm{IE}=E_{\text{CCSD}\left(T\right)}^{\text{cationic}}-E_{\text{CCSD}\left(T\right)}^{\text{neutral}}\right)$, while the electron affinity (*EA*) is calculated from the CCSD(T) energy difference of neutral and anionic species $\left(\mathrm{EA}=E_{\text{CCSD}\left(T\right)}^{\text{neutral}}-E_{\text{CCSD}\left(T\right)}^{\text{anionic}}\right)$. The difference in CCSD(T) *IE* and CCSD(T) *EA*, that is, $\left({∆}_{\text{CCSD}\left(T\right)}=\mathrm{IE}-\mathrm{EA}\right)$ is the fundamental gap, considered as the standard data in the absence of the experimental gap. For this study, we have considered two sets of methodology: one where the geometry optimization is done with B3LYP, and the other where the optimization is done with their respective functionals. The DFT HOMO–LUMO gap $\left({∆}_{\mathrm{DFT}}=\varepsilon_{\mathrm{DFT}}^{\text{LUMO}}-\varepsilon_{\mathrm{DFT}}^{\text{HOMO}}\right)$ of the neutral species is simply the difference in the HOMO and LUMO eigenvalues. The statistical errors, mean signed error (MSE), mean absolute error (MAE), and the maximum unsigned error (MAX_UE) are calculated and analyzed. Two sets of DFT calculations are performed on Thia[n]helicenes, *n* = 1–5, Seleno[n]helicenes, *n* = 1–4, and [n]TeH, *n* = 1–4 systems to compute the orbital energies. In the first set (Set1) of functional (BLYP, B3LYP, PBE0, MN15, B3LYP‐D, B3LYP‐D3, LC‐BLYP, HSE06, B2PLYP, and ωB97XD), the geometry optimization is also done with the same DFT functional. In the second set (Set2) of functional (M06, CAM‐B3LYP, LC‐BLYP, LC‐ωPBE, and ωB97XD), each of the molecular systems is first optimized applying B3LYP, and a single‐point calculation is performed at each of the choices of density functionals from Set 2.

Structures of neutral and radical cations, [n]TeH, *n* = 1–10, are fully optimized at the B3LYP and B3LYP‐D levels of theory to achieve the most stable structures in both the gas phase and in the DCM solvent. DCM solvent (using macroscopic solvation model based on solute density (SMD) [56, 57]) is used for solvent‐based calculations for oligotellurophene systems [58]. The restricted open‐shell Hartree‐Fock (ROHF) formalism is adapted for radical cationic doublet systems to avoid mixing of spin states. UV–visible absorption spectra of neutral and radical cations (lowest 50 excited states) are calculated following the TDDFT procedure and considering the CAM‐B3LYP [59, 60] and ωB97XD functionals in DCM solvent. A case study on π‐dimerization is also reported for the unsubstituted and end‐substituted dimers telluro[7]helicene radical cation in DCM solvent. All theoretical calculations are performed using the GAUSSIAN 16 program [61].

## 3 Results and Discussion

### 3.1 Structural Parameters of Telluro[n]Helicenes ([n]TeH, *n* = 1–10) and Their Corresponding Radical Cationic Systems

**FIGURE 1:**
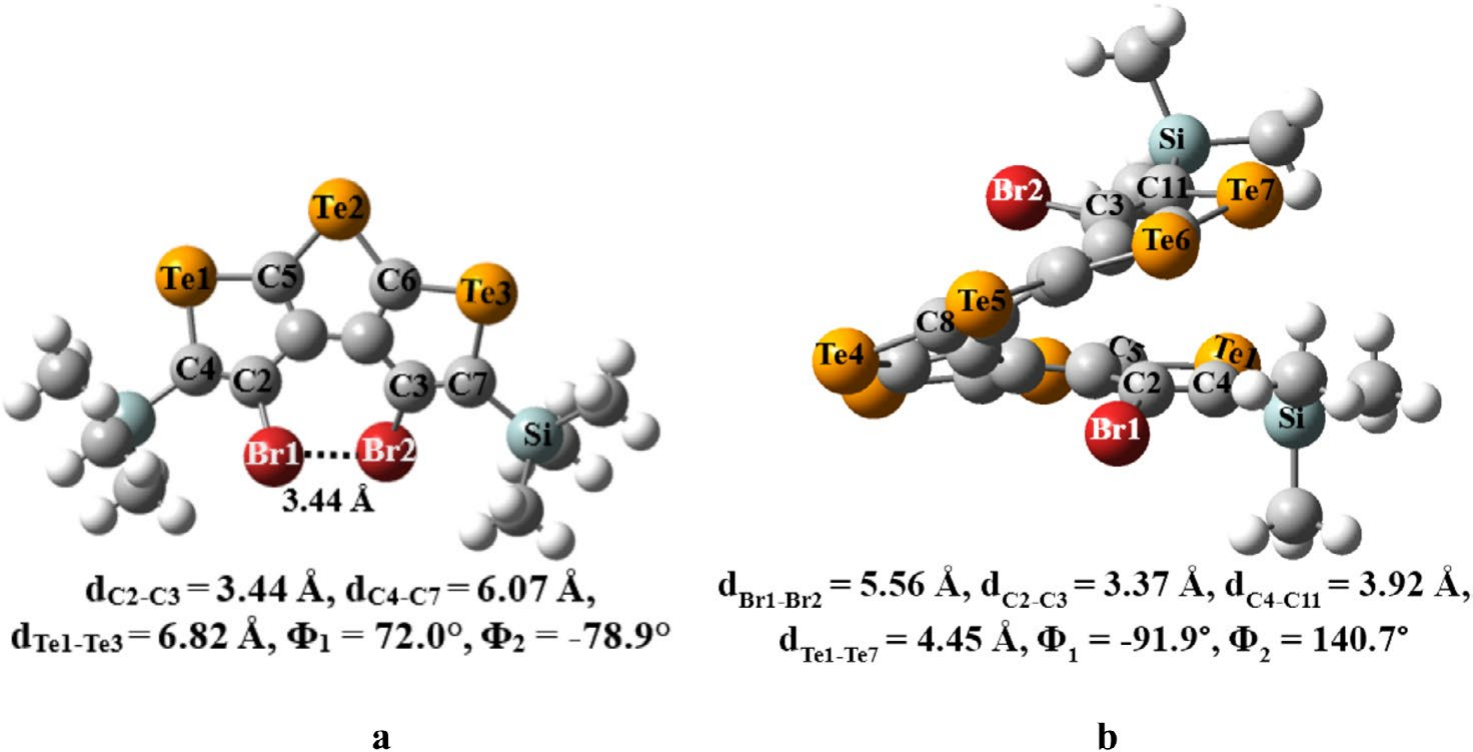
Optimized structures of (a) telluro[3]helicene and (b) telluro[7]helicene Φ_1_ = δ(Br1C2C3Br2) and Φ_2_ = δ(Br2C3C2C4) computed using B3LYP‐D functional in DCM solvent. Basis set used: 6–311++G(d,p) for H, C, Si, Br atoms, and LANL2DZ for Te atoms. Selected bond distances and dihedral angles are labeled in the figures.

**FIGURE 2:**
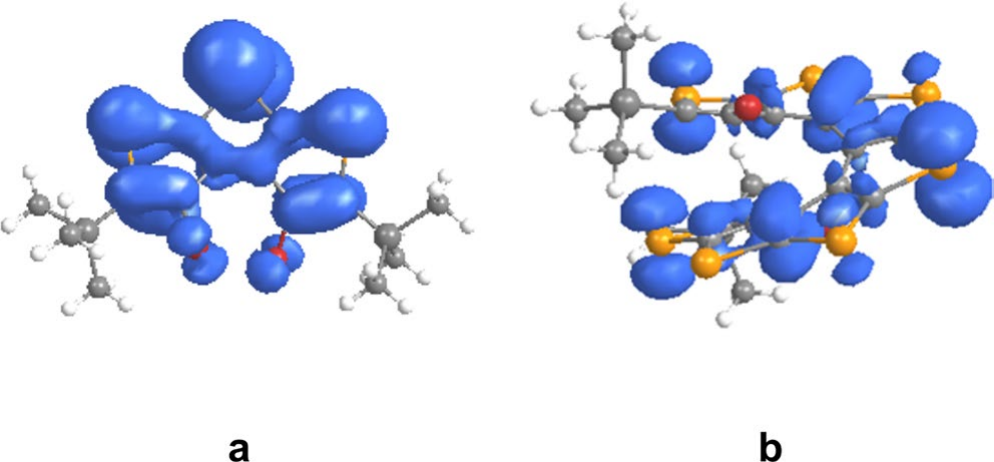
Spin density map for the most stable structures of (a) [3]TeH^•+^ and (b) [7]TeH^•+^. DFT functional used: B3LYP‐D (ROHF formalism); basis set used: 6–311++G(d,p) for H, C, Si, Br atoms, and LANL2DZ for Te atoms. Solvent used: DCM (SMD model). Iso‐value = 0.003 a.u.

**FIGURE 3:**
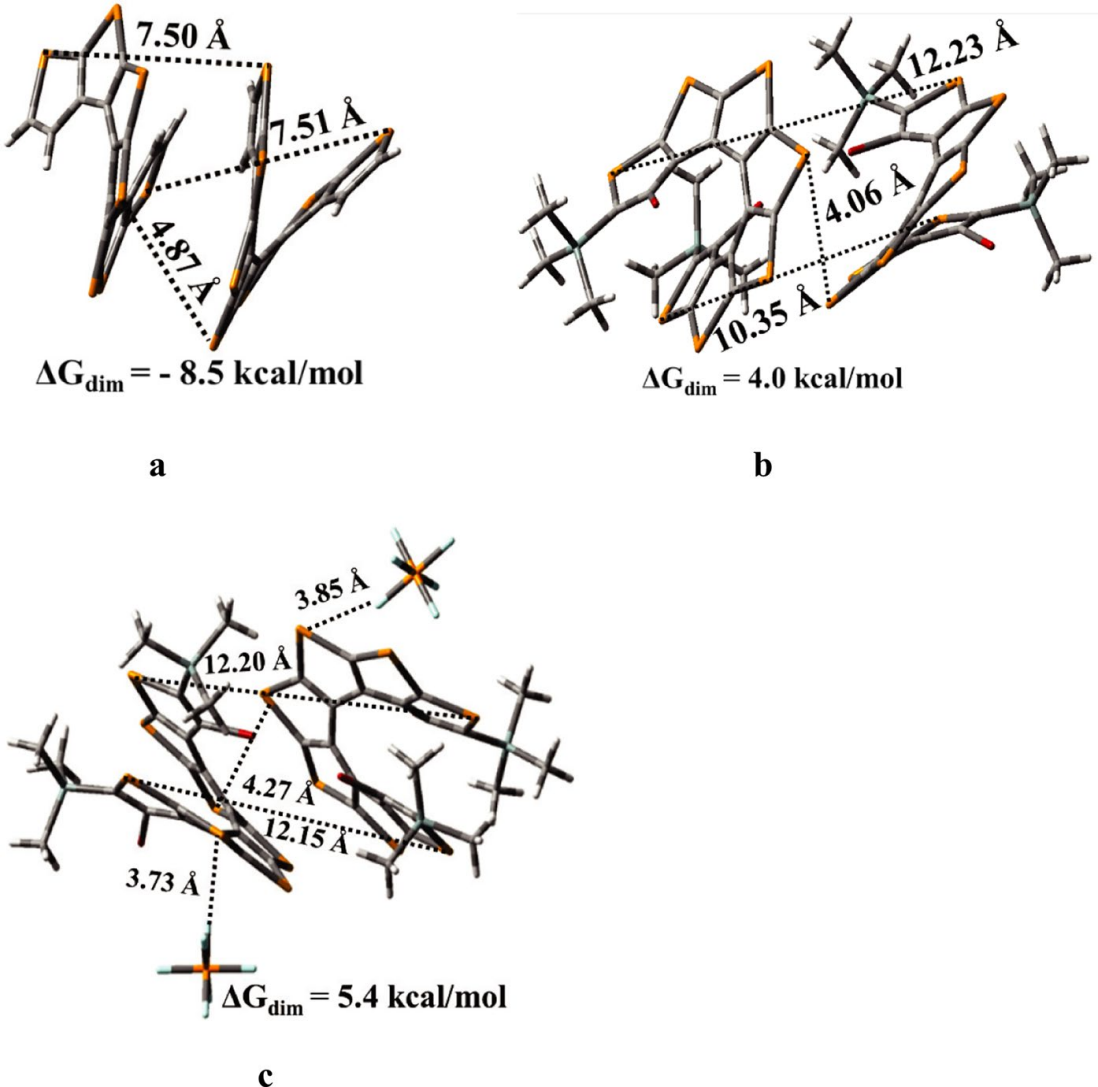
Most stable π‐dimeric structures of (a) unsub‐([7]TeH)_2_^2+^, (b) sub‐([7]TeH)_2_^2+^, and c) sub‐([7]TeH)_2_^2+^ ∙ ((PF_6_)_2_^2−^ calculated using B3LYP‐D functional. Basis set used: LANL2DZ basis set for Te atoms and 6–311++G(d,p) basis set is for all other atoms. The solvent of DCM is incorporated by considering the macroscopic SMD model. Unsub indicates that the system is not end‐substituted, and sub indicates that the system is substituted by ‐Br (β, β’) and ‐TMS (α, α’) at terminal tellurophene rings.

Cartesian coordinates of the equilibrium geometries of [n]TeH obtained at the B3LYP‐D functional with 6–311++G(d,p) basis set for H, C, Si, Br atoms and LANL2DZ for Te atoms in DCM solvent are provided in the Supporting Information. To put it simply, the overall shapes of neutral [n]TeH (*n* = 1–10) in gas phase and DCM solvent are pretty much similar at the B3LYP and B3LYP‐D levels of theory with 6–311++G(d,p) basis set for H, C, Si, Br atoms, and LANL2DZ for Te atoms basis set. However, we observed only small changes to the geometrical parameters for [n]TeH. All reported structures of these tellurophene‐based helicenes ([n]TeH) are closely related to their corresponding S‐, [n]TH, and Se‐based, [n]SH analogs. The most stable structures of tellurophene‐based [n]helicenes, *n* = 1–2, are planar with C_2h_ symmetry, and with *n* > 2 are nonplanar with C_2_ symmetry. The nonplanar structures of [n]TeH for *n* > 2 result from the repulsive interaction between large substituents present at the terminal tellurophene rings. Comparison between the dihedral angles calculated at B3LYP and B3LYP‐D levels of theory shows that repulsive interaction between large groups at terminal rings requires a dispersion‐corrected version of the B3LYP functional. However, end‐to‐end Te‐Te non‐bond distances and nonbonded Br‐Br distances are hardly different at B3LYP and B3LYP‐D levels of theory (see Table S11). The calculated dihedral angles, Φ_1_ and Φ_2_, are 72.0° and −78.9°, respectively, for the nonplanar structure of [3]TeH in DCM solvent as shown in Figure 1a. The X‐ray reported structure for thia[3]helicene [12] has Φ_1_ and Φ_2_ values 13.3^o^ and −164.3^o^, respectively, while for the computationally reported seleno[3]helicene [14] system, the Φ_1_ and Φ_2_ values are 47.3^o^ and −128.3^o^, respectively. Mulliken charges over Te1, Te2, Si, and Br atoms are 0.80, 0.82, 0.07, and −0.11 a.u., respectively. The calculated charges on Br atoms are much more negative in telluro[3]helicene compared with its S‐ and Se‐based analogs. It appears that the nonplanarity in telluro[3]helicene should be more than in its S‐ and Se‐based analogs. Further, the calculated non‐bond distances, d_S1‐S3_, d_Se1‐Se3_, and d_Te1‐Te3_ are 5.82, 6.62, and 6.82 Å, respectively, for [3]TH [13], [3]SH [14], and [3]TeH systems in DCM solvent. The reported dihedral angles, −170.11 and 83.11, for the thia[7]helicene system based on crystallography [12]. In the case of thia[7]helicene, the dihedral angles Φ_1_ and Φ_2_ are −166.1° and 82.6°, respectively, at the B3LYP‐D/6–311++G(d,p) level of theory in DCM solvent. The dihedral angle values are −170.1^o^ and 83.1^o^ for thia[7]helicene system based on crystallography [12]. For computationally reported seleno[7]helicene [14] system, the Φ_1_ and Φ_2_ values are −128.9^o^ and 108.5^o^, respectively. The calculated dihedral angles Φ_1_ and Φ_2_ values for the telluro[7]helicene system are −91.9^o^ and 140.7^o^ (see Figure 1b) at B3LYP‐D level in DCM solvent. The telluro[7]helicene ([7]TeH) system can be more conjugated than its sulfur ([7]TH) and selenium ([7]SH) analogs, as evidenced by its dihedral angles are closer to planarity compared with [7]TH and [7]SH. Further, [7]TeH has a shorter nonbonded distance (d_Te1‐Te7_ = 5.30 Å) than [7]TH (d_S1‐S7_ = 8.00 Å) and [7]SH (d_Se1‐Se7_ = 6.63 Å), along with the higher polarizability of tellurium, which can enhance π‐electron delocalization and conjugation. These telluro[n]helicene systems require seven tellurophene units for a full turn (see Figure 1b), one unit lower than their S‐ and Se‐based analogs. The remaining most stable structures, along with structural information for telluro[n]helicene (*n* = 1–2, 4–6, 8–10), are provided in the Supporting Information (Figure S1a–h, Table S1). The most stable structures obtained for [n]TeH^•+^, *n* = 1–10, are quite similar to their corresponding neutral [n]TeH at the present level of theory in DCM solvent (see Table S1). In [3]TeH^•+^, Br atoms experience a significant population of spin densities in DCM solvent, as one can notice from the Figure 2. These most stable structures, along with structural information for telluro[n]helicene radical cations (*n* = 1–10), are provided in the Supporting Information (Figure S2a–j and Table S1) along with their Cartesian coordinates. The spin density plot of telluro[3]helicene radical cation is calculated using its most stable structure at the B3LYP‐D level of theory under ROHF formalism in DCM solvent (Figure 2a). The spin densities at Te1 and Te2 atoms are 0.11 and 0.40 a.u., respectively, while at carbon atoms, they are 0.05 a.u. The central tellurophene ring and adjacent rings delocalize the spin populations. Central and terminal rings of [7]TeH^•+^ in DCM primarily distribute spin densities over Te atoms (Figure 2b). The EPR spectra of the [7]TeH^•+^ radical cation may not have a *g* value similar to the free electron value in the DCM solvent. The remaining spin density plots for [n]TeH radical cation are provided in the Supporting Information (*n* = 1–2, 4–6, 8–10 systems, Figure S3a–h). Thus, the B3LYP‐D functional can provide better descriptions of structural information for [n]TeH systems in neutral and positively charged states compared with the B3LYP functional. Neutral and radical cationic states of [n]TeH systems share almost similar geometrical parameters and are closely related to their S‐ and Se‐analogs. Telluro[n]helicene systems show a higher degree of conjugation compared with their S‐ and Se‐counterparts. Thus, lower *IE* is required in converting neutral systems to their corresponding positively charged ions in [n]TeH in comparison to their S‐ and Se‐counterparts in DCM solvent (see Figure S4). The values of ionization energies decrease by 1.1 to 1.9 eV in DCM solvent as compared with the gas phase in [n]TeH systems (see Table S2). Higher conjugation in [n]TeH can significantly increase the likelihood of dimerization as compared with their S‐ and Se‐counterparts. Experimentally, one‐electron oxidation of end‐substituted thia[7]helicene generated its radical cation in DCM solvent. A theoretical model study based on Gibbs free energy calculations revealed the high thermodynamic stability in solution for radical cation of end‐substituted thia[7]helicene [13] and its selenophene‐analogs [14]. The model study on one‐electron oxidation of telluro[7]helicene is also needed to compare their structural and energetic properties with their corresponding S‐ and Se‐analogs. In this regard, we conducted a case study on unsubstituted to substituted telluro[7]helicene radical cation. Out of several possible structures of unsubstituted and substituted telluro[7]helicene radical cations (represented as unsub‐([7]TeH)_2_^2+^ and sub‐([7]TeH)_2_^2+^), the most stable structures are provided in Figure 3a to Figure 3b. The Δ*G*_dim_ value for π‐dimer of unsub‐([7]TeH)_2_^2+^ (see Figure 3a for structure) is −9.2 kcal/mol, which is significantly more negative than sub‐([7]TeH)_2_^2+^ (+4.0 kcal/mol) π‐dimeric system (see Figure 3b for structure). It is found that the bulky substituents (‐Br and ‐TMS groups) at terminal positions in tellurophene rings create steric hindrance in sub‐([7]TeH)_2_^2+^ π‐dimer, and that leads to a decrease of π‐π stacking in sub‐[7]TeH units. The decrease in π‐π stacking for sub‐([7]TeH)_2_^2+^ π‐dimer is reflected as a more positive value of Δ*G*_dim_ as compared with the π‐dimer of unsub‐([7]TeH)_2_^2+^. To consider the effect of counter ion, two PF_6_^−^ anions are placed in various positions in the most stable structure of the ([7]TeH)_2_^2+^ dimeric system, and geometry optimization is carried out. Three main guess structures with two counter ions are, (1) the two PF_6_^−^ anions are placed at the bottom and top parallel to the coil axis, (2) both the PF_6_^−^ anions are placed perpendicular to coil axis, and (3) two PF_6_^−^ anions is placed near one coil only to [7]TeH units.

In the presence of two PF_6_^−^ counter ions, the ΔG_dim_ value for the π‐dimer of sub‐([7]TeH)_2_^2+^ ∙ (PF_6_)_2_^2−^ (see Figure 3c for the final structure) is +5.4 kcal/mol, which is more positive than the sub‐([7]TeH)_2_^2+^ (+4.0 kcal/mol) π‐dimeric system. The dispersion interactions are weakened in the π‐dimeric system of sub‐([7]TeH)_2_^2+^ ∙ (PF_6_)_2_^2−^ and π‐dimeric system becomes less stable compared with sub‐([7]TeH)_2_^2+^ due to the presence of anionic counter ions. Thus, it is expected that the radical cationic systems of substituted [n]TeH to have stability like their S‐ and Se‐based helical analogs. Cartesian coordinates of the most stable structures from Figure 3a (unsub‐([7]TeH)_2_^2+^), Figure 3b (sub‐([7]TeH)_2_^2+^), and Figure 3c (sub‐([7]TeH)_2_^2+^ ∙ (PF_6_)_2_^2−^) are provided in the Supporting Information.

### 3.2 Benchmarking Studies of DFT Functionals for HOMO–LUMO Gap and Comparative Study With Thio‐, Seleno‐, and Telluro‐ Helicenes

**FIGURE 4:**
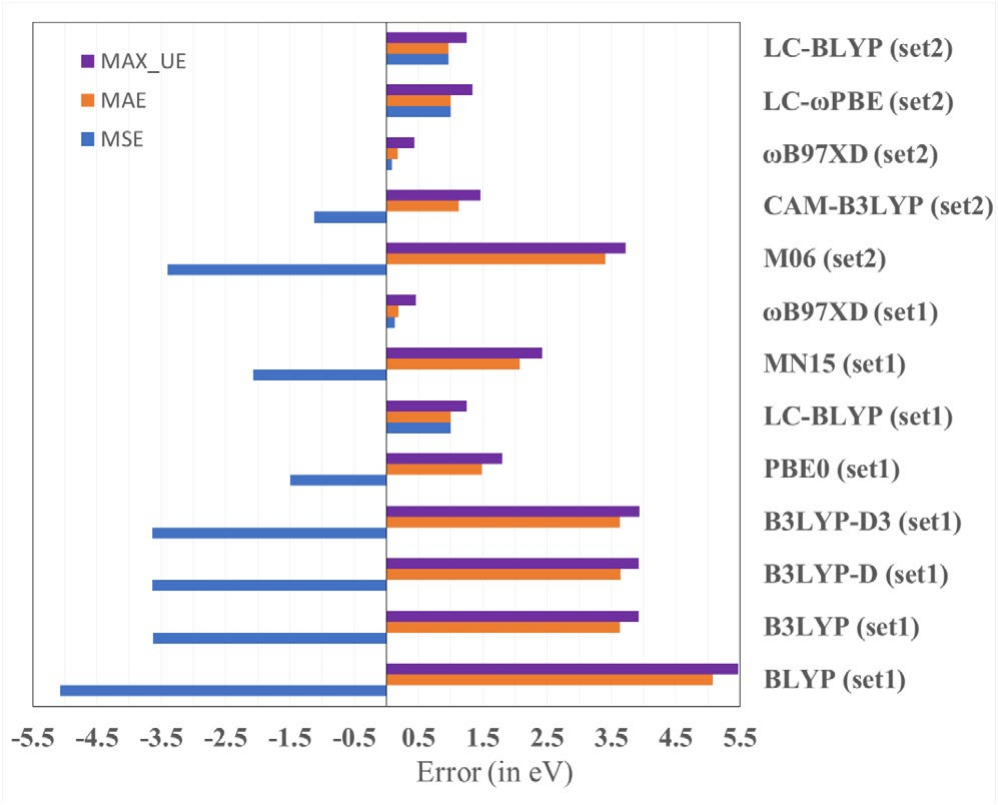
Error in HOMO–LUMO energy gap (in eV) calculated by different density functionals for Thia[n]helicene, *n* = 1–5, Seleno[n]helicene, *n* = 1–4 and Telluro[n]helicene, *n* = 1–4 systems compared with CCSD(T) results. Errors (mean signed error (MSE), mean absolute error (MAE), and maximum unsigned error (MAX_UE)) are calculated for the two sets (Set1 and Set2) used for our analysis.

**FIGURE 5:**
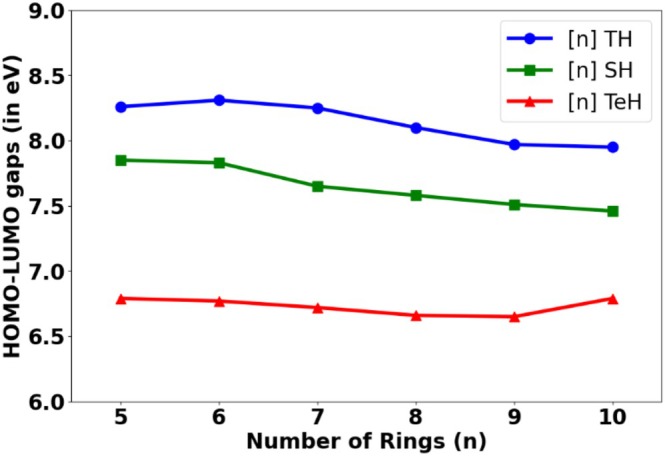
Plot of HOMO–LUMO gaps versus the number of rings (*n*) in thia[n]helicenes, [n]TH, *n* = 5–10, and seleno[n]helicenes, [n]SH, and telluro[n]helicenes, [n]TeH, *n* = 5–10 in gas phase: DFT Method: B3LYP functional (most stable geometries) followed by single‐point energy calculations at ωB97XD functional. Basis set used: 6–311++G(d,p) for H, C, Si, S, Se, Br atoms, and LANL2DZ basis set for Te atoms.

Computed orbital energies with the GGA kind of functional, BLYP, are very high from the benchmark values. The MAE for the BLYP functional is more than 5 eV (see Figure 4 and Table 1). Employing a hybrid functional improves the orbital energies to some extent, but, in general, it is not a correct one for computing orbital energies. For instance, the B3LYP functional, which is a default choice of many experimentalists, also supports the measurement outcome, giving a MAE of 3.6 eV with a maximum error of 4 eV. For experimental chemists, such a high value error of B3LYP would be misleading for systems with a low HOMO–LUMO gap, particularly in systems with increasing chain length. The dispersion correction will have a minimum contribution to accuracy compared with the form of the functional, as may be expected. In this case, it is observed that the dispersion‐corrected functional (B3LYP‐D and B3LYP‐D3) has no significant impact on the accuracy of the orbital energies, and hence HOMO–LUMO energy gaps. Surprisingly, the other hybrid functional, PBE0 (only S and Se), shows a much‐improved HOMO–LUMO gap (1.4 eV) than the B3LYP functional. HOMO–LUMO energy gaps calculated applying HSE06 functional are underestimated by approximately 0.50 to 1.30 eV for these systems (Table S12). Thus, the performance of HSE06 functional for HOMO–LUMO energy gaps is expected to be better than BLYP. HOMO–LUMO energy gaps at the Moreover, the performance of the M06 functional (in Set 2) is slightly improved (3.4 eV) over B3LYP, which may be due to a different level of optimization employed. A much‐improved HOMO–LUMO gap (2 eV) is observed for the latest series of Minnesota functional MN15. On the other HSE06 functional is underestimated as compared with the HOMO–LUMO energy gaps obtained at the B3LYP functional hand, it is interesting to note that the long‐range corrected density functionals provide an acceptable deviation from benchmark values. In general, it can also be observed that the long‐range corrected density functionals consistently overestimate the HOMO–LUMO gap. It may be noted that the functional ωB97XD, in both Set1 and Set2, proves to be the best among the sets, with a remarkable overall error to be within 0.17–0.19 eV. The other long‐range corrected density functionals, LC‐BLYP, LC‐ωPBE, and CAM‐B3LYP, reproduce the HOMO–LUMO gap within 1 eV. Moreover, on comparing the Set1 and Set2 data, it may be inferred that slightly better HOMO–LUMO gaps are found if the optimization is done in B3LYP (Set2) than in the functional itself (Set1). Although the calculations are on a set of similar systems, it may be anticipated that the prescription of computing the orbital energies with ωB97XD (*ω* = 0.20 bohr^−1^) will hold for other systems as well. Computed HOMO–LUMO gaps at B2PLYP functional, including Grimme's D3 dispersion correction, are quite like HOMO–LUMO gaps at CAM‐B3LYP for thia[n]helicenes, seleno[n]helicenes, and [n]TeH for *n* = 1–5. LC‐ωPBE functional is built upon with the default range‐separation parameters, *ω* = 0.40 bohr^−1^ [62a]. The value of ω is varied for the LC‐ωPBE functional to understand the change in the HOMO–LUMO gap in the case of telluro[1]helicene. The optimal tuned ω value is obtained by minimizing the J, which is (𝜀_HOMO_ + IP)^2^ with respect to ω to satisfy the Koopmans' theorem [62b, 62c]. Optimally tuned ω value for LC‐ωPBE functional for telluro[1]helicene is 0.21 bohr^−1^ in gas phase. LANL2DZ basis set for Te atoms and 6–311++G(d,p) basis set for all other atoms are used for optimal tuning of ω value. The ω value for LC‐ωPBE functional is close to the default ω value for ωB97XD functional. The optimally tuned ω value at LC‐ωPBE functional for telluro[4]helicene is 0.19 bohr^−1^, which is marginally lower than the optimally tuned ω value for telluro[1]helicene and consistent with some reported systems [62d, 62e]. The optimized ω parameter trend is consistent with the fact that ω value decreases as the size of the system increases [62b, 62c]. A smaller range‐separation value of ω in ωB97XD functional than the default ω value in LC‐ωPBE functional may be responsible for accurate HOMO–LUMO gap predictions applying ωB97XD functional, which is in line with available literature [62, 63]. Further, optimally tuned ω values for telluro[1]helicene and telluro[4]helicene are 0.17 and 0.14 bohr^−1^, respectively, in gas phase for ωB97XD functional. The plot showing variation of 𝐽 ((𝜀_HOMO_ + IP)^2^) with ω for telluro[1]helicene and telluro[4]helicene are provided in Supporting Information. The calculated density of states spectra using single‐point energy calculations at ωB97XD functional with the most stable geometries obtained with B3LYP functional for [n]TeH (*n* = 1–10) are presented in Figure S5. It is also found that HOMO–LUMO gap for [*n*]TeH calculated applying def2‐TZVP basis set for Te atoms and 6–311++G(d,p) basis set for all other atoms are quite close to the results obtained with LANL2DZ basis set for Te atoms and 6–311++G(d,p) basis set for all other atoms. Calculated orbital energies are provided in the Supporting Information from Tables S3–S16. Out of the 15 tested methodologies, it is found that an accurate HOMO–LUMO energy gap is obtained using the ωB97XD functional, preferably with optimization done at the same level of theory. Overall, a lower computation cost is required when HOMO–LUMO energy gaps are calculated using single‐point energy calculation at the ωB97XD functional with geometry optimization performed with B3LYP level of theory. Table S15 in Supporting Information compares the computational cost. DFT calculations highlight that the HOMO–LUMO gaps of Te‐analogs are redshifted compared with their S‐ and Se‐analogs (Figure 5) for medium to large‐ring‐size helicenes (*n* ≥ 5) with ωB97XD functional. The HOMO–LUMO gap values for medium to large‐ring‐size helicenes are listed in Table 2. The documented HOMO–LUMO gap for thia[n]helicene (*n* = 1–10) and seleno[n]helicene (*n* = 1–10) at B3LYP, B3LYP‐D, and B3LYP‐D3 using the 6–311++G(d,p) basis set in gas phase is consistently comparable in a solvent such as DCM, according to the literature [13, 14]. In literature, the electronic absorption spectra of [3]TH and [7]TH (TH stands for thio‐helicene) in DCM are reported [64]. The excited‐state spectra of [3]TH and [7]TH are calculated using CAM‐B3LYP, ωB97XD, and LC‐ωPBE functionals applying TDDFT procedure and 6–311++G(d,p) basis set in DCM solvent. The *λ*_max_ values obtained with CAM‐B3LYP and ωB97XD functionals closely matched the experimental values, while LC‐ωPBE predicted lower *λ*_max_ values (see Supporting Information). Therefore, the default *ω* value in ωB97XD can be trusted for calculating the HOMO–LUMO energy gap in DCM for these systems. The optimal tuned *ω* values for telluro[1]helicene and telluro[4]helicene in DCM solvent are 0.04 bohr^−1^ (~5 times lower than the gas phase *ω* value) and 0.02 bohr^−1^ (~10 times lower than the gas phase *ω* value), respectively. The trend observed for ω values in solvents with increasing size is consistent with the reported literature [65]. In DCM solvent, optimally tuned ω values for telluro[1]helicene and telluro[4]helicene are 0.018 and 0.012 bohr^−1^, respectively, for ωB97XD functional. The plot showing variation of 𝐽 ((𝜀_HOMO_ + IP)^2^) with ω for telluro[1]helicene and telluro[4]helicene in DCM solvent are provided in Supporting Information.

**TABLE 1:** Statistical Errors (MSE, MAE, and MAX_UE)^a^ in eV for different density functionals for Thia[n]helicene, *n* = 1–5, Seleno[n]helicene, *n* = 1–4 and Telluro[n]helicene, *n* = 1–4 systems with respect to CCSD(T) results.

Functional	MSE	MAE	MAX_UE
BLYP (set1)	−5.076	5.076	5.472
B3LYP (set1)	−3.631	3.631	3.926
B3LYP‐D (set1)	−3.638	3.638	3.929
B3LYP‐D3 (set1)	−3.637	3.637	3.931
PBE0 (set1)	−1.394	1.394	1.378
LC‐BLYP (set1)	1.003	1.003	1.245
MN15 (set1)	2.073	2.073	2.421
ωB97X‐D (set1)	0.137	0.189	0.464
M06 (set2)	−3.405	3.405	3.722
CAM‐B3LYP (set2)	−1.125	1.125	1.463
ωB97XD (set2)	0.091	0.172	0.435
LC‐ωPBE (set2)	1.002	1.002	1.345
LC‐BLYP (set2)	0.965	0.965	1.255

^a^ MSE, MAE, and MAX_UE refer to mean signed error, mean absolute error, and maximum unsigned error, respectively.

**TABLE 2:** Calculated HOMO–LUMO gaps (E_g_), for thia[n]helicenes, [n]TH, *n* = 5–10, and seleno[n]helicenes, [n]SH, and telluro[n]helicenes, [n]TeH, *n* = 5–10, in gas phase.

Number of rings (*n*)	*E*_g_ for [n]TH (in eV)	*E*_g_ for [n]SH (in eV)	*E*_g_ for [n]TeH (in eV)
5	8.26	7.85	6.79
6	8.31	7.83	6.77
7	8.25	7.65	6.72
8	8.10	7.58	6.66
9	7.97	7.51	6.65
10	7.95	7.46	6.79

*Note:* Method: B3LYP functional (most stable geometries) followed by single‐point energy calculations at ωB97XD functional. Basis set used: 6–311++G(d,p) for H, C, Si, S, Se, Br atoms, and LANL2DZ basis set for Te atoms.

### 3.3 HDOMO–LSOMO Energy Gaps and UV–vis Electronic Absorption Spectra of Radical Cations of Telluro[n]Helicenes (*n* = 1–10) and in DCM Solvent

**FIGURE 6:**
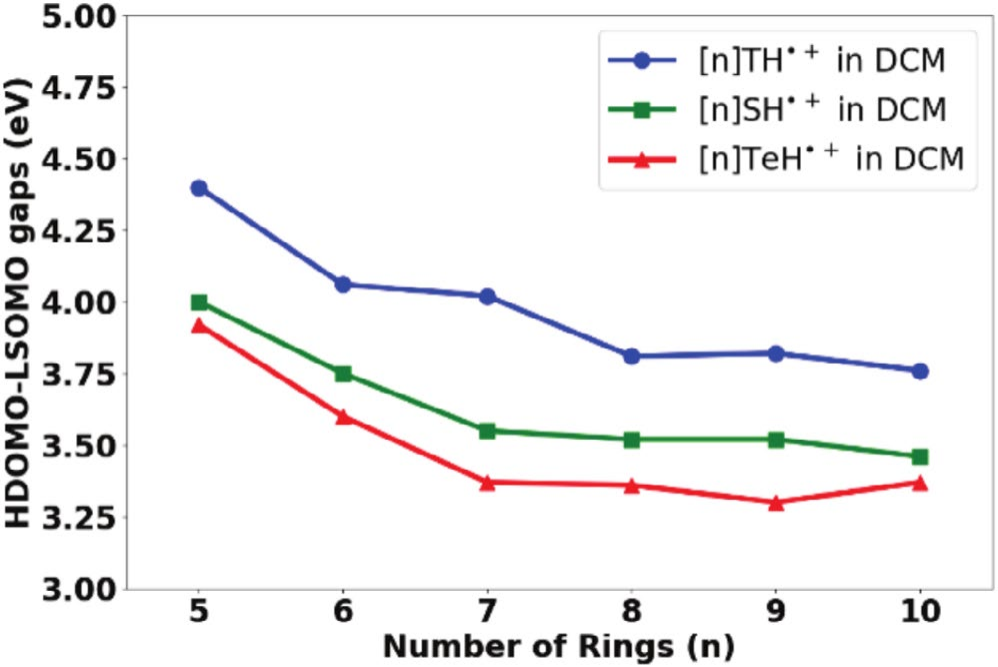
Plot of HDOMO–LSOMO gaps versus the number of rings in thia[n]helicenes radical cations, [n]TH^•+^, *n* = 5–10, seleno[n]helicenes radical cations, [n]SH^•+^, 5–10, and telluro[n]helicenes radical cations, [n]TeH^•+^, *n* = 5–10 in DCM solvent: DFT Method: B3LYP functional (most stable geometries) followed by single‐point energy calculations at ωB97XD functional. Basis set used: 6–311++G(d,p) for H, C, Si, S, Se, Br atoms, and LANL2DZ basis set for Te atoms. Solvation of DCM is accounted for by using a macroscopic SMD model. HDOMO and LSOMO stand for the highest doubly occupied orbital and the lowest singly occupied molecular orbital, respectively.

DFT calculations highlight that the HDOMO–LSOMO energy gaps of [n]TeH radical cations are redshifted compared with their S‐ and Se‐analogs (see Figure 6) for medium‐ to large‐ring‐size helicenes (*n* ≥ 5) with the ωB97XD functional, which is in similar line with trends of HOMO–LUMO energy gaps. HDOMO and LSOMO stand for the highest doubly occupied molecular orbital and the lowest singly occupied molecular orbital, respectively. The calculated HDOMO–LSOMO energy gaps are going down steadily with the ωB97XD functional for [n]TH^•+^ and [n]SH^•+^ from 5‐fused‐ring‐based helicenes to 10‐fused‐ring‐based helicenes, which is in line with reported literature [13, 14]. The values at the ωB97XD functional also support the expectation that the HDOMO–LSOMO energy gaps for [n]TeH radical cations, [n]TeH^•+^, should be lower compared with their S‐ and Se‐analogs, due to higher conjugation and the electropositive effect of Te atoms.

Also, for [n]TeH radical cations, the HDOMO–LSOMO energy gaps get smaller and smaller for helicenes with medium to large ring sizes (*n* ≥ 5). The HDOMO–LSOMO energy gap values for medium‐ to large‐ring‐size helicenes are listed in Table 3. The excited‐state properties of neutral [n]TeH and their corresponding radical cations are calculated using the TDDFT procedure. CAM‐B3LYP functional is used to calculate all the UV–Vis spectra, with LANL2DZ basis set is used for Te atoms, and 6–311++G(d,p) basis set is for all other atoms. Solvent effect in DCM is accounted for by using SMD model for macroscopic solvation. The absorption maxima (*λ*_max_), oscillator strengths, and molecular orbitals involved in electronic transitions are listed in Table S16 for neutral [n]TeH systems. Table S17 lists the *λ*_max_, oscillator strengths, and molecular orbitals involved in electronic transitions for the radical cations of tellurophene‐based [n]helicene. Simulated UV–Vis spectra of neutral [n]TeH are presented in Figures S8a and S8b. To deal with charge‐transfer states when transitioning from an isolated molecule or complex to the solid state, a smaller range‐separation parameter is recommended, as suggested by the literature [65]. To ensure the validity of the default value of range‐separation parameter, comparison of electronic absorption spectra for telluro[5]helicene and telluro[7]helicene using CAM‐B3LYP and ω‐B97XD functionals is provided in the Supporting Information. This leads to the fact that CAM‐B3LYP and ω‐B97XD functionals are quite similar in producing electronic absorption spectra for telluro[5]helicene (Figure S8c) and telluro[7]helicene (Figure S8d). The excited‐state spectra of [3]TH and [7]TH calculated using CAM‐B3LYP are closer to experimentally reported spectra. This makes CAM‐B3LYP functional as the obvious choice, and it is also reported to be among the best DFT functionals under TDDFT procedure for calculating UV–Vis spectra [59, 60]. However, ω‐B97XD functional can also be used to calculate excited‐state spectra of these kinds of systems. The *λ*_max_ values of [n]TeH in DCM solvent range from 240 to 354 nm. The neutral thiahelicene systems are expected to absorb in the ultraviolet (UV) region. The origin of optical absorption bands of the end‐substituted [n]TeH radical cation in the IR regions is due to π → π* type of electronic transition. Telluro[1]helicene radical cations exhibit a *λ*_max_ at 445 nm and a low‐intensity peak at 1174 nm in DCM solvent (Figure S9a). As the number of tellurophene rings increases, the intensity of the near‐infrared (NIR) peak and *λ*_max_ is expected to rise due to the increase in conjugation and the heavy atoms effect. Telluro[2]helicene radical cations show absorption bands at 1614 and 1045 nm, while telluro[3]helicene features strong absorption at 1047 nm and a weak peak at 2085 nm (Figure S9a).

**TABLE 3:** Calculated HDOMO–LSOMO energy gaps (in eV) for thia[n]helicenes radical cations, [n]TH^•+^, *n* = 5–10, seleno[n]helicenes radical cations, [n]SH^•+^, 5–10, and telluro[n]helicenes radical cations, [n]TeH^•+^, *n* = 5–10 in DCM solvent. Values in the braces are HDOMO–LSUMO energy in the gas phase. HDOMO and LSOMO stand for the highest doubly occupied molecular orbital and the lowest singly occupied molecular orbital, respectively.

Number of rings (*n*)	HDOMO–LSOMO energy gaps (in eV)		
	[n]TH^•+^	[n]SH^•+^	[n]TeH^•+^
5	4.40, (*5.13*)	4.00, (*4.91*)	3.92, (*4.80*)
6	4.06, (*4.71*)	3.75, (*4.50*)	3.60, (*4.42*)
7	4.02, (*4.71*)	3.55, (*4.25*)	3.37, (*4.16*)
8	3.81, (*4.48*)	3.52, (*4.26*)	3.36, (*4.22*)
9	3.82, (*4.49*)	3.52, (*4.29*)	3.30, (*4.13*)
10	3.76, (*4.28*)	3.46, (*3.33*)	3.37, (*4.19*)

*Note:* Method: B3LYP functional (most stable geometries) and then single‐point energy calculations at ωB97XD functional, Basis set used: 6–311++G(d,p) for H, C, Si, S, Se, Br atoms and LANL2DZ basis set for Te atoms. Solvation of DCM is accounted for by using the SMD model.

**FIGURE 7:**
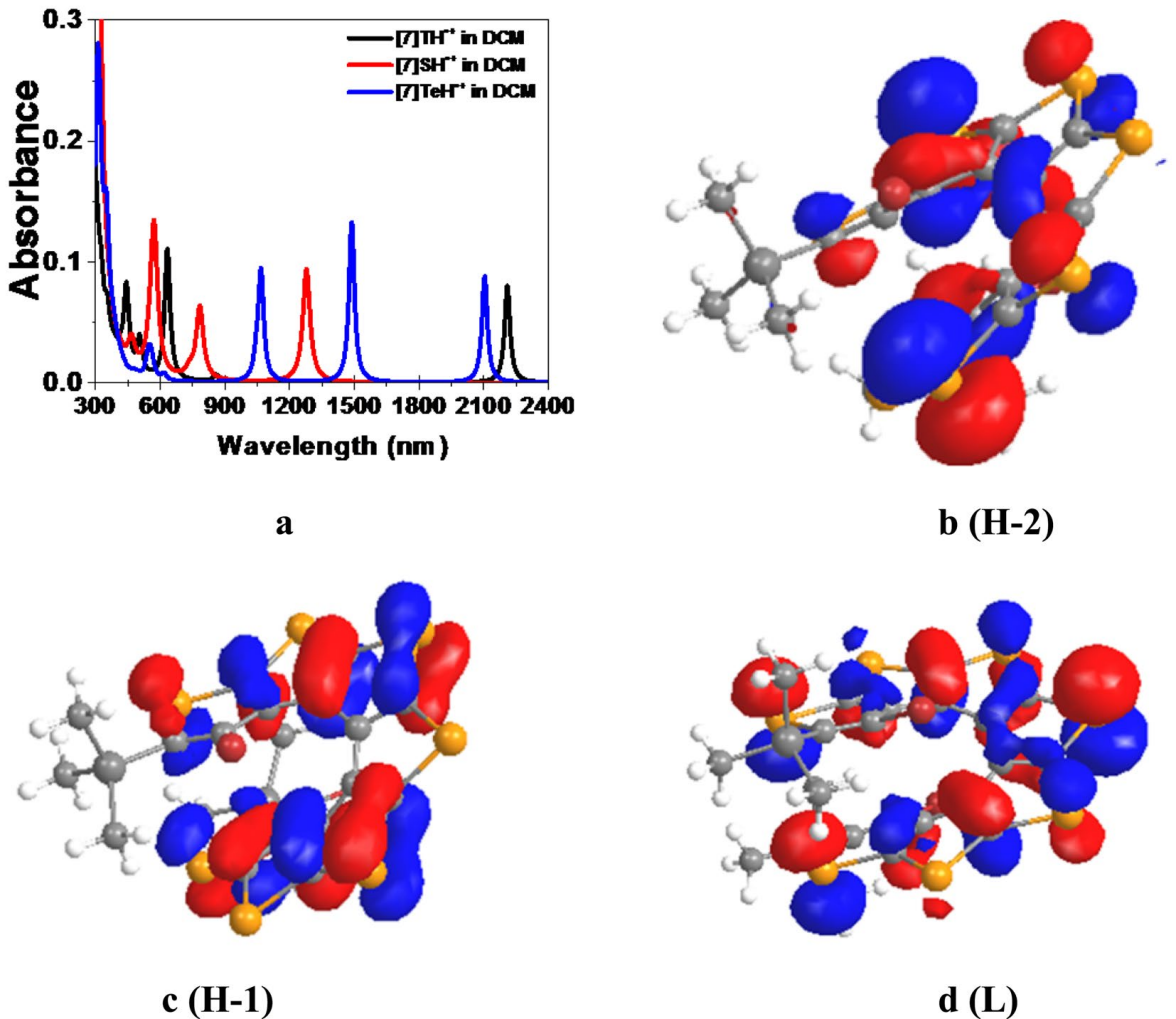
Simulated electronic absorption spectra, (a) [7]TH^•+^, [7]SH^•+^, and [7]TeH^•+^ in DCM solvent. CAM‐B3LYP functional is used to calculate all the UV–Vis spectra applying TDDFT procedure. Frontier orbitals of [7]TeH^•+^ (b) H‐2 of [7]TeH^•+^, (c) H‐1 of [7]TeH^•+^, and (d) L of [7]TeH^•+^ calculated at B3LYP‐D level of theory in DCM solvent. Frontier orbitals are taken at a contour cut‐off value of 0.03 a.u. Basis set used: LANL2DZ basis set for Te atoms and 6–311++G(d,p) basis set is for all other atoms. Solvent effect in DCM is accounted for by using the SMD model. H and L stand for the highest doubly occupied molecular orbital (HDOMO) and the lowest singly occupied molecular orbital (LSOMO), respectively.

For [4]TeH^•+^, a strong peak at 1127 nm is noted, while in [5]TeH^•+^, a strong absorption peak at 1185 nm is observed without a peak above 2000 nm. (see Figure S9a). For [7]TeH^•+^, both a weak peak at 2104 nm (H‐1 → L) and a strong peak at 1488 nm ((H‐2 → L)) are obtained, with the latter showing greater intensity compared with [7]SH^•+^ and [7]TH^•+^ (see Figure 7a). Frontier orbitals H‐2, H‐1, and L are shown in Figure 7b–d. The comparison of electronic absorption spectra [7]TeH^•+^ calculated in DCM solvent using CAM‐B3LYP and ω‐B97XD functionals is provided in the Supporting Information (Figure S9c). It is found that the overall simulated electronic absorption spectrum of [7]TeH^•+^ at ω‐B97XD functional is similar to that calculated at CAM‐B3LYP functional, applying TDDFT procedure with LANL2DZ basis set for Te atoms and 6–311++G(d,p) basis set for all other atoms. Furthermore, the absorption peaks obtained in the visible region remain unaffected, but peaks in the IR region are shifted by 100–150 nm. The strong absorptions in [n]TeH radical cations (*n* = 8–10) indicate prominent NIR peaks (Table S17 and Figure S9b). Remaining frontier orbitals of [n]TeH radical cations (*n* = 1–10) are also provided in Supporting Information from Figure S10a–j. Overall, these radical cation systems showed better absorption in the infrared range than S‐ and Se‐ systems and have absorption in near to far infrared regions. This indicates that [n]TeH are potential systems for finding applications as optoelectronic devices.

## 4 Conclusions

This study introduces a reliable method to predict HOMO–LUMO energy gaps in helicenes with thiophene, selenophene, and tellurophene using 15 DFT functional‐based methodologies. Comparisons with CCSD(T) fundamental gaps show that the ωB97XD functional is able to produce accurate results. Also, a simplified process combining B3LYP geometry optimization with ωB97XD single‐point calculations offers a faster option without losing much accuracy. HOMO–LUMO gaps of [n]TeH are redshifted compared with their S‐ and Se‐analogs. Studies on dimerization show that substituted [7]TeH^•+^ radicals are more stable in DCM than similar S‐ and Se‐based systems. The HDOMO–LSOMO energy gaps of [n]TeH radical cations are redshifted compared with their S‐ and Se‐analogs. TDDFT studies with the CAM‐B3LYP functional reveal interesting infrared absorption properties of tellurophene radical cations, suggesting possible uses in optoelectronic materials. The performance of the ωB97XD functional in the calculation of excited‐state spectra is quite like the CAM‐B3LYP functional for [n]TeH systems. This research proves that ωB97XD can accurately predict HOMO–LUMO gaps and provides new understanding of [n]TeH. This work not only advances the understanding of chalcogen‐based helicenes' electronic properties but also provides a robust computational framework for predicting and optimizing the electronic characteristics of organic materials, potentially leading to the development of novel materials for various optoelectronic and electronic applications. The results are expected to help explore advanced Te‐containing organic materials for various uses in future electronics and photonics.

## Conflicts of Interest

The authors declare no conflicts of interest.

## Supporting information


**DATA S1.** Supporting Information.

## Data Availability

The data that supports the findings of this study are available in the Supporting Information of this article.
