# Supplementary material for: Accurate Prediction of HOMO–LUMO Gap Using DFT Functional and Application to Next‐Generation Organic Telluro[n]Helicenes Materials
Source: J Comput Chem. 2025 Jul 2;46(18):e70175. doi: 10.1002/jcc.70175 (PMC12223471; doi:10.1002/jcc.70175)
Supplement: Supplementary file 1 — DATA S1. Supporting Information. [file JCC-46-0-s001.doc]

# Accurate Prediction of HOMO-LUMO Gap using DFT Functional and Application to Next-generation Organic Telluro[n]helicenes Materials^†^

**Rahul Kumar,^1,2^ Rahul Kar,^3^ and Dilip K. Maity^1,2,*^**

*Correspondence to: Dilip Kumar Maity (E-mail: [dkmaity@barc.gov.in](mailto:dkmaity@barc.gov.in); [maitydk@hbni.ac.in](mailto:maitydk@hbni.ac.in) )

ORCID ID: 0000-0003-4284-3578

1 *Homi Bhabha National Institute, Training School Complex, Anushaktinagar, Mumbai-400094, India*

^2^*Bhabha Atomic Research Centre, Trombay, Mumbai-400085, India*

^3^*Department of Chemistry, Dibrugarh University, Dibrugarh-786004, Assam, India*

**^†^**In honor of Prof. Shridhar R. Gadre on the occasion of his 75th birthday.

**Table of contents**

| **1.** | **Cartesian coordinates for the most stable structures of neutral telluro[n]helicenes, n=1-10.** | **S3-S13** |
| --- | --- | --- |
| **2.** | **Most stable structures of neutral telluro[n]helicenes , [n]TeH, n=1,2,4-6,8-10 with the complete labelling of molecular properties** | **S14-S15** |
| **3.** | **Gematrical parameters of neutral and radical cations of telluro[n]helicenes, n=1-10** | **S16** |
| **4.** | **Most stable structures of telluro[n]helicenes radical cation, [n]TeH^•+^, n=1-10 with the complete labelling of molecular properties** | **S17-S18** |
| **5.** | **Cartesian coordinates for the most stable structures of radical cations of end substituted telluro[n]helicenes, n=1-10** | **S19-S29** |
| **6.** | **Spin density plot with contour cutoff = 0.003 a.u. for the most stable structures of end substituted telluro[n]helicenes radical cation, n=1-10 following ROHF formalism** | **S30-S31** |
| **7.** | **Ionization energies plots and table for thia[n]helicenes, seleno[n]helicenes, and telluro[n]helicenes in gas phase and in DCM solvent** | **S32-S33.** |
| **8.** | **Cartesian coordinates for the most stable structures π-dimer of unsubstituted telluro[7]helicene radical cation, π-dimer of substituted telluro[7]helicene radical cation, π-dimer of radical cation of substituted telluro[7]helicene with two PF_6_^-^ counter anions** | **S34-S39** |
| **9** | **Table related to statistical errors (MSE, MAE and MAX_UE)$ analysis for different density functionals against CCSD(T) level of theory** | **S40-S51** |
| **10.** | **Figures for density of states spectra for telluro[n]helicenes, [n]TeH, , n=1-10 in gas phase** | **S52-S54** |
| **11** | **HOMO-LUMO gaps at B2PLYP and ωB97XD functional. Basis set used: 6-311++G(d,p) for H, C, Si, S, Se, Br atoms and def2-TZVPP basis set for Te atoms.**  **Calculated HOMO-LUMO gaps (E_g_) at LC-ωPBE DFT functional for telluro[n]helicene, [1]TeH in gas phase with varying ω.**  **Optimized ω parameter satisfying Koopmans' theorem at LC-ωPBE and ωB97XD functionals** | **S55-56** |
| **12** | **Computational cost comparison table for thia[n]helicenes, [n]TH, seleno[n]helicenes, [n]SH and telluro[n]helicenes [n]TeH** | **S57-S58** |
| **13.** | **Simulated electronic absorptions spectra [3]TH and [7]TH in DCM, and experimentally reported UV-Visible absorption spectra of 1 [7]TH and 4 [3]TH in DCM** | **S59** |
| **14.** | **TDDFT calculation data for neutral telluro[n]helicenes (n=1-10) and their radical cations** | **S60-S56** |
| **15** | **Plots of UV-Vis spectra for telluro[n]helicenes (n=1-10) and their corresponding radical cations** | **S66-S68** |
| **16.** | **Molecular orbital plots with contour cutoff = 0.03 a.u. for the most stable structure of telluro[n]helicenes radical cation, n=1-6,8-10** | **S69-70** |

**Theoretical Methods:**

1. **DFT Functionals:** B3LYP, B3LYP-D (full optimization)**,** ωB97XD (single point energy calculations)
2. **Basis Set:** 6-311++G(d,p) for H, C, Si, Br, P, and F and lanl2dz for Te atoms
3. **Solvent Used:** Dichloromethane (DCM)
4. **Solvent Model:** Solvation model based on solute density (SMD)

**1 Cartesian coordinates for the most stable structures of neutral telluro[n]helicenes, n=1-10 calculated at B3LYP-D with 6-311++G(d,p) basis set is used for H, C, Si, Br atoms and lanl2dz basis set is for Te atoms in DCM solvent.**

## 1.1 Neutral telluro[1]helicene

C 0.000000 0.000000 0.000000

Si 0.000000 0.000000 1.881462

C 1.748442 0.000000 2.576333

C -0.938481 -1.518824 2.518371

C -0.696038 -2.848632 2.353860

Br 0.837652 -3.425682 1.313051

Te -2.662698 -1.308819 3.688526

C -2.657212 -3.403155 3.684755

Si -3.999188 -4.384470 4.595439

C -4.958288 -5.436000 3.364867

C -1.585593 -3.823896 2.957540

Br -1.259304 -5.724680 2.736110

C -0.919408 1.520877 2.505388

C -3.209912 -5.436013 5.941246

C -5.162398 -3.130882 5.384819

H -3.987765 -5.959332 6.511817

H -2.529648 -6.186354 5.527204

H -2.642354 -4.810005 6.639907

H -5.958942 -3.657171 5.925365

H -4.642796 -2.488994 6.106153

H -5.640790 -2.488986 4.635524

H -5.775819 -5.959333 3.876957

H -5.397896 -4.809984 2.579383

H -4.322254 -6.186329 2.885629

H 0.488615 0.908639 -0.374299

H 0.531874 -0.864511 -0.408571

H -1.025357 -0.016145 -0.387969

H 2.276731 0.908646 2.260514

H 1.730291 -0.016143 3.672484

H 2.324561 -0.864512 2.232959

H -0.408631 2.427846 2.158742

H -1.949040 1.558429 2.130181

H -0.951024 1.558449 3.600797

## 1.2 Neutral telluro[2]helicene

C 0.000000 0.000000 0.000000

Si 0.000000 0.000000 1.882464

C 1.790214 0.000000 2.479898

C -0.804912 -1.582700 2.547491

C -2.065590 -2.080979 2.420776

Br -3.296030 -0.995860 1.370184

Te 0.358283 -2.899308 3.712191

C -1.414564 -3.955882 3.744031

C -2.456727 -3.352549 3.040382

C -3.700289 -4.130582 3.084486

Br -5.337480 -3.555487 2.199006

Te -1.914658 -5.749400 4.634843

C -3.779783 -5.312714 3.755284

Si -5.210470 -6.533103 3.997904

C -4.551051 -7.951363 5.054076

C -0.860954 1.521857 2.579871

C -6.629596 -5.711473 4.922393

C -5.769277 -7.232963 2.342127

H -5.352129 -8.682718 5.218223

H -3.721234 -8.477666 4.567267

H -4.212080 -7.609593 6.039401

H -6.523092 -8.013828 2.505977

H -6.207326 -6.466932 1.696080

H -4.925133 -7.686502 1.809213

H -7.404086 -6.455758 5.148314

H -6.279938 -5.290491 5.872544

H -7.089723 -4.906414 4.342547

H 2.293297 0.903883 2.114582

H 1.860413 0.005213 3.574256

H 2.351619 -0.863052 2.102355

H -0.320570 2.426292 2.271955

H -1.894433 1.607801 2.232595

H -0.870869 1.492395 3.675912

H 0.561109 0.867825 -0.369958

H 0.484910 -0.904181 -0.386773

H -1.011380 0.046890 -0.413489

## Neutral telluro[3]helicene

C 0.000000 0.000000 0.000000

Si 0.000000 0.000000 1.885863

C 1.756313 0.000000 2.569809

C -0.975208 -1.475470 2.568974

C -0.711401 -2.809642 2.600463

Br 0.710774 -3.473628 1.442449

C -0.868732 1.568295 2.479276

C -1.624432 -3.768408 3.216789

C -2.859986 -3.217594 3.521975

Te -4.359733 -4.631217 3.826313

C -2.720734 -5.891716 3.571329

C -1.513531 -5.221752 3.441174

C -0.357918 -6.079590 3.688936

Br 1.306769 -5.264979 4.296945

C -0.467591 -7.431973 3.787078

Si 0.817846 -8.797138 4.067544

C -0.050071 -10.450177 3.783702

Te -2.518779 -7.943155 3.706230

C 2.222579 -8.643054 2.820387

C 1.467651 -8.765270 5.837634

Te -2.911422 -1.158837 3.360202

H 2.281960 0.904709 2.243314

H 1.747604 -0.005055 3.664613

H 2.334809 -0.863578 2.233232

H 0.491403 0.907030 -0.370562

H 0.527753 -0.862181 -0.414572

H -1.022436 -0.009787 -0.391532

H -0.321572 2.444607 2.113825

H -1.893473 1.648608 2.101054

H -0.899382 1.634801 3.571524

H 0.669581 -11.264678 3.923525

H -0.869276 -10.620358 4.490482

H -0.448788 -10.537725 2.767962

H 2.139886 -9.615518 6.000961

H 2.022079 -7.850275 6.059315

H 0.646672 -8.845344 6.557600

H 2.911393 -9.488509 2.930584

H 1.836725 -8.657362 1.795884

H 2.797399 -7.723203 2.951393

## Neutral seleno[4]helicene

C 0.000000 0.000000 0.000000

Si 0.000000 0.000000 1.885796

C 1.780963 0.000000 2.498266

C -0.937191 -1.509309 2.508123

C -0.897123 1.565611 2.443468

C -1.301181 1.970575 3.674970

Br -0.564838 1.053661 5.233664

Te -1.386271 3.084518 1.048882

C -2.079517 4.022092 2.761757

C -2.046008 3.205512 3.881372

C -2.600235 3.777113 5.113628

C -3.041249 3.115766 6.346705

C -3.548774 1.766109 6.554845

Br -4.105662 0.728365 4.997470

Te -2.539587 6.007184 3.214833

C -2.726677 5.154070 5.112706

Te -3.066198 5.961405 7.009456

C -3.157169 3.926329 7.465221

Te -3.668310 2.880503 9.179442

C -3.872551 1.296050 7.786960

Si -4.469672 -0.405989 8.346849

C -6.220798 -0.731058 7.734611

C -3.273285 -1.720269 7.726403

C -4.469891 -0.403452 10.232643

H -6.588963 -1.683821 8.136592

H -6.902348 0.061327 8.066155

H -6.264806 -0.781491 6.642530

H -4.791479 -1.383507 10.606094

H -3.470803 -0.204627 10.638076

H -5.160498 0.345641 10.639103

H -3.595910 -2.715895 8.056890

H -3.210750 -1.726007 6.633954

H -2.266340 -1.541722 8.121727

H -0.438643 -2.429994 2.178928

H -0.997785 -1.524883 3.600585

H -1.959754 -1.517611 2.112656

H 0.494707 -0.905619 -0.372169

H -1.018536 0.013046 -0.405584

H 0.542723 0.861769 -0.407548

H 2.316487 -0.870353 2.097528

H 2.306903 0.902776 2.165572

H 1.833276 -0.040123 3.590408

## Neutral telluro[5]helicene

C 0.000000 0.000000 0.000000

Si 0.000000 0.000000 1.880501

C 1.767352 0.000000 2.545932

Te 2.327990 -1.400687 4.028453

C 4.079244 -0.299247 3.982459

C 4.110410 0.647959 2.971777

C 5.371719 1.390777 2.823830

C 5.857021 2.243662 1.727858

C 6.818612 3.171056 2.092251

Te 7.205601 4.611766 0.633254

C 5.925087 3.353267 -0.424618

Te 5.302017 3.329278 -2.397782

C 4.694617 1.374913 -1.863336

Si 4.011809 0.213481 -3.186655

C 2.391052 -0.554797 -2.621691

C -0.820496 -1.586428 2.489209

C -0.943211 1.481216 2.562144

C 2.819629 0.827186 2.315521

Br 2.482956 2.457281 1.302318

Te 5.703673 -0.180856 5.282401

C 6.212016 1.317491 3.922029

Te 7.681308 2.796989 3.953621

C 5.532634 2.234858 0.292845

C 4.977370 1.180626 -0.549258

Br 4.903702 -0.643950 0.130917

C 3.687270 1.257085 -4.724856

C 5.286491 -1.110644 -3.599164

H 3.277797 0.624697 -5.522342

H 2.959567 2.052970 -4.526550

H 4.603663 1.719591 -5.111107

H 4.922231 -1.744323 -4.418044

H 6.231171 -0.654536 -3.918315

H 5.494104 -1.752420 -2.737313

H 2.011018 -1.235503 -3.394502

H 2.510794 -1.122344 -1.695220

H 1.633469 0.216265 -2.448321

H -1.032185 0.011329 -0.373485

H 0.520283 0.870886 -0.407010

H 0.490633 -0.897605 -0.390011

H -1.990468 1.445788 2.235348

H -0.930261 1.479004 3.658565

H -0.510649 2.426508 2.219969

H -1.851457 -1.636834 2.117532

H -0.293976 -2.477658 2.127552

H -0.863935 -1.635177 3.584022

## Neutral telluro[6]helicene

C 0.000000 0.000000 0.000000

Si 0.000000 0.000000 1.887330

C 1.783503 0.000000 2.493373

C -0.926658 1.517761 2.497862

C -0.894962 -1.566832 2.437115

C -1.282959 -2.002413 3.665877

Br -0.552361 -1.075071 5.218203

Te -1.309671 -3.081379 1.024453

C -1.976290 -4.061517 2.714839

C -1.971355 -3.273637 3.855226

C -2.562611 -3.882999 5.056796

C -3.008383 -3.270738 6.316484

C -3.354569 -1.875927 6.624825

C -3.727527 -0.765181 5.734994

C -4.254275 -0.762486 4.375448

Br -5.073133 -2.385229 3.668584

Te -2.532893 -6.035309 3.063878

C -2.723421 -5.255488 4.987050

Te -3.118516 -6.155545 6.824928

C -3.119929 -4.148177 7.383331

Te -3.256302 -3.205762 9.236626

C -3.326802 -1.552238 7.969780

Te -3.356308 0.496548 8.350081

C -3.661800 0.502190 6.292370

Te -4.010936 2.023647 4.941187

C -4.441024 0.388466 3.675190

Si -5.097273 0.705074 1.935295

C -4.863684 2.544973 1.585745

C -4.104052 -0.284867 0.682864

C -6.932711 0.295513 1.827429

H -5.464358 3.169691 2.258183

H -3.814361 2.846770 1.683973

H -5.179712 2.770013 0.559507

H -4.486402 -0.098902 -0.329293

H -3.050141 0.007682 0.706747

H -4.158529 -1.358625 0.878864

H 1.840031 0.040385 3.585277

H 2.306929 -0.903904 2.159692

H 2.318326 0.869286 2.089288

H -1.942690 1.539363 2.092849

H -0.999908 1.537249 3.588106

H -0.409726 2.429171 2.170254

H 0.462807 0.923192 -0.370391

H 0.572562 -0.841083 -0.409908

H -1.017159 -0.047858 -0.406048

H -7.118875 -0.771724 1.980587

H -7.499553 0.850827 2.584176

H -7.323276 0.574150 0.840119

## Neutral telluro[7]helicene

C 0.000000 0.000000 0.000000

Si 0.000000 0.000000 1.880293

C 1.769462 0.000000 2.530101

C -0.881709 -1.557722 2.476795

C -0.916486 1.469452 2.628030

C -0.814871 2.806994 2.419423

Br 0.621021 3.437003 1.263136

Te -2.132849 1.150108 4.330559

C -2.155898 3.216549 4.334807

Te -2.657616 4.607678 5.796159

C -1.909147 5.877990 4.327366

C -1.603193 5.246085 3.134560

C -1.436189 6.141831 1.979173

C -1.492848 5.857568 0.535818

C -1.983770 4.671644 -0.183723

C -2.850726 3.571482 0.263396

C -3.899704 3.543145 1.278332

Br -4.602682 5.231786 1.949194

C -1.547503 3.779603 3.224798

Te -1.676621 7.944543 4.279843

C -1.235191 7.474074 2.300929

Te -0.624207 8.658323 0.700853

C -0.959641 6.848238 -0.272334

Te -0.564275 6.231885 -2.220698

C -1.520042 4.538856 -1.481047

Te -1.774367 2.636408 -2.285550

C -2.788288 2.407239 -0.485000

Te -3.948973 0.874517 0.272808

C -4.579298 2.409214 1.587152

Si -6.061308 2.089384 2.709541

C -6.263499 0.217471 2.831177

C -7.596862 2.829393 1.904755

C -5.843720 2.777817 4.445690

H -0.836026 -1.666718 3.567148

H -1.935761 -1.559823 2.175282

H -0.406804 -2.444687 2.039298

H 0.603255 -0.846856 -0.353434

H -1.014233 -0.118374 -0.392829

H 0.421647 0.917911 -0.414755

H -7.513850 3.917574 1.815712

H -7.745320 2.417589 0.899518

H -8.490034 2.602088 2.500861

H -5.026594 2.272485 4.969085

H -5.636349 3.849901 4.443817

H -6.768719 2.606626 5.012317

H -7.095016 -0.023924 3.504940

H -6.487595 -0.238162 1.859060

H -5.359811 -0.256184 3.232341

H 2.312671 0.893164 2.204316

H 1.780740 -0.022331 3.626230

H 2.312000 -0.882060 2.166254

## Neutral telluro[8]helicene

C 0.000000 0.000000 0.000000

Si 0.000000 0.000000 1.887404

C 1.804753 0.000000 2.438311

C 2.379030 -0.136966 3.662304

Br 1.188108 -0.133262 5.204641

C -0.939157 -1.516373 2.484985

C -0.835603 1.575251 2.498535

Te 3.297836 0.551979 1.048756

C 4.480117 0.469582 2.741052

C 3.819950 -0.003117 3.863961

C 4.637932 -0.139801 5.078942

C 4.346631 -0.806949 6.355605

C 3.455394 -1.937171 6.644058

C 2.815117 -2.881624 5.720499

C 3.082645 -3.134941 4.297803

C 4.314745 -2.964950 3.512328

C 5.710178 -2.864923 3.933696

Br 6.206719 -3.382819 5.745404

Te 6.392571 1.180780 3.134550

C 5.854500 0.519994 5.030973

Te 6.736190 0.789152 6.896794

C 5.081419 -0.350847 7.435551

Te 4.447848 -1.109426 9.268843

C 3.178251 -2.142437 7.982169

Te 1.572682 -3.424413 8.318628

C 1.747103 -3.584641 6.249408

Te 0.502421 -4.420936 4.807352

C 2.021694 -3.679588 3.593540

Te 2.302504 -3.733785 1.534206

C 4.171672 -3.054613 2.136742

Te 5.920891 -2.717171 1.090411

C 6.721605 -2.678089 3.045412

Si 8.595194 -2.627317 3.263739

C 9.136758 -1.405527 4.587534

C 9.220050 -4.358592 3.670395

C 9.327980 -2.084914 1.611116

H 9.112186 -2.801301 0.808767

H 8.948058 -1.102678 1.306097

H 10.419059 -2.008277 1.696480

H 10.222818 -1.485578 4.728461

H 8.908617 -0.378143 4.286581

H 8.651687 -1.595527 5.547548

H -0.865425 1.614358 3.592020

H -0.298748 2.462408 2.142130

H -1.866462 1.629597 2.125342

H -0.551318 -2.425467 2.014598

H -0.877043 -1.639705 3.568465

H -1.997426 -1.415786 2.209768

H -1.031976 -0.032940 -0.370889

H 0.467305 0.902573 -0.412954

H 0.526269 -0.873949 -0.401773

H 8.825308 -4.712145 4.628156

H 8.917512 -5.072250 2.894862

H 10.316188 -4.364555 3.728195

## Neutral telluro[9]helicene

C 0.000000 0.000000 0.000000

Si 0.000000 0.000000 1.888104

C 1.806705 0.000000 2.434613

C 2.394379 -0.169913 3.646865

Br 1.216617 -0.161360 5.199940

C -0.946495 -1.512858 2.481128

C -0.834393 1.576326 2.498866

Te 3.277129 0.642480 1.058092

C 4.475644 0.516809 2.738180

C 3.838554 -0.032835 3.838625

C 4.682081 -0.232055 5.027332

C 4.423654 -0.988692 6.259654

C 3.437166 -2.040195 6.531888

C 2.761468 -2.947693 5.600159

C 3.057461 -3.223018 4.190582

C 4.311660 -3.065733 3.444406

C 5.696750 -2.938192 3.925024

C 6.316274 -3.302817 5.200892

Br 5.415054 -4.566617 6.379818

Te 6.383082 1.240962 3.136034

C 5.889587 0.444911 4.993840

Te 6.851038 0.533841 6.836791

C 5.212769 -0.638738 7.342033

Te 4.474339 -1.320280 9.166448

C 3.127411 -2.224931 7.864933

Te 1.489750 -3.469821 8.179144

C 1.666159 -3.614963 6.111268

Te 0.440814 -4.445158 4.649025

C 1.998452 -3.737269 3.461555

Te 2.313655 -3.727076 1.408472

C 4.182178 -3.092360 2.064299

Te 5.900236 -2.547040 1.026767

C 6.624421 -2.542252 2.973508

Te 8.526589 -2.246587 3.733322

C 7.620627 -3.040287 5.470883

Si 8.721336 -3.469967 6.941717

C 7.958671 -2.982922 8.590277

C 9.098056 -5.316755 6.911385

C 10.332566 -2.509667 6.723612

H 10.882851 -2.821990 5.827394

H 10.149324 -1.431022 6.655850

H 10.987378 -2.683529 7.586571

H 8.592889 -3.361518 9.402813

H 7.893347 -1.894368 8.686973

H 6.955642 -3.395184 8.718079

H -0.867231 1.611737 3.592506

H -0.294285 2.463680 2.147498

H -1.863941 1.634083 2.122948

H -0.580349 -2.420284 1.990349

H -0.866412 -1.654626 3.560956

H -2.008254 -1.391954 2.228335

H -1.031355 -0.054565 -0.370222

H 0.447893 0.912510 -0.412429

H 0.544992 -0.862541 -0.401183

H 8.189617 -5.911852 7.049650

H 9.548975 -5.603983 5.954206

H 9.803505 -5.576950 7.711131

## Neutral telluro[10]helicene

C 0.000000 0.000000 0.000000

Si 0.000000 0.000000 1.879089

C 1.766249 0.000000 2.537706

C -0.879517 -1.562216 2.471483

C -0.937264 1.467524 2.601773

Te -2.170433 1.142409 4.289198

C -2.230762 3.208915 4.264092

C -1.635353 3.773566 3.148217

C -1.724257 5.241009 3.045948

C -1.454594 6.143861 1.916836

C -1.372475 5.857910 0.480222

C -1.844488 4.688439 -0.259884

C -2.862723 3.709223 0.117678

C -3.956882 3.832958 1.087147

C -4.541205 5.033174 1.704231

C -4.483282 6.449188 1.300171

C -4.242126 7.063823 -0.004947

Br -4.443846 5.978233 -1.611655

C -0.860393 2.804604 2.373761

Br 0.646725 3.435123 1.309933

Te -2.755735 4.592609 5.720257

C -2.064450 5.873586 4.230559

Te -1.699017 7.920042 4.232239

C -1.220934 7.464730 2.261668

Te -0.402010 8.601190 0.719990

C -0.705988 6.803294 -0.277179

Te -0.118393 6.093835 -2.144501

C -1.245826 4.479672 -1.485270

Te -1.652334 2.614341 -2.301690

C -2.818399 2.515867 -0.579456

Te -4.012381 1.028045 0.245167

C -4.541888 2.634316 1.461100

Te -5.823215 2.792058 3.089732

C -5.325576 4.792432 2.820612

Te -5.875297 6.505872 3.869390

C -4.898512 7.366050 2.251784

Te -4.684804 9.336768 1.664052

C -4.194508 8.410971 -0.173433

Si -4.028535 9.493763 -1.707823

C -3.732325 11.259302 -1.107725

C -5.636776 9.438425 -2.689594

C -2.579374 8.988545 -2.792113

H -0.849657 -1.668996 3.562754

H -1.928800 -1.574862 2.153810

H -0.390142 -2.446154 2.043748

H 0.560232 -0.872524 -0.361492

H -1.021139 -0.064939 -0.388555

H 0.461739 0.899790 -0.411859

H -5.843889 8.428023 -3.056661

H -6.483368 9.753635 -2.068181

H -5.580328 10.112376 -3.554183

H -1.635235 9.080503 -2.246149

H -2.667545 7.958443 -3.143655

H -2.534772 9.649988 -3.667511

H -3.591228 11.923415 -1.969605

H -4.578459 11.650520 -0.529655

H -2.832050 11.324034 -0.485317

H 2.311183 0.892655 2.214035

H 1.773092 -0.022592 3.633914

H 2.309134 -0.882900 2.176219

**2** **Most stable structures of telluro[n]helicenes, [n]TeH, n=1-2,4-6,8-10**


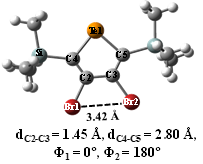

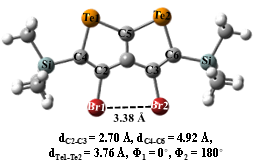


**S1a** **S1b**


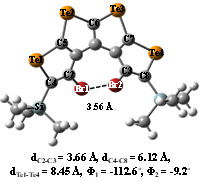
 
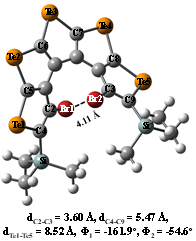


**S1c** **S1d**


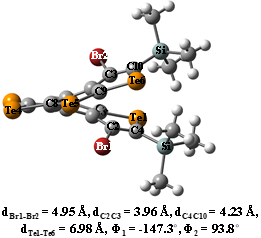
 
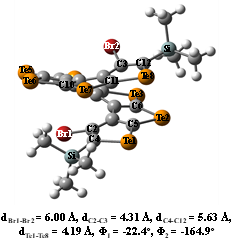


**S1e** **S1f**


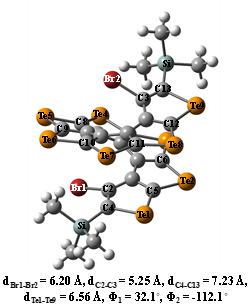

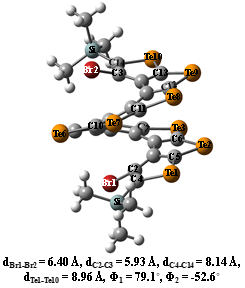


**S1g** **S1h**

**Figure S1.** Optimized structures of tellurophene-based (a-h) [n]helicenes a) [1]helicene, b) [2]helicene, c) [4]helicene, d) [5]helicene, e) [6]helicene, f) [8]helicene, g) [9]helicene, and h) [10]helicene. Φ_1_ = δ(Br1C2C3Br2) and Φ_2_ = δ(Br2C3C2C4) Computed using B3LYP-D functional in DCM solvent. Basis set used: 6-311++G(d,p) for H, C, Si, Br atoms and lanl2dz for Te atoms. Selected bond distances and dihedral angles are labelled in all figures mentioned above.

**3 Selected geometrical parameters of neutral and radicla cations of telluro[n]helicenes, n=1-10**

**Table S1**. Bond distances^#^ and dihedral angles^#^ for the most stable molecular structures of tellurophene-based [n]helicenes and their corresponding radical cations (n=1-10) calculated at B3LYP-D (without brackets) and B3LYP (in parenthesis) functionals in DCM solvent. Lanl2dz basis set is used for Te atoms and 6-311++G(d,p) basis set is considered for all other atoms.

| **Neutral tellurophene-based [n]helicenes ([n]TeH, n=1-10) in DCM solvent** | | | | | | | | | | |
| --- | --- | --- | --- | --- | --- | --- | --- | --- | --- | --- |
| **N** | 1 | 2 | 3 | 4 | 5 | 6 | 7 | 8 | 9 | 10 |
| **d_C2-C3 (Å)_** | 1.45 | 2.70 | 3.44 | 3.66 | 3.60 | 3.96 | 3.37 | 4.31 | 5.25 | 5.93 |
| **^a^d_C4-C5+n (Å)_** | 2.80 | 4.92 | 6.07 | 6.12 | 5.47 | 4.23 | 3.92 | 5.63 | 7.23 | 8.15 |
| **^b^d_Te1-Te(n) (Å)_** | - | 3.76,  *(3.75)* | 6.82,  *(6.80)* | 8.45,  *(8.48)* | 8.52,  *(8.70)* | 6.98,  *(7.28)* | 4.45,  *(5.30)* | 4.19,  *(4.76)* | 6.56,  *(6.90)* | 8.96,  *(9.26)* |
| **d_Br1-Br2 (Å)_** | 3.42,  *(3.40)* | 3.38,  *(3.36)* | 3.44,  *(3.42)* | 3.56,  *(3.55)* | 4.11,  *(4.12)* | 4.95,  *(5.11)* | 5.56,  *(5.77)* | 6.00,  *(6.17)* | 6.20,  *(6.41)* | 6.40,  *(6.70)* |
| **Φ_1_ = δ(Br1C2C3Br2) (degree)** | 0,  *(0)* | 0,  *(0)* | 72.0,  *(69.2)* | -112.6,  *(-109.8)* | -161.9,  *(-159.2)* | -147.3,  *(-145.0)* | -91.9,  *(-91.0)* | -22.4,  *(-26.6)* | 32.1,  *(27.2)* | 79.1,  *(75.3)* |
| **Φ_2 =_ δ(Br2C3C2C4) (degree)** | 180,  *(180)* | 180,  *(180)* | -78.9,  *(-82.7)* | -9.2,  *(-7.0)* | -54.6,  *(-53.5)* | 93.8,  *(95.6)* | 140.7,  *(139.9)* | -164.9,  *(-168.5)* | -112.1,  *(-117.0)* | -52.6,  *(-56.0)* |
| **Tellurophene-based [n]helicenes radical cations ([n]TeH^•+^, n=1-10) in DCM solvent** | | | | | | | | | | |
| **d_C2-C3 (Å)_** | 1.50 | 2.70 | 3.44 | 3.63 | 3.60 | 3.28 | 3.34 | 4.30 | 5.29 | 6.00 |
| **^a^d_C4-C5+n (Å)_** | 2.86 | 4.93 | 6.11 | 6.07 | 5.50 | 4.22 | 3.86 | 5.59 | 7.28 | 8.20 |
| **^b^d_Te1-Te(n) (Å)_** | - | 3.74  (3.73) | 6.82,  (6.80) | 8.35,  (8.39) | 8.50,  (8.68) | 6.95, (7.32) | 4.25,  (5.28) | 4.15,  (4.75) | 6.62,  (6.92) | 9.05,  (9.35) |
| **d_Br1-Br2 (Å)_** | 3.40,  (3.36) | 3.37,  (3.35) | 3.42,  (3.40) | 3.57,  (3.57) | 4.06,  (4.07) | 4.96, (5.10) | 5.59,  (6.01) | 6.01,  (6.17) | 6.21,  (6.42) | 6.39,  (6.66) |
| **Φ_1_ = δ(Br1C2C3Br2) (degree)** | 0,  (0) | 0,  (0) | 68.6,  (65.5) | -112.2,  (-109.3) | -159.3,  (-156.6) | -148.1,  (-146.3) | -90.5,  (-92.9) | -21.2,  (-27.9) | 33.0,  (27.5) | 79.1,  (75.2) |
| **Φ_2 =_ δ(Br2C3C2C4) (degree)** | 180,  (180) | 180,  (180) | -84.2,  (-88.2) | -6.1,  (-3.5) | -51.7,  (-50.5) | 92.4,  (93.8) | 141.5,  (137.9) | -164.2,  (-170.0) | -116.9,  (-111.7) | -51.7,  (-56.1) |

^#^**Figure 1** may be referred for identifying bond distances and dihedral angles.

^a^r_C4-C5+n_, n=0 for telluro[1]helicene, n=1 for telluro[2]helicene and so on.

^b^r_Te1-Te(n)_, n = 1 for telluro[1]helicene, n = 2 for telluro[2]helicene and so on.

**4** **Most stable structures of telluro[n]helicenes radical cation, [n]TeH^•+^, n=1-10**

**S2a S2b**

**S2c S2d**

**S2e S2f**

**S2g S2h**

**S2i S2j**

**Figure S2.** Most stable structures (S1a-S1j) of telluro[n]helicene radical cations, a) [1]helicene, b) [2]helicene, c) [3]helicene, d) [4]helicene, e) [5]helicene, f) [6]helicene, g) [7]helicene, h) [8]helicene, i) [9]helicene, and j) [10]helicene. Φ_1_ = δ(Br1C2C3Br2) and Φ_2_ = δ(Br2C3C2C4). Computed using B3LYP-D functional in DCM solvent. Basis set used: 6-311++G(d,p) for H, C, Si, Br atoms and lanl2dz for Te atoms. Selected bond distances and dihedral angles are labelled in all figures mentioned above.

# 5 Cartesian coordinates for the most stable structures of radical cations of end substituted telluro[n]helicenes, n=1-10 calculated at B3LYP-D with 6-311++G(d,p) basis set for H, C, Si, and Br atoms and lanl2dz basis set for Te atom in DCM solvent.

## 5.1 Telluro[1]helicene radical cation

C 0.000000 0.000000 0.000000

Si 0.000000 0.000000 1.875072

C 1.721724 0.000000 2.617739

C -0.911376 -1.579134 2.474504

C -0.640600 -2.892204 2.296178

Br 0.890075 -3.422575 1.288937

Te -2.666278 -1.461130 3.629320

C -2.629560 -3.564753 3.604357

Si -3.980142 -4.599616 4.492373

C -4.871435 -5.628823 3.203102

C -1.542734 -3.934740 2.889381

Br -1.153838 -5.784532 2.632811

C -1.005531 1.440900 2.536447

C -3.149519 -5.629900 5.820708

C -5.147720 -3.346351 5.261003

H -3.916770 -6.142255 6.414482

H -2.483260 -6.391367 5.404734

H -2.566479 -4.996075 6.498798

H -5.952947 -3.869536 5.790550

H -4.637714 -2.705626 5.990869

H -5.615862 -2.705036 4.504105

H -5.720581 -6.141248 3.672274

H -5.263137 -4.994391 2.399648

H -4.225896 -6.390193 2.755478

H 0.415149 0.948757 -0.362300

H 0.602397 -0.811448 -0.418856

H -1.020088 -0.092102 -0.390401

H 2.218832 0.948748 2.380022

H 1.676166 -0.092082 3.709033

H 2.344912 -0.811461 2.230521

H -0.540486 2.385734 2.230578

H -2.030467 1.438456 2.145505

H -1.052527 1.438444 3.632404

## 5.2 Telluro[2]helicene radical cation

C 0.000000 0.000000 0.000000

Si 0.000000 0.000000 1.877867

C 1.776149 0.000000 2.503208

C -0.777778 -1.625908 2.519245

C -2.041558 -2.152596 2.370875

Br -3.273774 -1.098270 1.343859

Te 0.381441 -2.919908 3.655657

C -1.349354 -4.022652 3.667160

C -2.399982 -3.440025 2.969421

C -3.619738 -4.249817 2.995988

Br -5.245862 -3.719025 2.124954

Te -1.808674 -5.830505 4.523035

C -3.659380 -5.455317 3.660610

Si -5.066054 -6.732436 3.884471

C -4.335625 -8.122428 4.923775

C -0.917773 1.467262 2.606711

C -6.492420 -5.941319 4.815060

C -5.575166 -7.408532 2.208143

H -5.101692 -8.890211 5.086365

H -3.485939 -8.608516 4.428467

H -4.007554 -7.774117 5.911007

H -6.286389 -8.231834 2.352021

H -6.055658 -6.654440 1.578663

H -4.707365 -7.804726 1.668178

H -7.237463 -6.710517 5.055080

H -6.146469 -5.502761 5.758245

H -6.990503 -5.159102 4.235560

H 2.276454 0.915187 2.164222

H 1.830410 -0.015260 3.598835

H 2.352337 -0.849691 2.116414

H -0.388575 2.391522 2.342029

H -1.943336 1.548332 2.236008

H -0.951847 1.401046 3.700360

H 0.563051 0.870150 -0.360802

H 0.488062 -0.900992 -0.389387

H -1.007961 0.052946 -0.420690

## 5.3 Telluro[3]helicene radical cation

C 0.000000 0.000000 0.000000

Si 0.000000 0.000000 1.879657

C 1.737392 0.000000 2.593631

C -0.926814 -1.538726 2.520032

C -0.568740 -2.870926 2.501969

Br 0.850753 -3.400434 1.307364

C -0.955124 1.493454 2.512993

C -1.385447 -3.875284 3.133191

C -2.636991 -3.401834 3.516782

Te -4.024394 -4.905683 3.764342

C -2.366197 -6.072051 3.393256

C -1.193541 -5.329968 3.284357

C -0.005137 -6.112097 3.508307

Br 1.619271 -5.245206 4.084692

C -0.053509 -7.489334 3.572913

Si 1.349753 -8.769817 3.740137

C 0.561590 -10.456499 3.461426

Te -2.037617 -8.115104 3.516022

C 2.635107 -8.435187 2.411912

C 2.074769 -8.689071 5.472459

Te -2.818953 -1.342534 3.364329

H 2.256698 0.920854 2.299481

H 1.707612 -0.035447 3.688791

H 2.329605 -0.849209 2.239944

H 0.429884 0.941350 -0.365245

H 0.587898 -0.825044 -0.412418

H -1.021369 -0.077915 -0.390275

H -0.461591 2.412779 2.175078

H -1.983880 1.516963 2.132349

H -0.990534 1.519678 3.608333

H 1.328649 -11.236127 3.543399

H -0.210803 -10.679397 4.208091

H 0.112796 -10.536611 2.464491

H 2.799844 -9.502403 5.603218

H 2.588408 -7.741916 5.660835

H 1.291404 -8.810969 6.229466

H 3.408758 -9.212771 2.443525

H 2.178484 -8.453857 1.415586

H 3.124905 -7.466061 2.545533

## 5.4 Telluro[4]helicene radical cation

C 0.000000 0.000000 0.000000

Si 0.000000 0.000000 1.882565

C 1.770491 0.000000 2.515621

C -0.976416 -1.472410 2.524034

C -0.885644 1.589611 2.427100

C -1.285033 2.009578 3.651068

Br -0.580567 1.115729 5.224149

Te -1.378985 3.103031 1.014465

C -2.051412 4.061492 2.685388

C -2.019065 3.261379 3.825708

C -2.619356 3.835582 5.005081

C -3.108090 3.209084 6.209083

C -3.599654 1.851652 6.437255

Br -4.121288 0.780030 4.904497

Te -2.597452 6.053128 3.078662

C -2.751445 5.256929 4.975903

Te -3.056654 6.087709 6.839720

C -3.228045 4.047266 7.315803

Te -3.718879 3.050221 9.026875

C -3.919669 1.415586 7.678904

Si -4.499530 -0.286407 8.291066

C -6.237386 -0.638128 7.665348

C -3.266369 -1.578789 7.706326

C -4.506507 -0.209804 10.172061

H -6.607781 -1.573296 8.104357

H -6.926540 0.164934 7.951874

H -6.266086 -0.739807 6.576376

H -4.795568 -1.184766 10.582651

H -3.516163 0.038245 10.572262

H -5.224583 0.530100 10.546822

H -3.552356 -2.568594 8.084081

H -3.218188 -1.632455 6.614510

H -2.261108 -1.351161 8.079591

H -0.511060 -2.407244 2.186798

H -1.019017 -1.488885 3.617276

H -2.004652 -1.449698 2.144683

H 0.465029 -0.921041 -0.371646

H -1.017526 0.045174 -0.406290

H 0.571172 0.843490 -0.407217

H 2.308954 -0.867015 2.112138

H 2.300560 0.904771 2.195690

H 1.813285 -0.052157 3.607626

## 5.5 Telluro[5]helicene radical cation

C 0.000000 0.000000 0.000000

Si 0.000000 0.000000 1.878292

C 1.782007 0.000000 2.528968

Te 2.366526 -1.411120 3.978334

C 4.121732 -0.350195 3.890098

C 4.130713 0.627208 2.911080

C 5.390979 1.363155 2.763970

C 5.898912 2.166371 1.687490

C 6.905118 3.086172 2.068104

Te 7.234829 4.567514 0.690056

C 5.942454 3.346085 -0.415156

Te 5.344152 3.382250 -2.378523

C 4.695438 1.434243 -1.911620

Si 3.955466 0.336190 -3.270163

C 2.314914 -0.378063 -2.698812

C -0.804305 -1.586537 2.500552

C -0.918246 1.486961 2.575178

C 2.834050 0.827320 2.278734

Br 2.502756 2.448411 1.268035

Te 5.794547 -0.280649 5.147257

C 6.259403 1.259472 3.876809

Te 7.772950 2.691203 3.921063

C 5.554731 2.203795 0.262123

C 4.973102 1.185882 -0.601990

Br 4.832326 -0.640790 0.032528

C 3.664138 1.445725 -4.765251

C 5.185411 -1.015060 -3.718751

H 3.253007 0.851836 -5.590688

H 2.946244 2.243857 -4.542238

H 4.591188 1.908636 -5.125325

H 4.794847 -1.616203 -4.549694

H 6.141562 -0.581828 -4.035135

H 5.378091 -1.685541 -2.875514

H 1.883893 -0.997237 -3.496024

H 2.425418 -1.000354 -1.807274

H 1.600801 0.418610 -2.466839

H -1.033386 -0.018597 -0.369415

H 0.492143 0.884825 -0.411312

H 0.514248 -0.883673 -0.391321

H -1.968533 1.460976 2.257999

H -0.895870 1.479664 3.671288

H -0.483012 2.430724 2.232251

H -1.848351 -1.626219 2.166679

H -0.297705 -2.476665 2.109202

H -0.808529 -1.647662 3.595818

## 5.6 Telluro[6]helicene radical cation

C 0.000000 0.000000 0.000000

Si 0.000000 0.000000 1.884784

C 1.777955 0.000000 2.501638

C -0.947719 1.497765 2.508550

C -0.878577 -1.587168 2.429063

C -1.254133 -2.030693 3.663354

Br -0.547115 -1.101186 5.217237

Te -1.277781 -3.091221 1.015065

C -1.929013 -4.087372 2.696090

C -1.919204 -3.308931 3.841967

C -2.494952 -3.939388 5.038451

C -2.969543 -3.357886 6.274601

C -3.354885 -2.000429 6.591163

C -3.729306 -0.881488 5.714611

C -4.245447 -0.863804 4.357631

Br -5.031324 -2.480429 3.617844

Te -2.477004 -6.062369 3.043686

C -2.661645 -5.320901 4.950491

Te -3.102419 -6.253280 6.777567

C -3.074807 -4.272756 7.358759

Te -3.170522 -3.363497 9.209486

C -3.311715 -1.691443 7.950082

Te -3.349644 0.323549 8.347716

C -3.664522 0.375364 6.293450

Te -4.024372 1.913348 4.971605

C -4.435828 0.305391 3.680862

Si -5.091942 0.648478 1.937629

C -4.879357 2.496253 1.632771

C -4.082093 -0.312125 0.677483

C -6.919998 0.213156 1.835995

H -5.483790 3.097660 2.322967

H -3.833342 2.808826 1.730479

H -5.205291 2.741499 0.614451

H -4.465714 -0.112210 -0.331386

H -3.032024 -0.007600 0.706348

H -4.126544 -1.389859 0.853113

H 1.831254 0.042734 3.593522

H 2.304244 -0.902889 2.170250

H 2.312497 0.869721 2.098541

H -1.958430 1.520525 2.091148

H -1.031644 1.504512 3.598161

H -0.431759 2.415514 2.197961

H 0.458986 0.925668 -0.368520

H 0.578377 -0.837160 -0.409909

H -1.015853 -0.051866 -0.408366

H -7.092132 -0.859390 1.965982

H -7.489699 0.745237 2.606926

H -7.319460 0.507794 0.857089

## 5.7 Telluro[7]helicene radical cation

C 0.000000 0.000000 0.000000

Si 0.000000 0.000000 1.879007

C 1.764796 0.000000 2.534382

C -0.902863 -1.537517 2.488846

C -0.902441 1.487980 2.623191

C -0.788594 2.822013 2.412419

Br 0.624413 3.443839 1.233673

Te -2.116958 1.191081 4.331922

C -2.092838 3.240345 4.365192

Te -2.598876 4.639152 5.825944

C -1.870558 5.898929 4.350225

C -1.571219 5.248401 3.140983

C -1.438528 6.128158 1.990284

C -1.534524 5.827768 0.554394

C -2.034434 4.640894 -0.121656

C -2.882578 3.561842 0.359674

C -3.895478 3.544144 1.415976

Br -4.600949 5.233868 2.063849

C -1.496931 3.799272 3.240199

Te -1.596881 7.946997 4.291727

C -1.225158 7.466210 2.295752

Te -0.680375 8.611478 0.667551

C -1.035824 6.815562 -0.285190

Te -0.712777 6.175302 -2.244531

C -1.584783 4.480904 -1.443530

Te -1.829138 2.562850 -2.189443

C -2.820325 2.376742 -0.364811

Te -3.890356 0.851454 0.489432

C -4.538315 2.405585 1.772975

Si -5.996408 2.077735 2.934327

C -6.136680 0.206547 3.107610

C -7.553238 2.757168 2.122704

C -5.766391 2.830861 4.640355

H -0.858863 -1.636644 3.580107

H -1.956263 -1.531088 2.185702

H -0.438073 -2.434408 2.061403

H 0.552149 -0.883395 -0.347051

H -1.015618 -0.055584 -0.402138

H 0.482570 0.887513 -0.414879

H -7.498892 3.843996 2.001206

H -7.702195 2.311916 1.132061

H -8.434127 2.526087 2.734987

H -4.896876 2.409052 5.152489

H -5.646212 3.915660 4.602290

H -6.657341 2.606260 5.241590

H -6.951727 -0.041768 3.798397

H -6.360525 -0.281081 2.151198

H -5.214697 -0.229666 3.509173

H 2.314422 0.886964 2.202532

H 1.773383 -0.014217 3.630525

H 2.303236 -0.887663 2.178864

## 5.8 Telluro[8]helicene radical cation

C 0.000000 0.000000 0.000000

Si 0.000000 0.000000 1.885371

C 1.812779 0.000000 2.431680

C 2.394741 -0.221246 3.640748

Br 1.219841 -0.355002 5.187845

C -0.945953 -1.512318 2.482034

C -0.812392 1.575139 2.520577

Te 3.280808 0.707043 1.084425

C 4.481869 0.491685 2.751715

C 3.830675 -0.082133 3.841202

C 4.660239 -0.321367 5.021216

C 4.378759 -1.082815 6.243499

C 3.536068 -2.244956 6.433449

C 2.924215 -3.113389 5.449438

C 3.167165 -3.207915 4.005457

C 4.381434 -2.958333 3.230142

C 5.775640 -2.872368 3.642747

Br 6.303257 -3.561541 5.385853

Te 6.400383 1.135796 3.172550

C 5.890189 0.314864 5.010037

Te 6.792259 0.394890 6.875469

C 5.135613 -0.725412 7.354503

Te 4.550695 -1.675717 9.125912

C 3.271514 -2.581603 7.778722

Te 1.682089 -3.882442 8.009644

C 1.855673 -3.867329 5.923308

Te 0.586516 -4.494376 4.431653

C 2.084533 -3.656775 3.267862

Te 2.340108 -3.504314 1.213088

C 4.216625 -2.913903 1.847664

Te 5.948308 -2.455364 0.818165

C 6.768508 -2.571237 2.763397

Si 8.649041 -2.484683 2.965035

C 9.180332 -1.499152 4.476460

C 9.318464 -4.241854 3.058661

C 9.322213 -1.628632 1.425996

H 9.103607 -2.187283 0.507597

H 8.911479 -0.618048 1.319541

H 10.413100 -1.540525 1.500333

H 10.276680 -1.437972 4.484523

H 8.784676 -0.479933 4.448744

H 8.859022 -1.964292 5.411045

H -0.819810 1.604907 3.615200

H -0.278109 2.461998 2.160289

H -1.850472 1.638035 2.170132

H -0.477333 -2.438665 2.137951

H -1.022361 -1.552912 3.570872

H -1.962782 -1.472230 2.069433

H -1.033006 -0.014372 -0.368808

H 0.481384 0.892685 -0.417985

H 0.510002 -0.884237 -0.399174

H 8.937193 -4.765868 3.941378

H 9.029650 -4.816920 2.171153

H 10.414414 -4.230958 3.114767

## 5.9 Telluro[9]helicene radical cation

C 0.000000 0.000000 0.000000

Si 0.000000 0.000000 1.886061

C 1.778515 0.000000 2.505092

C -0.843497 -1.601677 2.434447

Te -0.967485 -3.201490 1.052907

C -1.619685 -4.207054 2.731171

Te -1.872533 -6.227612 3.117001

C -2.371254 -5.430298 4.972994

Te -2.765567 -6.326414 6.791546

C -2.991411 -4.345926 7.332851

Te -3.280122 -3.384780 9.160898

C -3.400981 -1.764208 7.900835

Te -3.724808 0.271505 8.215510

C -3.936454 0.172530 6.143442

Te -4.046929 1.599476 4.666905

C -4.194895 -0.120604 3.497162

Te -4.299468 -0.446156 1.460243

C -4.640468 -2.370006 2.129462

Te -5.004807 -4.145664 1.108088

C -5.331284 -4.767304 3.058042

Te -5.942583 -6.576139 3.837385

C -6.212106 -5.390518 5.570811

Si -7.099849 -6.153895 7.056325

C -7.004610 -8.025938 6.847495

C -0.913922 1.528742 2.487166

C -1.254936 -2.044520 3.650248

C -1.802511 -3.386943 3.842556

C -2.374133 -4.039571 5.019708

C -2.907958 -3.473215 6.262460

C -3.372777 -2.122868 6.548695

C -3.877426 -1.130706 5.637641

C -4.263223 -1.284854 4.241621

C -4.704815 -2.479612 3.515135

C -5.244518 -3.747878 4.003673

C -5.843454 -4.115048 5.286420

Br -6.534724 -2.716535 6.454577

Br -0.690560 -1.010264 5.202678

C -6.288904 -5.688847 8.687165

C -8.905494 -5.619606 7.033343

H -7.521550 -8.366028 5.941611

H -5.966717 -8.375998 6.803333

H -7.486774 -8.517029 7.701690

H -6.847866 -6.154895 9.509223

H -5.255716 -6.048175 8.732864

H -6.280860 -4.608741 8.850303

H 1.824183 0.011938 3.598641

H 2.311363 -0.891145 2.152935

H 2.311200 0.883403 2.130461

H -1.930561 1.567679 2.082614

H -0.979852 1.567711 3.576860

H -0.376997 2.423089 2.144711

H 0.458931 0.925695 -0.368480

H 0.577853 -0.836671 -0.411795

H -1.017430 -0.052310 -0.404740

H -9.006794 -4.537576 7.163820

H -9.378276 -5.892448 6.082492

H -9.458731 -6.115214 7.841304

## 5.10 Telluro[10]helicene radical cation

C 0.000000 0.000000 0.000000

Si 0.000000 0.000000 1.878908

C 1.763092 0.000000 2.539890

C -0.890583 -1.552078 2.477304

C -0.935523 1.478166 2.593662

Te -2.191857 1.159504 4.264358

C -2.250502 3.219529 4.241135

C -1.629482 3.784758 3.135030

C -1.722061 5.245491 3.031784

C -1.450608 6.150560 1.916161

C -1.338822 5.867411 0.483573

C -1.807917 4.718821 -0.251046

C -2.833565 3.773334 0.113856

C -3.931252 3.931635 1.070421

C -4.511805 5.147644 1.637583

C -4.436829 6.547207 1.203340

C -4.163845 7.133200 -0.108761

Br -4.339193 6.020262 -1.698471

C -0.849412 2.814408 2.367319

Br 0.665087 3.438561 1.312203

Te -2.819478 4.603853 5.686500

C -2.089999 5.881085 4.217829

Te -1.764749 7.923741 4.219014

C -1.253072 7.478692 2.256443

Te -0.455460 8.612390 0.712170

C -0.677105 6.821918 -0.276733

Te -0.089925 6.112237 -2.154447

C -1.182242 4.490319 -1.486940

Te -1.552079 2.604665 -2.246368

C -2.774277 2.555465 -0.549885

Te -3.943459 1.110864 0.332748

C -4.509932 2.742481 1.482977

Te -5.805998 2.957352 3.090960

C -5.312883 4.938718 2.760397

Te -5.840417 6.674829 3.774321

C -4.848412 7.489330 2.136732

Te -4.603064 9.438422 1.513415

C -4.095571 8.475838 -0.299594

Si -3.884174 9.535311 -1.850039

C -3.591561 11.305758 -1.266876

C -5.465568 9.469106 -2.869941

C -2.410007 8.996980 -2.883138

H -0.862429 -1.653063 3.569093

H -1.939372 -1.564929 2.157931

H -0.403601 -2.440209 2.056078

H 0.514940 -0.901002 -0.358407

H -1.020257 -0.010769 -0.396163

H 0.512810 0.872519 -0.410502

H -5.667936 8.455214 -3.229618

H -6.326783 9.796976 -2.276059

H -5.382538 10.131048 -3.741438

H -1.482644 9.041261 -2.303460

H -2.523767 7.978834 -3.261573

H -2.308299 9.673162 -3.742126

H -3.425923 11.955570 -2.135016

H -4.450373 11.710156 -0.717421

H -2.706789 11.378457 -0.623436

H 2.314255 0.886552 2.210429

H 1.768739 -0.015449 3.636074

H 2.300894 -0.888551 2.185194

**6**  **Spin density plot with contour cutoff = 0.003 a.u. for the most stable structures of telluro[n]helicenes radical cation, [n]TeH^+●^, n=1-2,4-6,8-10 calculated at B3LYP-D functional with 6-311++G(d,p) basis set for H, C, Si, and Br atoms and lanl2dz basis set for Te atom in DCM solvent.**


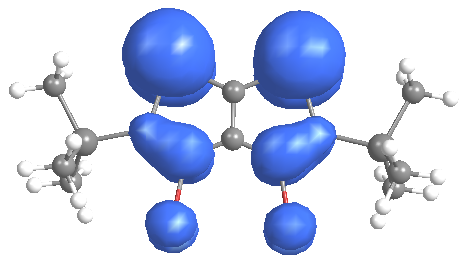


**[1]TeH^+●^ [2]TeH^+●^**

**S3a S3b**


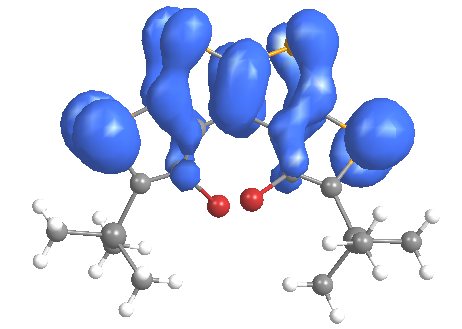

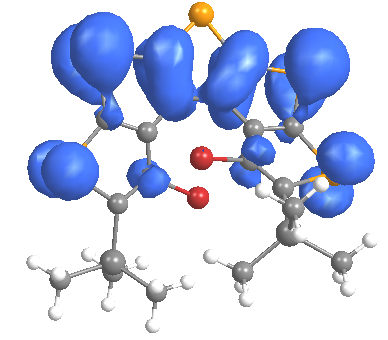


**[4]TeH^+●^ [5]TeH^+●^**

**S3c S3d**


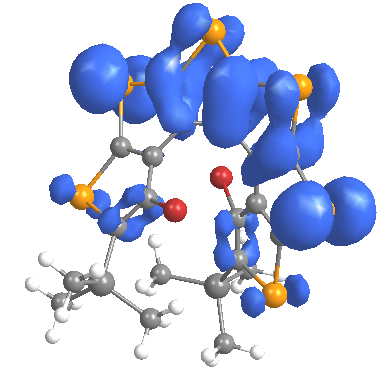

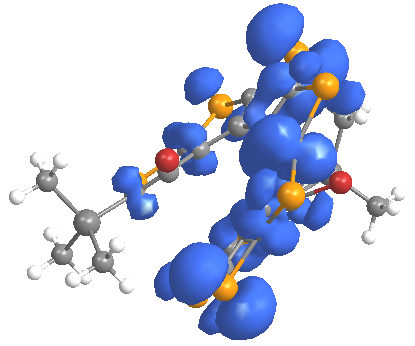


**[6]TeH^+●^ [8]TeH^+●^**

**S3e S3f**


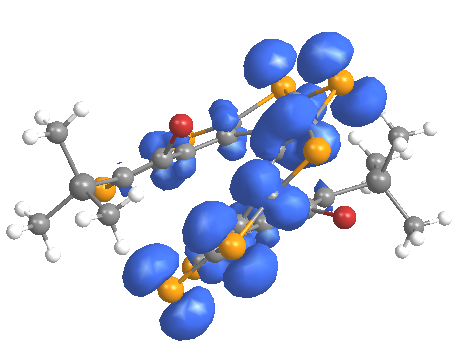

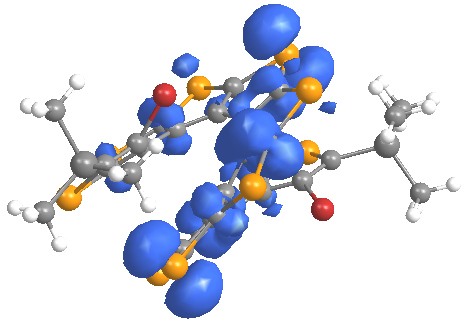


**[9]TeH^+●^ [10]TeH^+●^**

**S3i S3j**

**Figure S3.** Spin density plot for the most stable structures of telluro[n]helicenes radical cation, [n]TeH^•+^ (n=1-2, 4-6, 8-10) (S2a-S2h) at B3LYP-D functional (ROHF formalism) with 6-311++G(d,p) basis set for H, C, Si, and Br atoms and LANL2DZ basis set for Te atoms in DCM solvent.

**7.** **Ionization energies (in eV) plots and table for thia[n]helicenes, [n]TH, n=1-10 and seleno[n]helicenes, [n]SH n=1-10, and telluro[n]helicenes, [n]TeH, in DCM solvent**


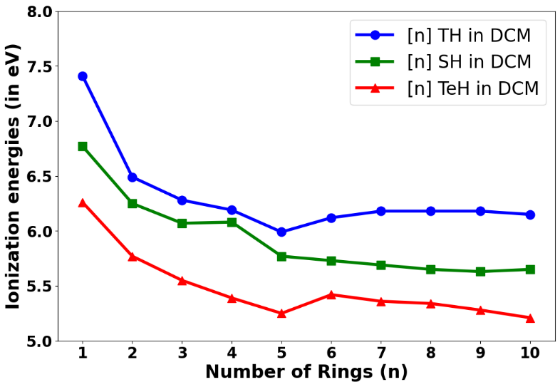


**Figure S4.** Plot of ionization energies (in eV) vs the number of rings in thia[n]helicenes, [n]TH, n=1-10 and seleno[n]helicenes, [n]SH n=1-10, and telluro[n]helicenes, [n]TeH, n=1-10 in DCM solvent: **DFT Method:** B3LYP functional (most stable geometries) and then single point energy calculations at ωB97XD functional. **Basis set used:** 6-311++G(d,p) for H, C, Si, S, Se, Br atoms and LANL2DZ basis set for Te atoms.

**Table S2**. Calculated ionization energies (in eV) vs the number of rings in thia[n]helicenes, [n]TH, n=1-10 and seleno[n]helicenes, [n]SH n=1-10 and telluro[n]helicenes, [n]the, n=1-10 in DCM solvent. Values in italics braces are for neutral systems in gas phase

| **Number of rings**  **(n)** | **E_g_ for [n] TH**  **(in eV)** | **E_g_ for [n] SH**  **(in eV)** | **E_g_ for [n] TeH**  **(in eV)** |
| --- | --- | --- | --- |
| **1** | 7.41, *(8.81)* | 6.77, *(8.49)* | 6.26, *(8.18)* |
| **2** | 6.49, *(7.82)* | 6.25, *(7.88)* | 5.77, *(7.48)* |
| **3** | 6.28, *(7.55)* | 6.07, *(7.80)* | 5.55, *(7.22)* |
| **4** | 6.19, *(7.46)* | 6.08, *(7.49)* | 5.39, *(6.96)* |
| **5** | 5.99, *(7.21)* | 5.77, *(7.33)* | 5.25, *(6.80)* |
| **6** | 6.12, *(7.34)* | 5.73, *(7.28)* | 5.42, *(6.93)* |
| **7** | 6.18, *(7.34)* | 5.69, *(7.24)* | 5.36, *(6.86)* |
| **8** | 6.18, *(7.32)* | 5.65, *(7.18)* | 5.34, *(6.79)* |
| **9** | 6.18, *(7.30)* | 5.63, *(7.09)* | 5.28, *(6.73)* |
| **10** | 6.15, *(7.26)* | 5.65, *(7.09)* | 5.21, *(6.59)* |

**DFT Method:** B3LYP functional (most stable geometries) and then single point energy calculations at ωB97XD functional. **Basis set used:** 6-311++G(d,p) for H, C, Si, S, Se, Br atoms and LANL2DZ basis set for Te atoms.

**8.** **Cartesian coordinates for the most stable structures of π-dimer systems**

**Functional used : B3LYP-D, Basis set used:6-311++G(d,p) for H, C, P, F atoms and lanl2dZ for Te atom, Solvent used: DCM (in SMD model)**

**8.1 π-dimer of unsub-[7]TeH)_2_^2+^ system**

C 0.000000 0.000000 0.000000

Te 0.000000 0.000000 2.069554

C 2.055350 0.000000 1.738279

C 2.361658 -0.029207 0.418500

C 1.274373 -0.079847 -0.532651

C 1.365528 -0.141473 -1.991354

C 2.507715 -0.440982 -2.826858

C 3.804648 -1.037304 -2.483813

C 4.197018 -1.882997 -1.350364

C 3.373052 -2.615895 -0.411758

C 1.955370 -2.886578 -0.509758

C 1.365568 -3.603295 0.468203

Te 2.734222 -4.209713 1.930679

C 4.022847 -3.184205 0.684177

Te 6.089870 -2.991311 0.620819

C 5.575042 -2.036666 -1.164888

Te 6.694714 -1.347943 -2.740841

C 4.816941 -0.773703 -3.392197

Te 4.164393 0.097611 -5.164141

C 2.373987 -0.036854 -4.167175

Te 0.444961 0.517152 -4.700413

C 0.184667 0.165710 -2.672690

Te -1.476892 0.262860 -1.453792

C -0.068638 -3.446480 -3.387560

C -1.319829 -3.169626 -2.968599

Te -2.547277 -2.525538 -4.536517

C -0.799807 -2.831224 -5.595860

Te -0.213927 -2.385709 -7.538709

C 1.607956 -3.029738 -6.742362

Te 3.528532 -2.983961 -7.461081

C 3.875294 -3.922621 -5.649354

C 2.723593 -4.183144 -4.924833

C 1.511395 -3.538099 -5.442587

C 0.237944 -3.305994 -4.793794

Te 5.626928 -4.529794 -4.709386

C 4.254389 -5.161986 -3.318757

C 2.921300 -5.044282 -3.751756

C 1.973627 -5.843053 -3.005478

C 0.613581 -6.247598 -3.363181

C -0.013130 -6.189465 -4.664637

C -1.301689 -6.595550 -4.772257

Te -2.069983 -7.266125 -2.956933

C -0.145617 -6.808492 -2.351480

Te 0.949858 -7.164310 -0.609205

C 2.429907 -6.325046 -1.774750

Te 4.473739 -6.125350 -1.491366

H 2.769291 0.030009 2.550615

H 3.391028 -0.001684 0.087867

H 1.385625 -2.516211 -1.345776

H 0.318581 -3.866054 0.519490

H -1.888908 -6.595647 -5.680881

H 0.534041 -5.846303 -5.532120

H -1.680293 -3.243515 -1.952568

H 0.689199 -3.773654 -2.695275

**8.2 π-dimer of sub-[7]TeH)_2_^2+^ system**

C 0.000000 0.000000 0.000000

Si 0.000000 0.000000 1.884512

C 1.770104 0.000000 2.528602

C -0.792051 1.618616 2.471059

Te 0.425875 3.207138 3.106781

C -1.354117 4.243831 3.010730

Te -1.825628 6.263151 2.967682

C -3.703380 5.467541 2.654183

Te -5.570316 6.379711 2.445663

C -6.146157 4.405493 2.713700

Te -8.010757 3.536375 2.818222

C -6.813126 1.912134 3.358752

Te -7.203332 -0.095905 3.633424

C -5.235974 -0.049668 4.254751

Te -3.926775 -1.545039 4.870156

C -2.760214 0.123440 5.222171

Te -0.896757 0.365698 6.066791

C -1.533346 2.388732 6.156623

Si -0.515921 3.606216 7.190210

C -1.326874 3.747159 8.883484

C -3.416084 1.332883 4.970203

C -4.681552 1.226291 4.261657

C -5.478800 2.249493 3.561783

C -5.076477 3.529091 3.000102

C -3.752038 4.080531 2.765531

C -2.440382 3.427887 2.754042

C -2.073854 2.072850 2.377851

Br -3.345906 1.032035 1.376089

C -2.766938 2.477428 5.600224

Br -3.831155 4.060503 5.991631

C -0.891433 -1.524556 2.525943

C 1.203530 2.855402 7.368364

C -0.355319 5.303154 6.397119

Br -4.565522 -2.185586 0.960489

C -6.088600 -3.340461 0.722807

C -5.914715 -4.670623 0.932598

Te -7.816423 -5.559412 1.141745

C -8.439483 -3.617366 0.933681

Te -10.271955 -2.690374 1.280721

C -9.130729 -1.050344 0.770037

Te -9.644293 0.940772 0.591142

C -7.708087 0.972597 -0.191832

Te -6.493508 2.510720 -0.810598

C -5.418168 0.901894 -1.545652

Te -3.569632 0.767731 -2.493868

C -4.107735 -1.223574 -2.478744

Te -3.054627 -2.957956 -2.923585

C -4.934766 -3.762550 -2.529161

Te -5.699022 -5.663380 -2.674532

C -7.538838 -4.628474 -2.523351

Si -9.139731 -5.563758 -2.904855

C -8.754752 -7.400476 -2.728301

C -7.415477 -2.738689 0.610096

C -7.823015 -1.364067 0.331700

C -7.162398 -0.294530 -0.396103

C -6.020553 -0.326770 -1.319175

C -5.406846 -1.455373 -2.010693

C -5.902842 -2.804227 -2.248126

C -7.269970 -3.308827 -2.368692

Br -8.710564 -2.040333 -2.685821

Si -4.367502 -5.685533 1.343194

C -3.831346 -5.231225 3.092094

C -9.618457 -5.190945 -4.687246

C -10.552635 -5.121657 -1.744585

C -4.876033 -7.499224 1.277288

C -2.940513 -5.399353 0.152978

H 1.187345 1.911104 7.925944

H 1.664017 2.671818 6.390699

H 1.854029 3.546151 7.918741

H 0.165445 5.966290 7.100506

H 0.233338 5.260342 5.475891

H -1.324713 5.748964 6.164611

H -1.019359 -0.018172 -0.399731

H 0.503028 0.891540 -0.392393

H 0.531352 -0.883401 -0.376375

H -0.763241 -1.635938 3.606721

H -1.960828 -1.499542 2.311360

H -0.468567 -2.412882 2.039041

H 2.267434 -0.937616 2.251703

H 2.363137 0.821591 2.109157

H 1.796048 0.079865 3.621784

H -2.328704 4.183032 8.811973

H -1.418023 2.761440 9.354417

H -0.723606 4.384298 9.542576

H -5.638293 -7.743316 2.027668

H -5.266940 -7.769356 0.289380

H -4.005730 -8.136202 1.476707

H -2.059680 -5.931520 0.536009

H -3.160788 -5.789424 -0.845119

H -2.683982 -4.341974 0.059294

H -9.846235 -4.129337 -4.828068

H -8.804187 -5.456968 -5.371346

H -10.505205 -5.770918 -4.972894

H -10.356866 -5.465272 -0.724325

H -10.745973 -4.047195 -1.713411

H -11.463142 -5.621934 -2.100056

H -9.663504 -7.987246 -2.910374

H -8.000827 -7.735130 -3.451432

H -8.397116 -7.643425 -1.721073

H -3.461944 -4.202195 3.136861

H -4.663092 -5.327915 3.799703

H -3.024540 -5.897184 3.423987

**8.3 π-dimer of sub-[7]TeH)_2_^2+^.(PF_6_)_2_^2-^ system**

C 0.000000 0.000000 0.000000

Si 0.000000 0.000000 1.878868

C 1.773338 0.000000 2.524976

C -0.775551 1.636996 2.441659

C -2.042695 2.115885 2.367431

Br -3.353977 0.999221 1.455270

Te 0.507363 3.227456 2.978761

C -1.250338 4.294119 2.900847

C -2.372976 3.499969 2.707036

C -3.654939 4.204844 2.738286

C -3.557114 5.588103 2.584033

Te -1.640228 6.334538 2.836032

Te -5.340581 6.519793 2.128639

C -6.005208 4.590175 2.481912

C -5.015247 3.708351 2.892275

C -5.513134 2.447006 3.480139

C -6.845845 2.159418 3.209862

Te -7.897037 3.771401 2.470526

Te -7.381748 0.184126 3.569686

C -5.430510 0.177304 4.263764

Te -4.164093 -1.370466 4.817499

C -2.923073 0.262049 5.184306

C -3.532758 1.484817 4.954520

C -4.815489 1.432710 4.252572

Te -1.056795 0.432256 6.036647

C -1.610931 2.471544 6.138080

Si -0.537756 3.656450 7.146195

C -1.296040 3.823507 8.862652

C -2.840790 2.605774 5.580987

Br -3.849414 4.228340 5.958450

C -0.884610 -1.510735 2.561887

C 1.169552 2.867810 7.283684

C -0.354574 5.349985 6.350199

F -1.523820 -2.894440 -0.317317

P -1.832460 -3.324490 -1.871884

F -0.706699 -4.521119 -1.766028

F -2.993016 -4.371794 -1.351170

F -0.676724 -2.279687 -2.397162

F -2.958635 -2.130933 -1.980387

F -2.145559 -3.758380 -3.428993

C -4.979310 0.417447 -2.259042

Si -3.774879 0.946779 -3.604429

C -2.029209 0.957070 -2.904566

C -4.102507 2.702758 -4.251065

C -5.229287 3.346558 -4.654076

Br -6.865016 2.297184 -4.806651

Te -2.430567 3.884624 -4.800481

C -3.914978 5.113112 -5.546523

C -5.200512 4.684460 -5.233840

C -6.289401 5.569932 -5.655580

C -5.925462 6.514502 -6.604121

Te -3.871272 6.715226 -6.862208

Te -7.525566 7.540607 -7.429918

C -8.529502 6.393307 -6.038532

C -7.690843 5.652801 -5.219104

C -8.338234 5.133788 -4.019865

C -9.751198 5.066869 -4.079873

Te -10.567597 6.187205 -5.606063

Te -10.600779 3.852325 -2.644128

C -8.606118 3.832963 -2.036116

Te -7.576598 2.597702 -0.783284

C -6.000848 3.804333 -1.386138

C -6.399299 4.795818 -2.266287

C -7.766355 4.659364 -2.790438

Te -4.042601 3.971704 -0.817880

C -4.163506 5.739573 -1.911491

Si -2.694543 6.937902 -1.828463

C -3.073237 8.276402 -0.556387

C -5.422755 5.850252 -2.434689

Br -5.987707 7.568895 -3.121990

C -3.879260 -0.243113 -5.057774

C -1.211562 5.929349 -1.245940

C -2.290128 7.722682 -3.485378

F -13.196385 4.588342 -4.377786

P -14.276978 5.580315 -3.610091

F -15.515551 4.744964 -4.288661

F -14.247495 6.595252 -4.901307

F -13.023024 6.405363 -2.936029

F -15.343856 6.564067 -2.845604

F -14.286671 4.554988 -2.324181

H 1.142286 1.909945 7.817770

H 1.611648 2.697828 6.295166

H 1.840765 3.532805 7.841055

H 0.289609 5.968316 6.989394

H 0.113129 5.277268 5.364416

H -1.312779 5.861108 6.235169

H -1.016699 -0.042363 -0.394347

H 0.479593 0.907400 -0.386085

H 0.542283 -0.868038 -0.389549

H -0.747561 -1.587205 3.645190

H -1.954556 -1.504441 2.346157

H -0.452321 -2.406007 2.098956

H 2.257822 -0.949582 2.265929

H 2.375539 0.804080 2.084317

H 1.805629 0.103073 3.616168

H -2.299291 4.259989 8.813679

H -1.375402 2.844706 9.350046

H -0.674103 4.469238 9.495705

H -1.359019 5.500044 -0.247988

H -0.979599 5.119021 -1.946251

H -0.330897 6.580835 -1.184789

H -1.447452 8.412057 -3.343111

H -1.993427 6.970712 -4.221266

H -3.128507 8.290181 -3.895237

H -4.887380 -0.268049 -5.485560

H -3.179692 0.046280 -5.850695

H -3.620329 -1.256047 -4.726219

H -4.849472 1.001854 -1.344687

H -6.020473 0.494165 -2.581121

H -4.772402 -0.629901 -2.015817

H -1.798474 -0.029854 -2.495966

H -1.277083 1.175307 -3.672906

H -1.924910 1.692055 -2.099317

H -4.050902 8.739187 -0.724321

H -3.051573 7.861898 0.456635

H -2.309969 9.063096 -0.612154

**9** **Statistical Errors (MSE, MAE and MAX_UE)^$^ analysis for different density functionals against CCSD(T) level of theory**

**Table S3**: HOMO energies from different density functionals for Thia[n]helicenes, n=1-5

| n | Units | Set 1 | | | | | | | | Set 2 | | | | |
| --- | --- | --- | --- | --- | --- | --- | --- | --- | --- | --- | --- | --- | --- | --- |
|  |  | BLYP | B3LYP | B3LYP-D | B3LYP-D3 | PBE0 | LC-BLYP | MN15 | ωB97XD | M06 | CAM-B3LYP | ωB97XD | LC-ωPBE | LC-BLYP |
| 1 | a.u | -0.20862 | -0.24580 | -0.24583 | -0.24577 | -0.28767 | -0.35691 | -0.27814 | -0.32088 | -0.25579 | -0.30025 | -0.32030 | -0.35037 | -0.35402 |
|  | eV | -5.67676 | -6.68846 | -6.68928 | -6.68765 | -7.82779 | -9.71188 | -7.56847 | -8.73147 | -6.96030 | -8.17010 | -8.71568 | -9.53392 | -9.63324 |
| 2 | a.u | -0.19746 | -0.23263 | -0.23269 | -0.23269 | -0.27179 | -0.33287 | -0.26204 | -0.30286 | -0.23997 | -0.28344 | -0.30346 | -0.33049 | -0.33390 |
|  | eV | -5.37308 | -6.33009 | -6.33173 | -6.33173 | -7.39568 | -9.05773 | -7.13037 | -8.24112 | -6.52982 | -7.71269 | -8.25745 | -8.99296 | -9.08575 |
| 3 | a.u | -0.19048 | -0.22457 | -0.22457 | -0.22448 | -0.26261 | -0.32397 | -0.25360 | -0.29403 | -0.23178 | -0.27445 | -0.29445 | -0.32098 | -0.32323 |
|  | eV | -5.18315 | -6.11077 | -6.11077 | -6.10833 | -7.14588 | -8.81555 | -6.90071 | -8.00085 | -6.30697 | -7.46806 | -8.01228 | -8.73419 | -8.79541 |
| 4 | a.u | -0.18571 | -0.21901 | -0.21904 | -0.21893 | -0.25618 | -0.31651 | -0.24790 | -0.28782 | -0.22625 | -0.26788 | -0.28782 | -0.31377 | -0.31613 |
|  | eV | -5.05335 | -5.95948 | -5.96030 | -5.95730 | -6.97091 | -8.61255 | -6.74561 | -7.83187 | -6.15649 | -7.28928 | -7.83187 | -8.53800 | -8.60221 |
| 5 | a.u | -0.18331 | -0.21629 | -0.21638 | -0.21628 | -0.25306 | -0.31293 | -0.24477 | -0.28442 | -0.22306 | -0.26486 | -0.28471 | -0.31074 | -0.31319 |
|  | eV | -4.98805 | -5.88547 | -5.88792 | -5.88520 | -6.88602 | -8.51514 | -6.66044 | -7.73935 | -6.06969 | -7.20711 | -7.74724 | -8.45555 | -8.52221 |

**Table S4**: LUMO energies from different density functionals for Thia[n]helicenes, n=1-5

| n | Units | Set 1 | | | | | | | | | Set 2 | | | | |
| --- | --- | --- | --- | --- | --- | --- | --- | --- | --- | --- | --- | --- | --- | --- | --- |
|  |  | BLYP | B3LYP | B3LYP-D | B3LYP-D3 | PBE0 | LC-BLYP | MN15 | ωB97XD | M06 | | CAM-B3LYP | ωB97XD | LC-ωPBE | LC-BLYP |
| 1 | a.u | -0.04819 | -0.02568 | -0.02585 | -0.02578 | 0.01916 | 0.03413 | 0.00558 | 0.04375 | -0.03496 | | 0.01803 | 0.04375 | 0.04003 | 0.03416 |
|  | eV | -1.31130 | -0.69878 | -0.70340 | -0.70150 | 0.52136 | 0.92871 | 0.15184 | 1.19048 | -0.95130 | | 0.49061 | 1.19048 | 1.08926 | 0.92953 |
| 2 | a.u | -0.05178 | -0.03088 | -0.03106 | -0.03106 | 0.01185 | 0.03080 | -0.00050 | 0.04009 | -0.03587 | | 0.01312 | 0.03805 | 0.03599 | 0.03074 |
|  | eV | -1.40899 | -0.84028 | -0.84517 | -0.84517 | 0.32245 | 0.83810 | -0.01361 | 1.09089 | -0.97606 | | 0.35701 | 1.03538 | 0.97932 | 0.83647 |
| 3 | a.u | -0.05605 | -0.03613 | -0.03631 | -0.03636 | 0.00508 | 0.02877 | -0.00638 | 0.03413 | -0.03811 | | 0.00696 | 0.03216 | 0.03414 | 0.02869 |
|  | eV | -1.52518 | -0.98313 | -0.98803 | -0.98939 | 0.13823 | 0.78286 | -0.17361 | 0.92871 | -1.03701 | | 0.18939 | 0.87511 | 0.92898 | 0.78068 |
| 4 | a.u | -0.05925 | -0.03997 | -0.04020 | -0.04026 | 0.00027 | 0.02757 | -0.01078 | 0.02977 | -0.04007 | | 0.00251 | 0.02783 | 0.03323 | 0.02751 |
|  | eV | -1.61225 | -1.08762 | -1.09388 | -1.09551 | 0.00735 | 0.75021 | -0.29333 | 0.81007 | -1.09034 | | 0.06830 | 0.75728 | 0.90422 | 0.74857 |
| 5 | a.u | -0.06177 | -0.04301 | -0.04328 | -0.04332 | -0.00350 | 0.02684 | -0.01424 | 0.02634 | -0.04058 | | -0.00094 | 0.02443 | 0.03244 | 0.02679 |
|  | eV | -1.68082 | -1.17035 | -1.17769 | -1.17878 | -0.09524 | 0.73034 | -0.38748 | 0.71674 | -1.10422 | | -0.02558 | 0.66476 | 0.88272 | 0.72898 |

**Table S5**: CCSD(T) energies of anions, cations and neutral systems (in a.u) and CCSD(T) IP and CCSD(T) EA (in eV) for Thia[n]helicenes, n=1-5 systems

| number of rings (n) | CCSD_Ani (a.u) | CCSD_Cat (a.u) | CCSD_Neu (a.u) | CCSD_IP (eV) | CCSD_EA (eV) | IP-EA (eV) |
| --- | --- | --- | --- | --- | --- | --- |
| 1 | -552.077445 | -551.784484 | -552.109713 | 8.8498036 | -0.87805271 | 9.72785631 |
| 2 | -1025.78947 | -1025.51532 | -1025.81736 | 8.21886214 | -0.75895561 | 8.97781775 |
| 3 | -1499.50171 | -1499.24082 | -1499.52681 | 7.78216369 | -0.68309134 | 8.46525502 |
| 4 | -1973.21342 | -1972.95906 | -1973.23657 | 7.55144978 | -0.63015778 | 8.18160756 |
| 5 | -2446.92447 | -2446.67445 | -2446.94638 | 7.39944641 | -0.59598076 | 7.99542718 |

**Table S6**: HOMO energies from different density functionals for Seleno[n]helicenes, n=1-4

| n | Units | Set 1 | | | | | | | | Set 2 | | | | |
| --- | --- | --- | --- | --- | --- | --- | --- | --- | --- | --- | --- | --- | --- | --- |
|  |  | BLYP | B3LYP | B3LYP-D | B3LYP-D3 | PBE0 | LC-BLYP | MN15 | ωB97XD | M06 | CAM-B3LYP | ωB97XD | LC-ωPBE | LC-BLYP |
| 1 | a.u | -0.20781 | -0.24518 | -0.24518 | -0.24511 | -0.28724 | -0.34846 | -0.27647 | -0.31847 | -0.25372 | -0.29827 | -0.31831 | -0.34478 | -0.34855 |
|  | eV | -5.65472 | -6.67159 | -6.67159 | -6.66969 | -7.81609 | -9.48195 | -7.52303 | -8.66589 | -6.90397 | -8.11622 | -8.66153 | -9.38181 | -9.48439 |
| 2 | a.u | -0.19098 | -0.22439 | -0.22449 | -0.22446 | -0.26222 | -0.32227 | -0.25189 | -0.29279 | -0.23148 | -0.27415 | -0.29357 | -0.31990 | -0.32392 |
|  | eV | -5.19676 | -6.10588 | -6.10860 | -6.10778 | -7.13527 | -8.76929 | -6.85418 | -7.96711 | -6.29880 | -7.45990 | -7.98833 | -8.70480 | -8.81419 |
| 3 | a.u | -0.18533 | -0.21852 | -0.21873 | -0.21861 | -0.25571 | -0.31620 | -0.24602 | -0.28629 | -0.22589 | -0.26788 | -0.28711 | -0.31362 | -0.31657 |
|  | eV | -5.04301 | -5.94615 | -5.95186 | -5.94860 | -6.95812 | -8.60412 | -6.69445 | -7.79024 | -6.14669 | -7.28928 | -7.81255 | -8.53391 | -8.61419 |
| 4 | a.u | -0.18054 | -0.21332 | -0.21344 | -0.21329 | -0.24975 | -0.30913 | -0.24092 | -0.28044 | -0.22072 | -0.26151 | -0.28083 | -0.30630 | -0.30954 |
|  | eV | -4.91267 | -5.80465 | -5.80792 | -5.80383 | -6.79595 | -8.41174 | -6.55567 | -7.63105 | -6.00601 | -7.11595 | -7.64167 | -8.33473 | -8.42289 |

**Table S7**: LUMO energies from different density functionals for Seleno[n]helicenes, n=1-4

| n | Units | Set 1 | | | | | | | | Set 2 | | | | |
| --- | --- | --- | --- | --- | --- | --- | --- | --- | --- | --- | --- | --- | --- | --- |
|  |  | BLYP | B3LYP | B3LYP-D | B3LYP-D3 | PBE0 | LC-BLYP | MN15 | ωB97XD | M06 | CAM-B3LYP | ωB97XD | LC-ωPBE | LC-BLYP |
| 1 | a.u | -0.05388 | -0.03237 | -0.03263 | -0.03248 | 0.01099 | 0.03359 | -0.00219 | 0.03880 | -0.03533 | 0.01248 | 0.03689 | 0.03963 | 0.03362 |
|  | eV | -1.46613 | -0.88082 | -0.88789 | -0.88381 | 0.29905 | 0.91402 | -0.05959 | 1.05579 | -0.96136 | 0.33959 | 1.00381 | 1.07837 | 0.91483 |
| 2 | a.u | -0.05869 | -0.03931 | -0.03959 | -0.03953 | 0.00068 | 0.03120 | -0.01080 | 0.02919 | -0.03592 | 0.00301 | 0.02719 | 0.03635 | 0.03115 |
|  | eV | -1.59701 | -1.06966 | -1.07728 | -1.07565 | 0.01850 | 0.84898 | -0.29388 | 0.79429 | -0.97742 | 0.08191 | 0.73987 | 0.98912 | 0.84762 |
| 3 | a.u | -0.06264 | -0.04440 | -0.04469 | -0.04466 | -0.00605 | 0.02953 | -0.01686 | 0.02307 | -0.04080 | -0.00316 | 0.02108 | 0.02934 | 0.02943 |
|  | eV | -1.70450 | -1.20817 | -1.21606 | -1.21524 | -0.16463 | 0.80354 | -0.45878 | 0.62776 | -1.11021 | -0.08599 | 0.57361 | 0.79837 | 0.80082 |
| 4 | a.u | -0.06554 | -0.04801 | -0.04841 | -0.04833 | -0.01078 | 0.02820 | -0.02125 | 0.01877 | -0.04439 | -0.00743 | 0.01683 | 0.02460 | 0.02481 |
|  | eV | -1.78341 | -1.30640 | -1.31728 | -1.31511 | -0.29333 | 0.76735 | -0.57823 | 0.51075 | -1.20790 | -0.20218 | 0.45796 | 0.66939 | 0.67510 |

**Table S8**: CCSD(T) energies of anions, cations and neutral systems (in a.u) and CCSD(T) IP and CCSD(T) EA (in eV) for Seleno[n]helicenes, n=1-4

| number of rings (n) | CCSD_Ani (au) | CCSD_Cat (au) | CCSD_Neu (au) | CCSD_IP (eV) | CCSD_EA (eV) | IP-EA (eV) |
| --- | --- | --- | --- | --- | --- | --- |
| 1 | -2554.32179 | -2554.02864 | -2554.35275 | 8.81938 | -0.84257 | 9.66195 |
| 2 | -5030.28087 | -5030.01423 | -5030.30798 | 7.99314 | -0.73770 | 8.73084 |
| 3 | -7506.24041 | -7505.98387 | -7506.26482 | 7.64484 | -0.66411 | 8.30896 |
| 4 | -9982.20000 | -9981.94816 | -9982.22141 | 7.43555 | -0.58255 | 8.01811 |

**Table S9**: HOMO energies from different density functionals for Telluro[n]helicenes, n=1-4

| n | Units | Set 1 | | | | | | | | Set 2 | | | | |
| --- | --- | --- | --- | --- | --- | --- | --- | --- | --- | --- | --- | --- | --- | --- |
|  |  | BLYP | B3LYP | B3LYP-D | B3LYP-D3 | PBE0 | LC-BLYP | MN15 | ωB97XD | M06 | CAM-B3LYP | ωB97XD | LC-ωPBE | LC-BLYP |
| 1 | a.u | -0.19548 | -0.22822 | - 0.22812 | - 0.22819 | -0.2668 | -0.32350 | -0.25500 | -0.29749 | -0.23225 | -0.27703 | -0.2974 | -0.32244 | -0.32380 |
|  | eV | -5.31921 | -6.21009 | -6.20737 | -6.209278 | -7.26017 | -8.80276 | -6.93881 | -8.095 | -6.31975 | -7.53826 | -8.09119 | -8.77391 | -8.81092 |
| 2 | a.u | -0.17694 | -0.20889 | - 0.20903 | - 0.20897 | -0.246 | -0.30244 | -0.23394 | -0.27582 | -0.21301 | -0.25691 | -0.2766 | -0.30269 | -0.30414 |
|  | eV | -4.81471 | -5.68411 | -5.68792 | -5.68628 | -6.69309 | -8.22969 | -6.36574 | -7.505338 | -5.79622 | -6.99078 | -7.5252 | -8.2365 | -8.27595 |
| 3 | a.u | -0.17234 | -0.20448 | - 0.20471 | - 0.20459 | -0.2415 | -0.29854 | -0.22968 | -0.27123 | -0.20874 | -0.25248 | -0.272 | -0.29868 | -0.30006 |
|  | eV | -4.68954 | -5.56411 | -5.57036 | -5.567098 | -6.57146 | -8.12357 | -6.24982 | -7.38044 | -5.68002 | -6.87023 | -7.40248 | -8.12738 | -8.16493 |
| 4 | a.u | -0.16909 | -0.20113 | - 0.20117 | - 0.20116 | -0.2375 | -0.29436 | -0.22669 | -0.26770 | -0.20562 | -0.24843 | -0.2682 | -0.29415 | -0.29505 |
|  | eV | -4.60111 | -5.47295 | -5.47404 | -5.473764 | -6.46261 | -8.00983 | -6.16846 | -7.284385 | -5.59513 | -6.76003 | -7.2969 | -8.00412 | -8.02861 |

**Table S10**: LUMO energies from different density functionals for Telluro[n]helicenes, n=1-4

| n | Units | Set 1 | | | | | | | | Set 2 | | | | |
| --- | --- | --- | --- | --- | --- | --- | --- | --- | --- | --- | --- | --- | --- | --- |
|  |  | BLYP | B3LYP | B3LYP-D | B3LYP-D3 | PBE0 | LC-BLYP | MN15 | ωB97XD | M06 | CAM-B3LYP | ωB97XD | LC-ωPBE | LC-BLYP |
| 1 | a.u | -0.06229 | -0.04246 | - 0.04289 | - 0.04272 | -0.0013 | 0.03267 | -0.00948 | 0.02735 | -0.04061 | -0.00082 | 0.02518 | 0.03395 | 0.03014 |
|  | eV | -1.69497 | -1.15538 | -1.16708 | -1.162453 | -0.03619 | 0.88898 | -0.25796 | 0.744221 | -1.10504 | -0.02231 | 0.68517 | 0.92381 | 0.82014 |
| 2 | a.u | -0.07596 | -0.05691 | - 0.05718 | - 0.05719 | -0.0162 | 0.02603 | -0.02668 | 0.01380 | -0.04997 | -0.01443 | 0.01131 | 0.02426 | 0.02146 |
|  | eV | -2.06695 | -1.54858 | -1.55592 | -1.556197 | -0.44027 | 0.70830 | -0.72599 | 0.375512 | -1.35973 | -0.39265 | 0.30775 | 0.66013 | 0.58394 |
| 3 | a.u | -0.07990 | -0.06138 | - 0.06133 | - 0.0616 | -0.0214 | 0.02134 | -0.03258 | 0.00883 | -0.05298 | -0.01922 | 0.00633 | 0.01966 | 0.01650 |
|  | eV | -2.17416 | -1.67021 | -1.66885 | -1.676197 | -0.58123 | 0.58068 | -0.88653 | 0.240273 | -1.44164 | -0.523 | 0.17224 | 0.53496 | 0.44898 |
| 4 | a.u | -0.08536 | -0.06742 | - 0.06755 | - 0.06766 | -0.0281 | 0.01540 | -0.03991 | 0.00218 | -0.05874 | -0.02550 | -0.0003 | 0.01343 | 0.01040 |
|  | eV | -2.32273 | -1.83457 | -1.8381 | -1.841096 | -0.76408 | 0.41904 | -1.08599 | 0.05932 | -1.59837 | -0.69388 | -0.0068 | 0.36544 | 0.28299 |

**Table S11**: CCSD(T) energies of anions, cations and neutral systems (in a.u) and CCSD(T) IP and CCSD(T) EA (in eV) for Telluro[n]helicenes, n=1-4

| number of rings (n) | CCSD_Ani (au) | CCSD_Cat (au) | CCSD_Neu (au) | CCSD_IP (eV) | CCSD_EA (eV) | IP-EA (eV) |
| --- | --- | --- | --- | --- | --- | --- |
| 1 | -6737.15828 | -6736.89897 | -6737.19244 | 7.98565 | -0.92948 | 8.91513 |
| 2 | -13395.97128 | -13395.73596 | -13396.00174 | 7.23220 | -0.82892 | 8.06113 |
| 3 | -20054.79259 | -20054.55770 | -20054.81367 | 6.96531 | -0.57349 | 7.53879 |
| 4 | -26713.60010 | -26713.37059 | -26713.62128 | 6.82151 | -0.57631 | 7.39782 |

**Table S12. Calculated HOMO-LUMO gaps (E_g_), for thia[n]helicenes, [n]TH, n=1-4 and seleno[n]helicenes, [n]SH, , n=1-4 and telluro[n]helicenes, [n]TeH, , n=1-4in gas phase.**

| [n]TH | HOMO | LUMO | Energy gap (eV) | [n]SH | HOMO | LUMO | Energy gap (eV) | [n]TeH | HOMO | LUMO | Energy gap (eV) |
| --- | --- | --- | --- | --- | --- | --- | --- | --- | --- | --- | --- |
| 1 | -0.23610 | -0.05402 | 4.95, (5.99) | 1 | -0.234980 | -0.061430 | 4.72, (5.78) | 1 | -0.22382 | -0.06889 | 4.22, (5.12) |
| 2 | -0.21965 | -0.04816 | 4.67, (5.49) | 2 | -0.214610 | -0.057630 | 4.27, (5.03) | 2 | -0.20585 | -0.07788 | 3.48, (4.21) |
| 3 | -0.21642 | -0.05024 | 4.52, (5.13) | 3 | -0.209610 | -0.059140 | 4.09, (4.73) | 3 | -0.19996 | -0.08327 | 3.18, (3.97) |
| 4 | -0.21251 | -0.05161 | 4.38, (4.87) | 4 | -0.206360 | -0.060690 | 3.96, (4.49) | 4 | -0.19771 | -0.08077 | 3.18, (4.49) |

**Method:** HSE06 functional, Basis set used: 6-311++G(d,p) for H, C, Si, S, Se, Br atoms and LANL2DZ basis set for Te atom. Values in the braces are corresponding values at B3LYP functional with same basis set.

**10 Figures for density of states spectra of telluro[n]helicenes, [n]TeH, , n=1-10 in gas phase**


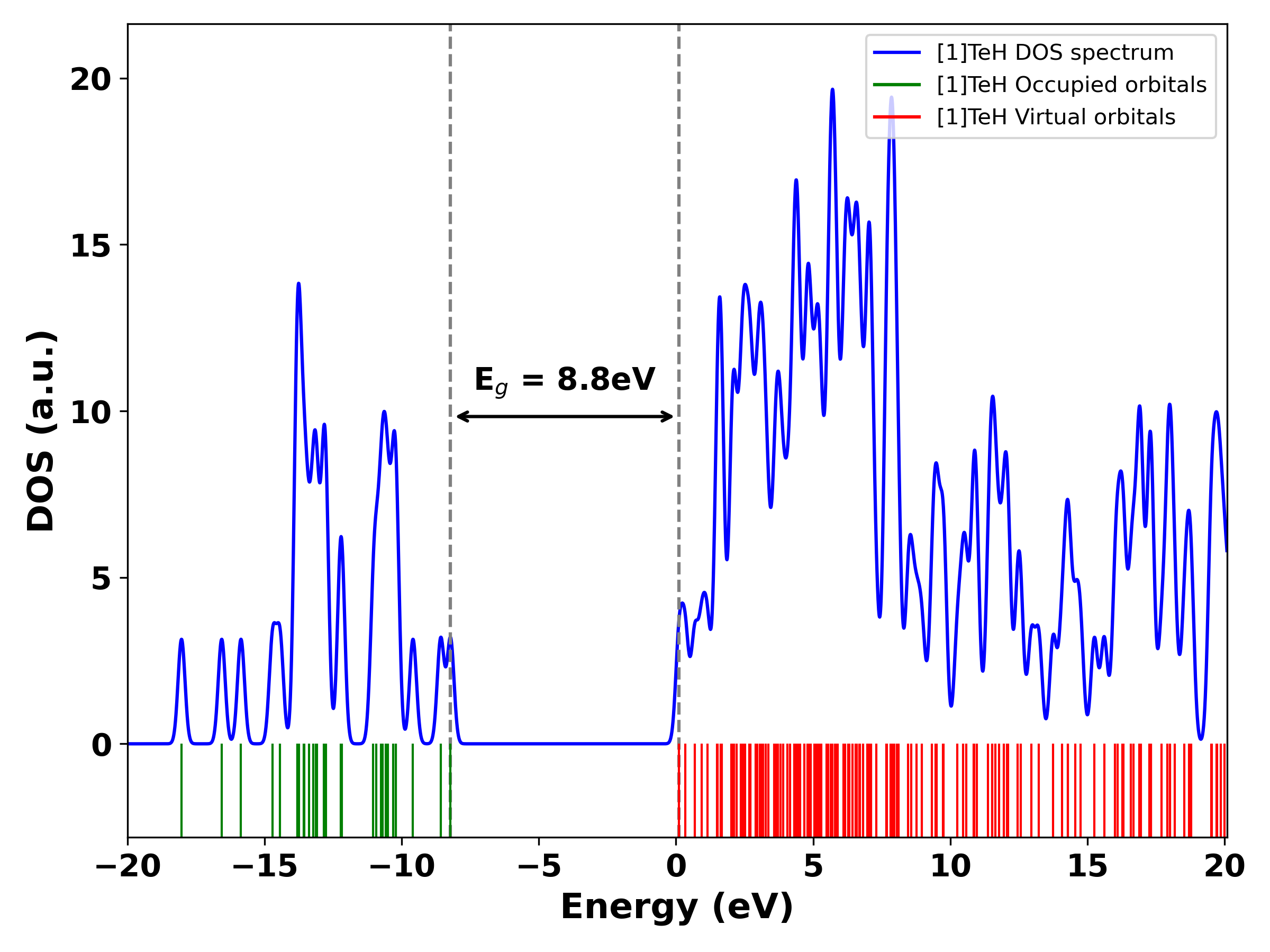

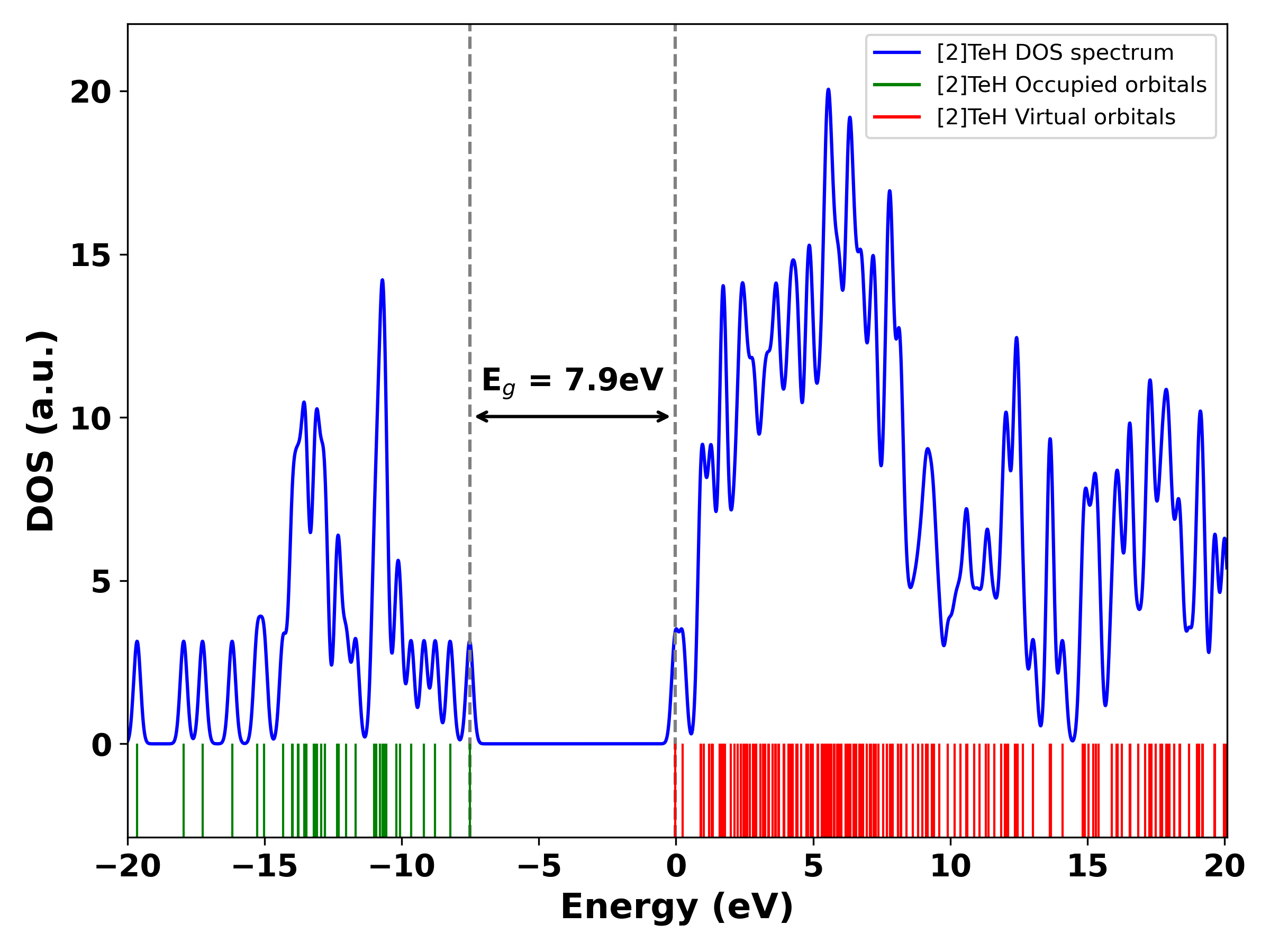


**S5 ([1]TeH) S5b ([2]TeH)**


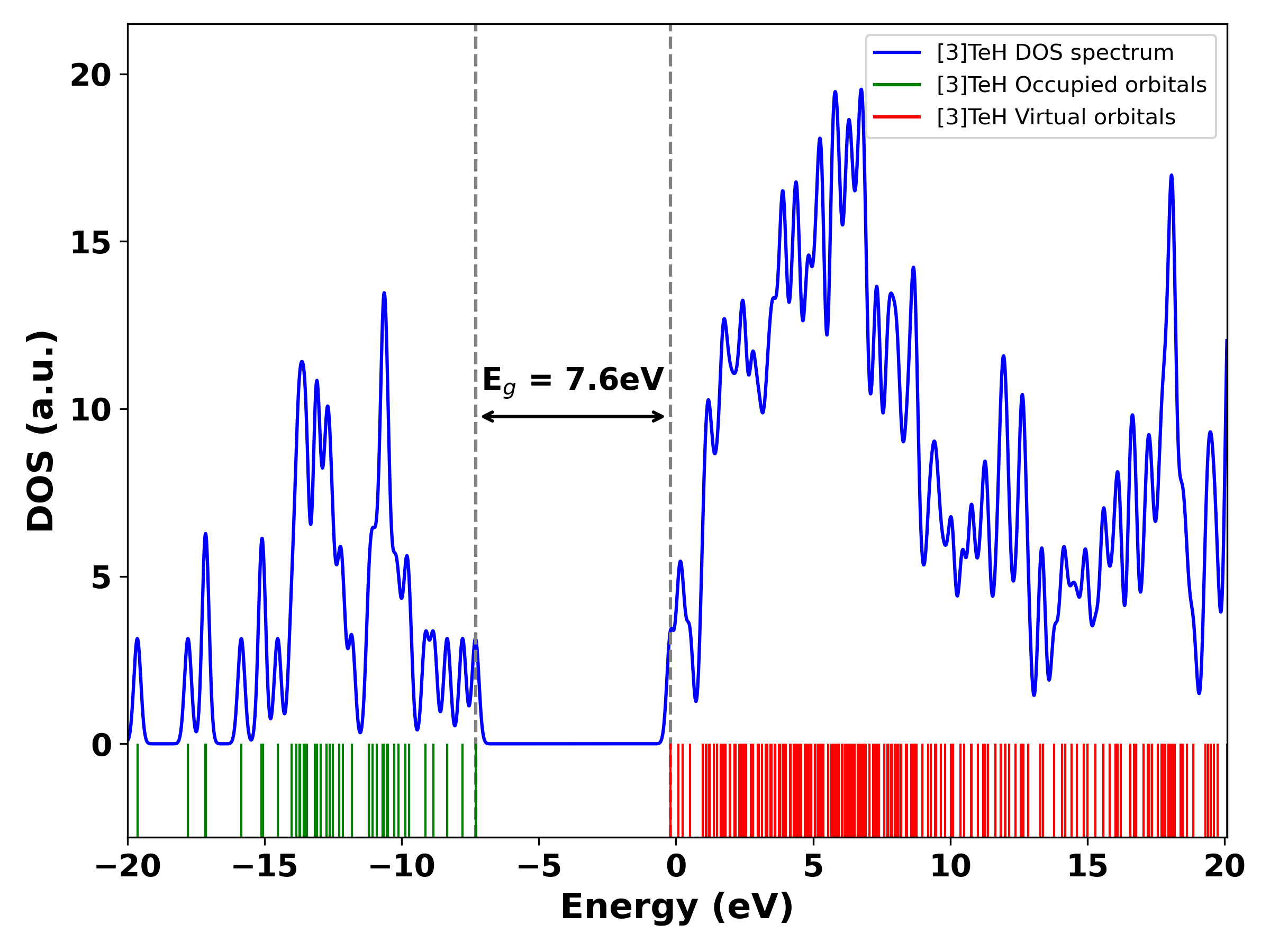

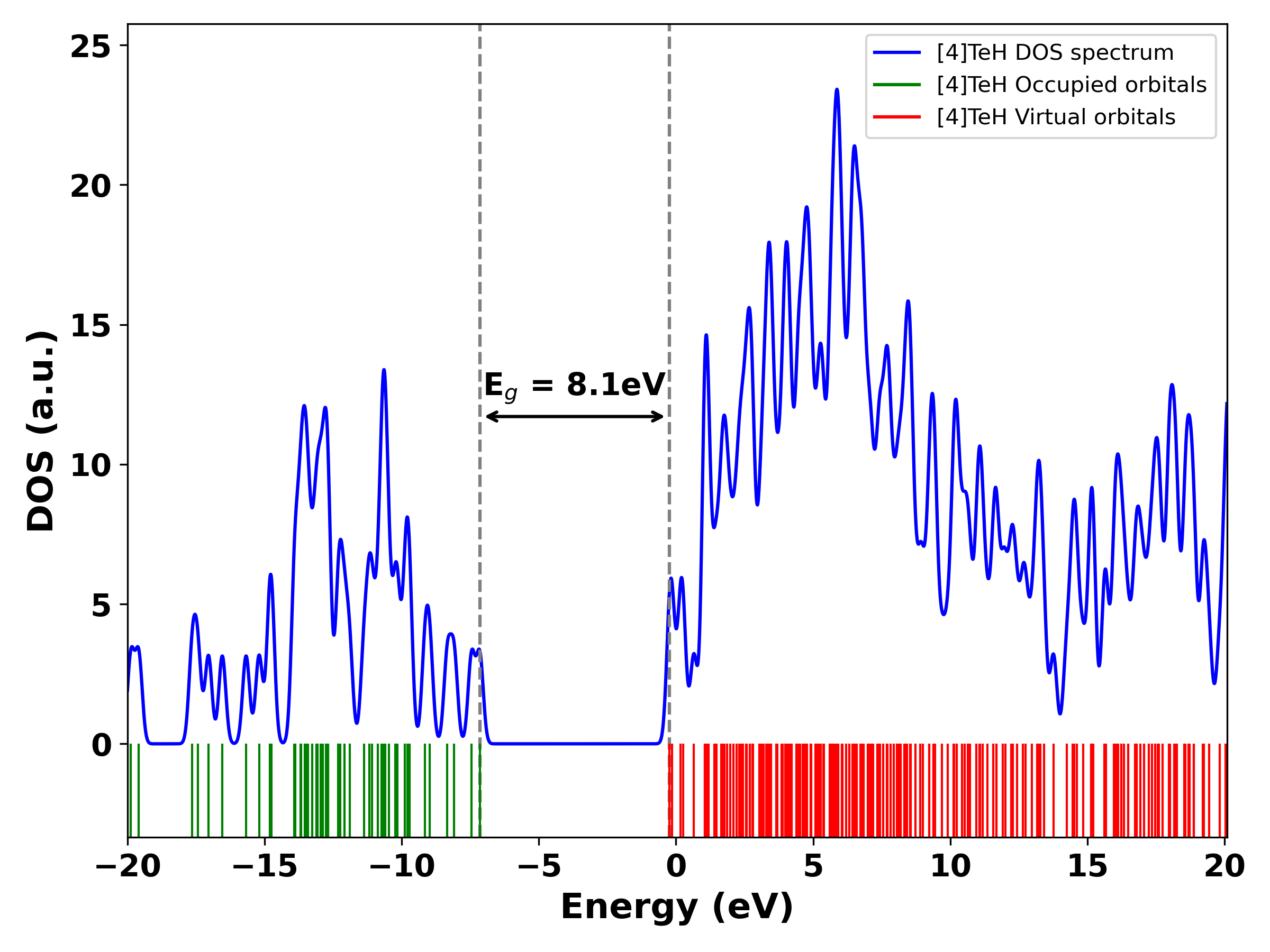


**S5c ([3]TeH) S5d ([4]TeH)**


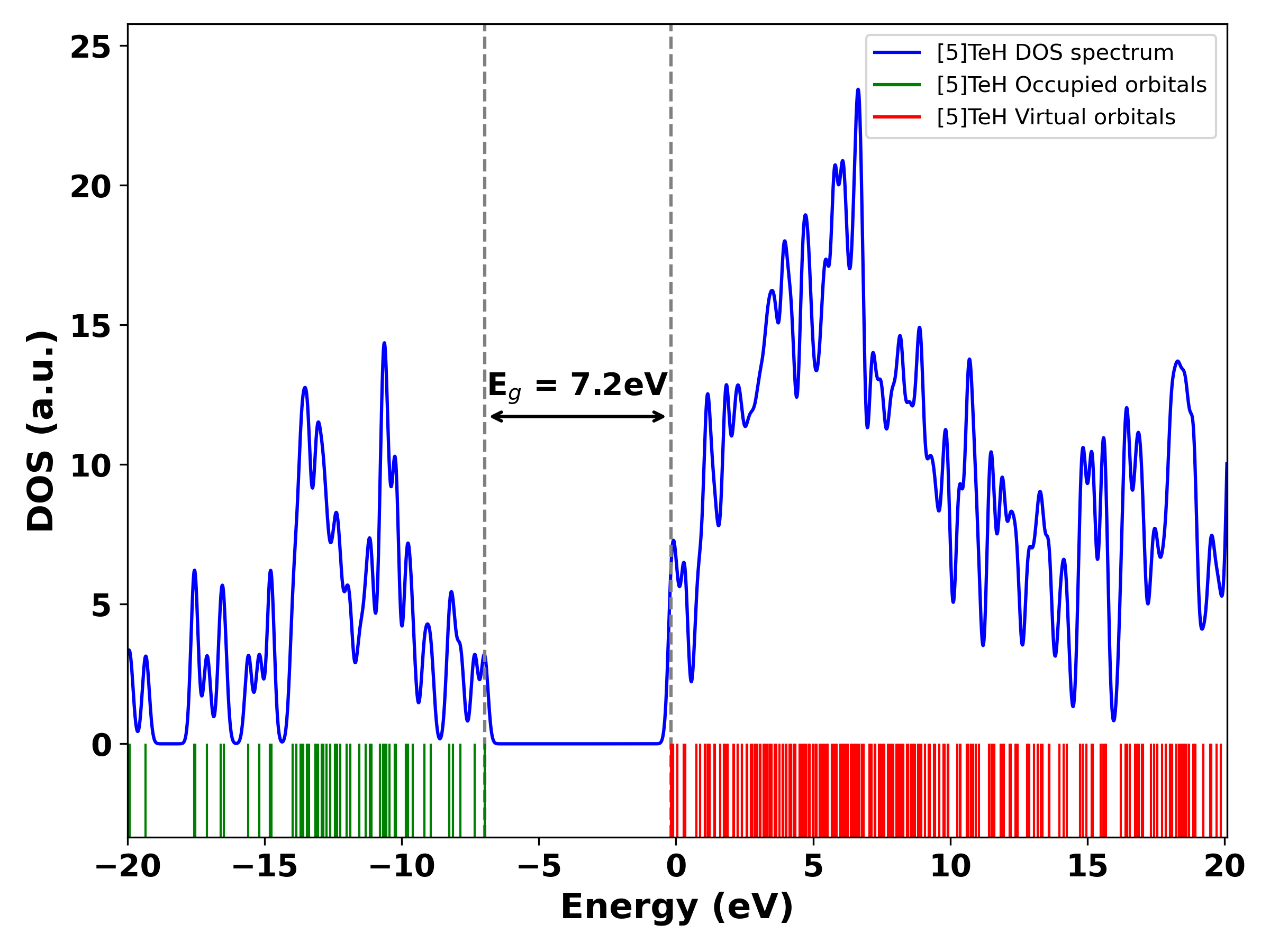

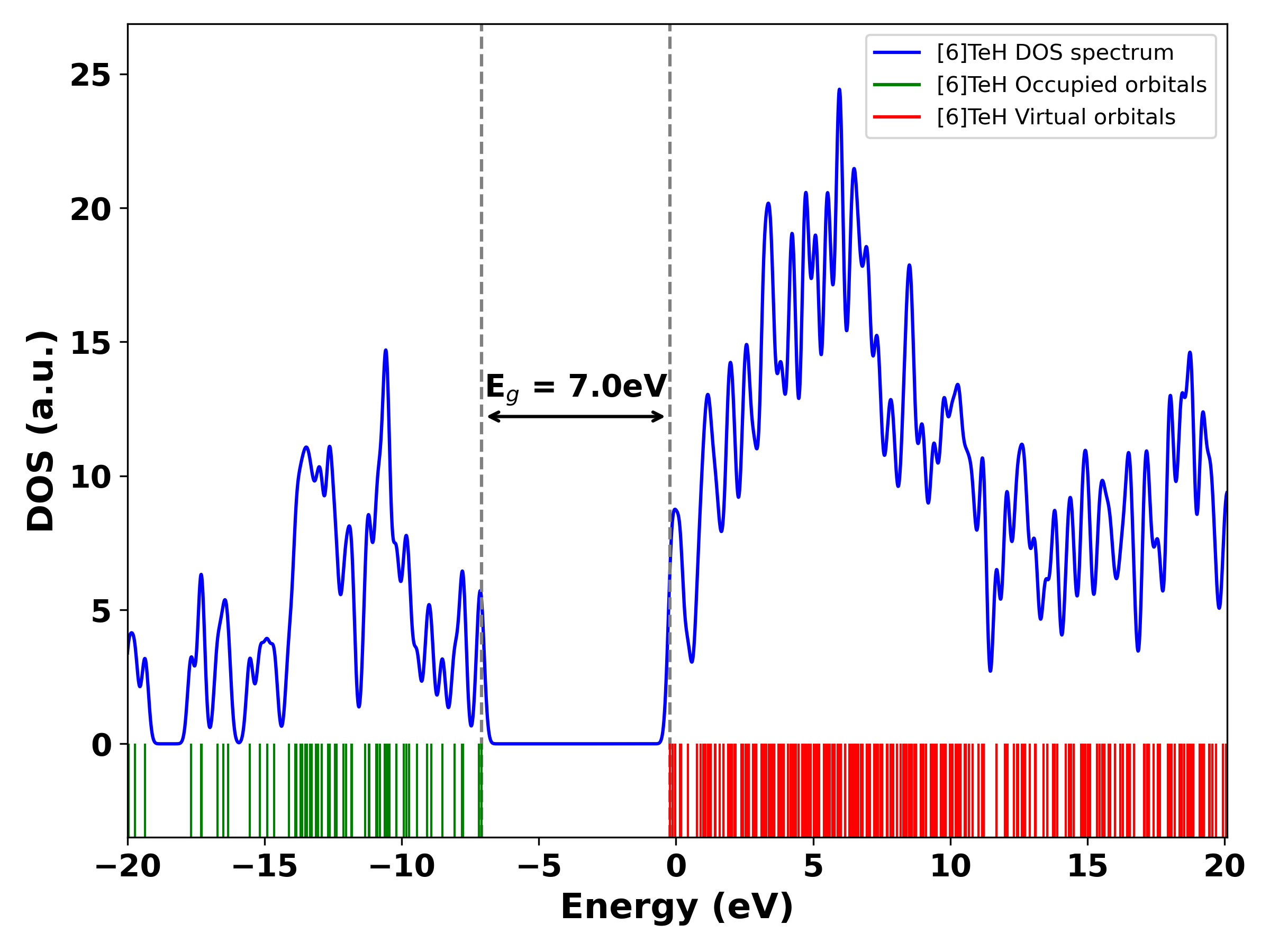


**S5e ([5]TeH) S5f ([6]TeH)**


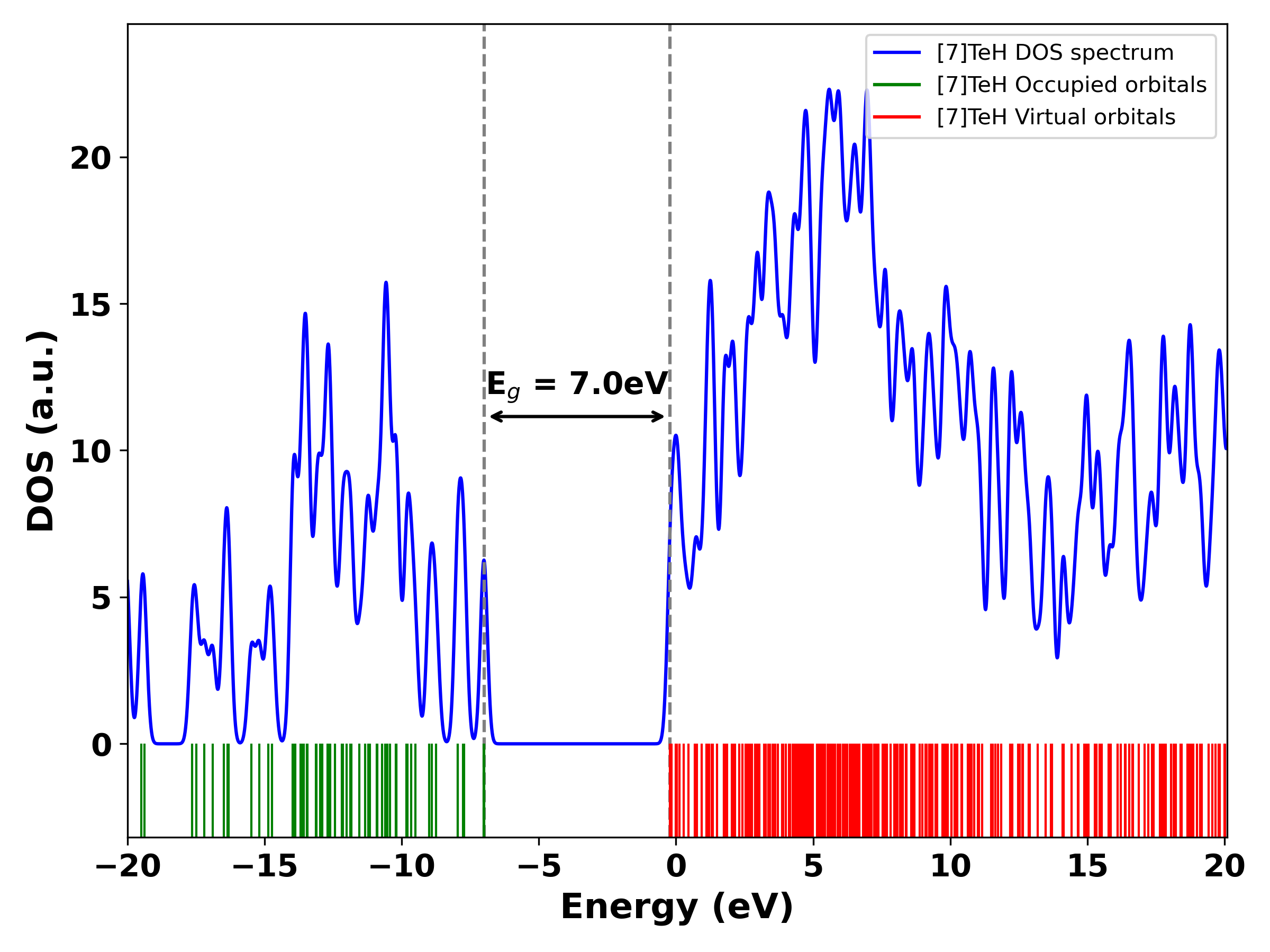

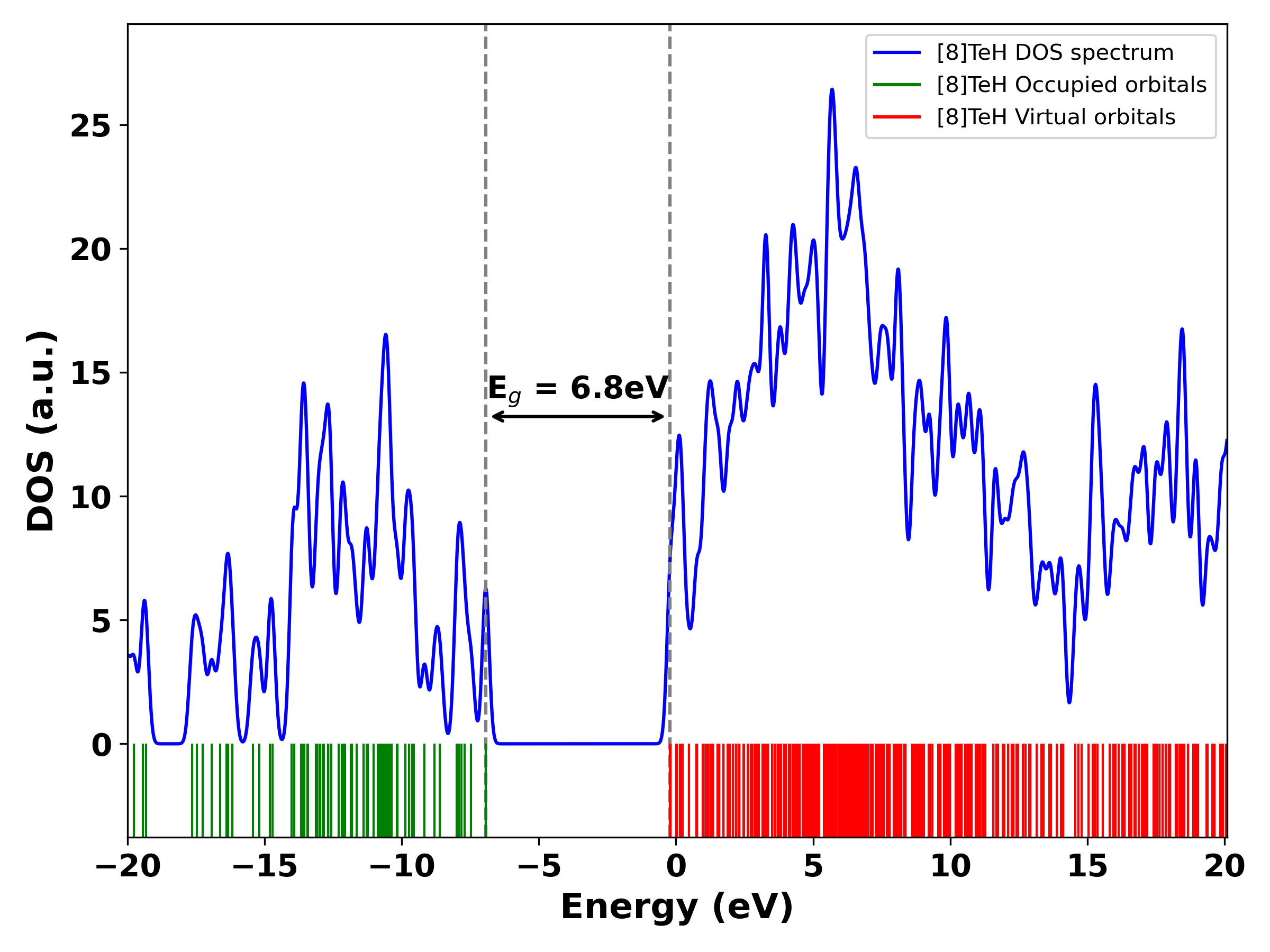


**S5g ([7]TeH) S5h ([8]TeH)**


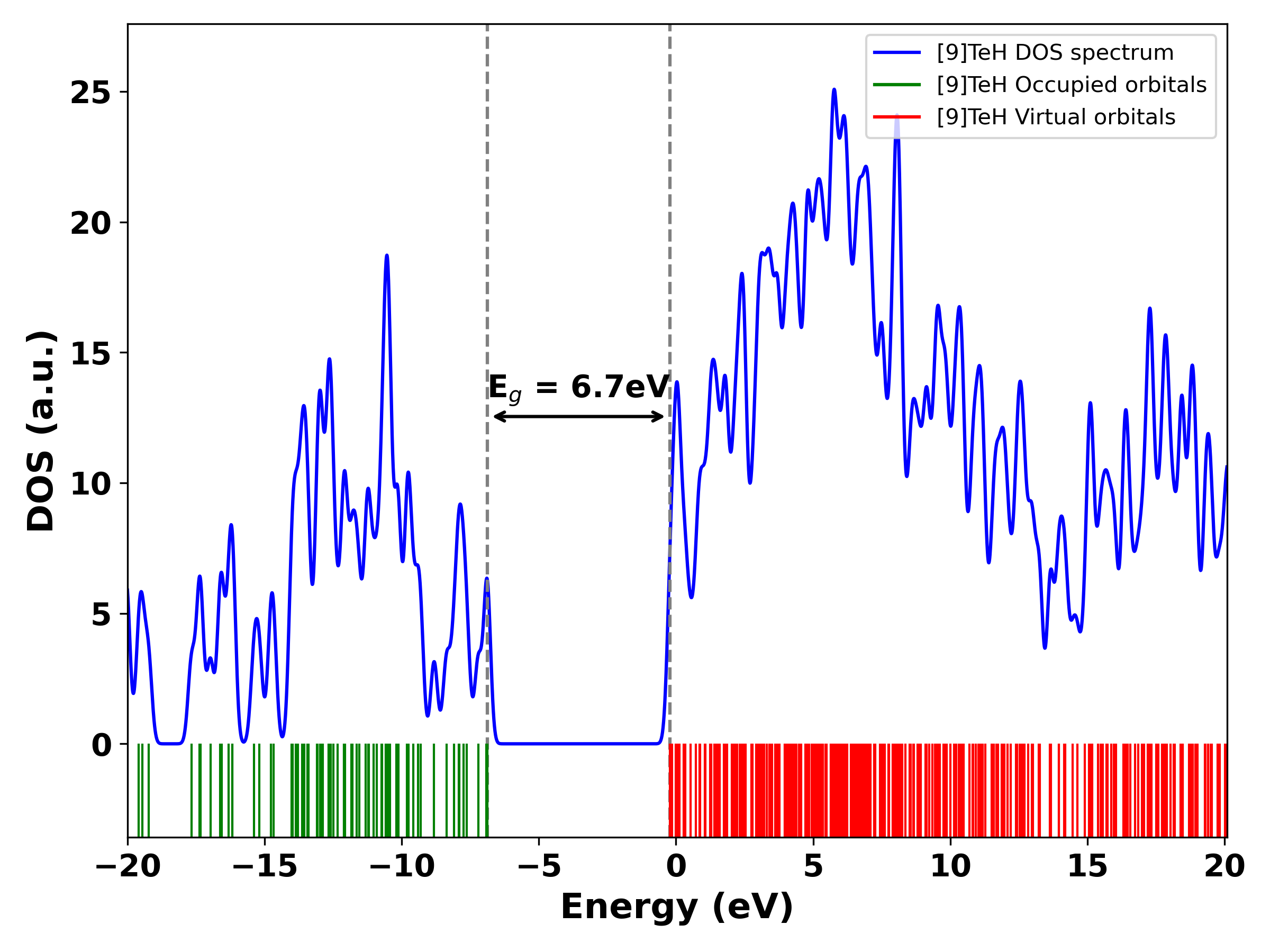

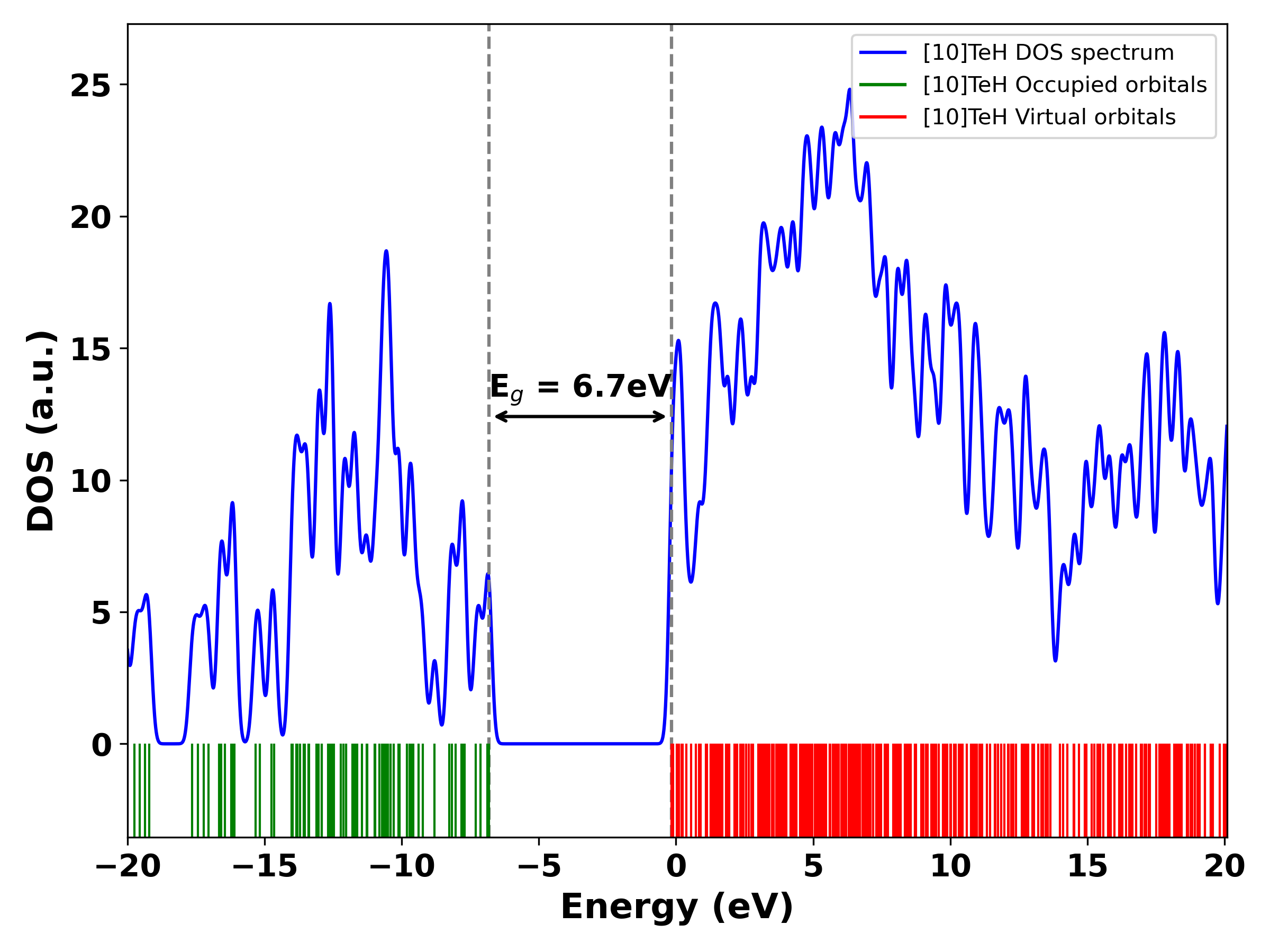


**S5i ([9]TeH) S5j ([10]TeH)**

**Figure S5. T**elluro[n]helicenes, [n]TeH (n=1-10, a-j) calculated using single point energy calculations using ωB97XD functional with geometries obtained at B3LYP functional. Basis set used: 6-311++G(d,p) for H, C, Si, S, Se, Br atoms and def2-TZVPP basis set for Te atoms.

**11. HOMO-LUMO gaps (E_g_), for thia[n]helicenes, [n]TH, and seleno[n]helicenes, [n]SH and telluro[n]helicenes, [n]TeH**

**Table S13**. Calculated HOMO-LUMO gaps (E_g_), for thia[n]helicenes, [n]TH, n=1-5 and seleno[n]helicenes, [n]SH, n=1-5 and telluro[n]helicenes, [n]TeH, n=1-5 in gas phase.

| **Number of rings (n)** | **E_g_ for [n]TH (in eV)^a^** | **E_g_ for [n]SH (in eV) ^a^** | **E_g_ for [n]TeH (in eV) ^a^** | **E_g_ for [n]TeH (in eV)^b^** |
| --- | --- | --- | --- | --- |
| **1** | 7.62 | 7.4 | 6.82 | 8.27 |
| **2** | 7.33 | 7.09 | 6.05 | 7.55 |
| **3** | 7.1 | 6.61 | 5.66 | 7.52 |
| **4** | 6.94 | 6.48 | 5.48 | 7.30 |
| **5** | 6.75 | 6.34 | 5.42 | 7.10 |

**Method:** B2PLYP^a^ functional including Grimme’s D3 dispersion correction with Becke-Johnson damping is used. E_g_ for telluro[n]helicenes calculated using ωB97XD^b^ functional. Basis set used: 6-311++G(d,p) for H, C, Si, S, Se, Br atoms and def2-TZVPP basis set for Te atoms.

**Table S14**. Calculated HOMO-LUMO gaps (E_g_), for telluro[n]helicene, [1]TeH in gas phase.^1^ with varying ω in bohr^-1^

| **Tuning parameter (ω in bohr^-1^)** | **E_g_ for [1]TH (in eV) with**  **LANL2DZ basis set for Te atoms** | **E_g_ for [1]TH (in eV)**  **def2-TZVP basis set for Te atoms** |
| --- | --- | --- |
| **0.1** | 4.94 | 4.99 |
| **0.2** | 6.76 | 6.74 |
| **0.3** | 7.67 | 7.56 |
| **0.4 (default value)** | 9.71 | 9.29 |
| **0.5** | 8.34 | 8.70 |

**Method:** LC-ωPBE DFT functional. Basis set used: LANL2DZ and def2-TZVP basis sets for Te atom and 6-311++G(d,p) for all other atoms.


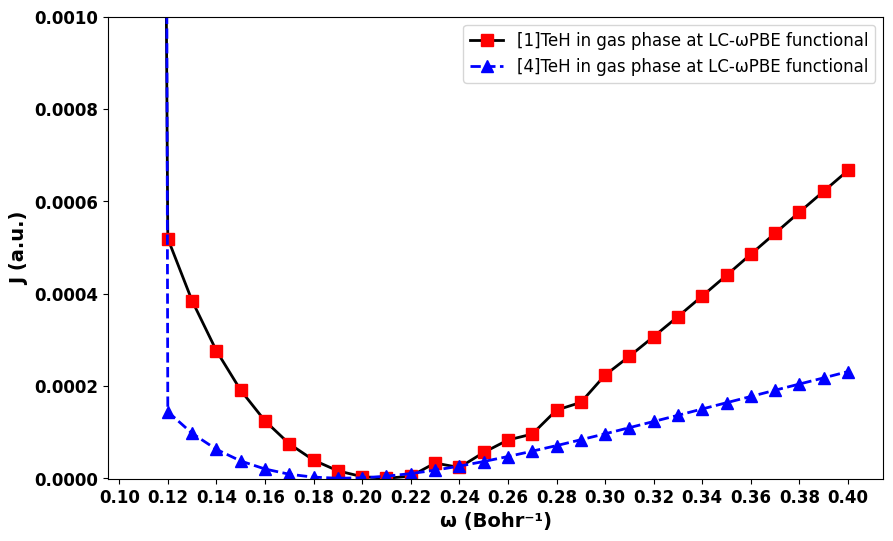

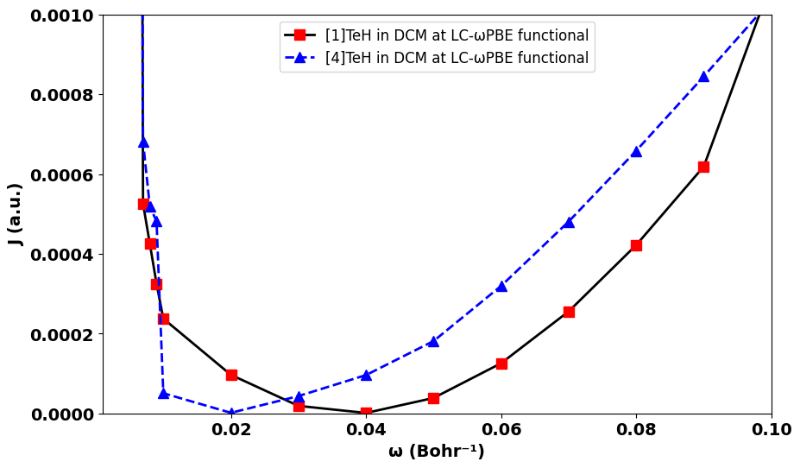


1. **b)**


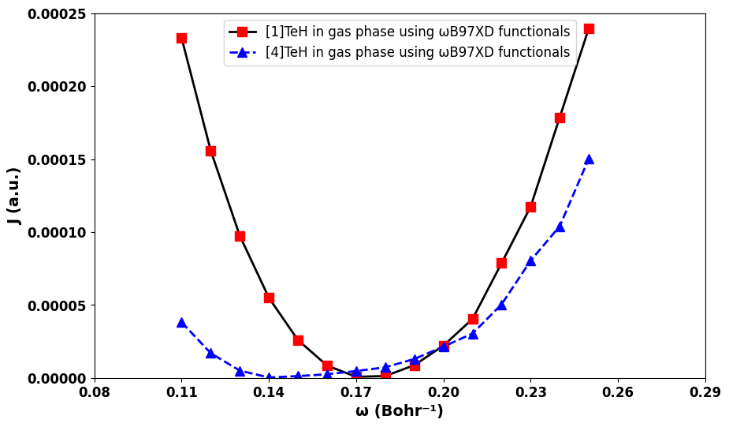

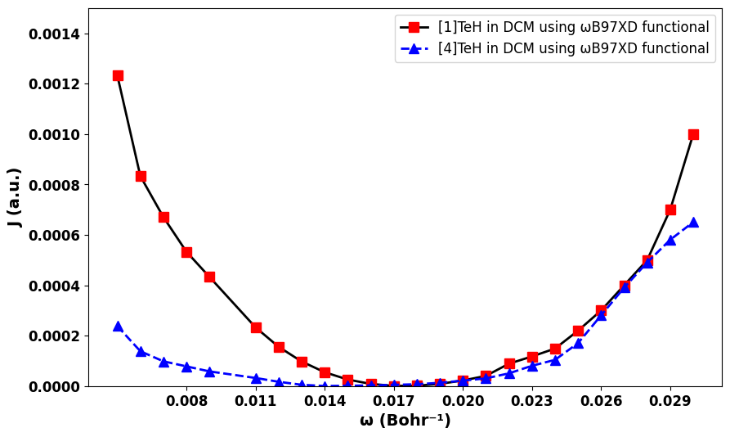


**c) d)**

**Figure S6.** Optimized range-separation parameter (ω) values satisfying Koopmans' theorem for (a) telluro[1]helicene (0.21 bohr⁻¹) and telluro[4]helicene (0.19 bohr⁻¹) in gas phase at LC-ωPBE functional, (b) telluro[1]helicene (0.04 bohr⁻¹) and telluro[4]helicene (0.02 bohr⁻¹) in DCM solvent at LC-ωPBE functional, (c) telluro[1]helicene (0.17 bohr⁻¹) and telluro[4]helicene (0.14 bohr⁻¹) in gas phase at ωB97XD functional, (d) telluro[1]helicene (0.018 bohr⁻¹) and telluro[4]helicene (0.012 bohr⁻¹) in DCM solvent. The deviation from Koopmans’ condition, 𝐽=(𝜀_HOMO_ + IP)^2^, was minimized to determine the optimal ω values. Basis set used: LANL2DZ basis sets for Te atoms and 6-311++G(d,p) for all other atoms. Solvation model: SMD

**12. Computational cost comparison**

**Table S15:** Computational cost for thia[n]helicenes, [n]TH, seleno[n] helicenes, [n]SH, Telleuro[n]helicenes, [n]TeH, n=1-10 HOMO-LUMO gap values in gas phase.

|  | **Thia[n]helicenes, [n]TH, n=1-10** | | |
| --- | --- | --- | --- |
|  | *ωB97XD Functional Full optimization* | *B3LYP Functional Full optimization* | *ωB97XD Functional single point energy calculation* |
|  | *CPU time* | *CPU time* | *CPU time* |
| *1* | *12 minutes 47.1 seconds* | *1 minutes 2.3 seconds* | *1 minutes 49.9 seconds* |
| *2* | *18 minutes 35.7 seconds* | *1 minutes 26.3 seconds* | *2 minutes 9.7 seconds* |
| *3* | *22 minutes 21.2 seconds* | *1 minutes 54.5 seconds* | *3 minutes 19.3 seconds* |
| *4* | *41 minutes 8.6 seconds* | *2 minutes 47.4 seconds* | *4 minutes 15.5 seconds* |
| *5* | *1 hours 55 minutes 40.8 seconds* | *3 minutes 38.7 seconds* | *7 minutes 6.4 seconds* |
| *6* | *2 hours 6 minutes 24.9 seconds* | *15 minutes 40.2 seconds* | *8 minutes 24.3 seconds* |
| *7* | *2 hours 28 minutes 23.9 seconds* | *22 minutes 59.6 seconds* | *12 minutes 21.3 seconds* |
| *8* | *2 hours 9 minutes 20.5 seconds* | *20 minutes 40.2 seconds* | *17 minutes 9.2 seconds* |
| *9* | *5 hours 23 minutes 7.9 seconds* | *29 minutes 49.1 seconds* | *20 minutes 58.1 seconds* |
| *10* | *3 hours 59 minutes 5.3 seconds* | *48 minutes 44.7 seconds* | *27 minutes 44.7 seconds* |
| *Total* | *19 hours, 36 minutes, and 55.9 seconds* | *2 hours, 28 minutes and 43 seconds* | *1 hours, 45 minutes and 43 seconds* |
|  | **Seleno[n]helicenes, [n]SH, n=1-10** | | |
| *1* | *0 hours 12 minutes 39.4 seconds* | *7 minutes 7.9 seconds* | *0 minutes 53.6 seconds* |
| *2* | *19 minutes 39.1 seconds* | *10 minutes 42.8 seconds* | *0 hours 1 minutes 29.6 seconds* |
| *3* | *36 minutes 23.5 seconds* | *19 minutes 46.5 seconds* | *2 minutes 6.0 seconds* |
| *4* | *51 minutes 47.8 seconds* | *32 minutes 13.2 seconds* | *1 minutes 50.8 seconds* |
| *5* | *1 hours 23 minutes 1.5 seconds* | *58 minutes 15.3 seconds* | *10 minutes 41.6 seconds* |
| *6* | *2 hours 43 minutes 26.3 seconds* | *1 hours 17 minutes 56.4 seconds* | *6 minutes 19.9 seconds* |
| *7* | *5 hours 14 minutes 24.3 seconds* | *2 hours 32 minutes 35.1 seconds* | *0 hours 9 minutes 1.5 seconds* |
| *8* | *5 hours 6 minutes 55.1 seconds* | *2 hours 59 minutes 25.4 seconds* | *0 hours 11 minutes 14.7 seconds* |
| *9* | *8 hours 57 minutes 49.4 seconds* | *4 hours 4 minutes 15.7 seconds* | *15 minutes 53.8 seconds* |
| *10* | *5 hours 12 minutes 45.2 seconds* | *3 hours 46 minutes 58.8 seconds* | *13 minutes 11.2 seconds* |
| *Total* | *30 hours, 38 minutes, and 51.6 seconds.* | *19 hours, 18 minutes, and 0.1 seconds* | *2 hours, 58 minutes, and 25.7 seconds.* |
|  | **Telluro[n]helicenes, [n]TeH, n=1-10** | | |
| *1* | *0 hours 12 minutes 39.4 seconds* | *9 minutes 20.8 seconds* | *2 minutes , 12.8 seconds* |
| *2* | *23 minutes 39.1 seconds* | *1 hours 44 minutes 48.9 seconds* | *3 minutes 16.5 seconds* |
| *3* | *38 minutes 33.5 seconds* | *20 minutes 29.7 seconds* | *5 minutes, 18.0 seconds* |
| *4* | *59 minutes 57.8 seconds* | *39 minutes 34.6 seconds* | *7 minutes , 24.5 seconds* |
| *5* | *1 hours 53 minutes 20.5 seconds* | *1 hours 14 minutes 0.4 seconds* | *9 minutes , 30.0 seconds* |
| *6* | *1 hours 53 minutes 26.3 seconds* | *6 hours 58 minutes 31.6 seconds* | *13 minutes, 41.0 seconds* |
| *7* | *6 hours 14 minutes 24.3 seconds* | *1 hours 49 minutes 3.3 seconds* | *13 minutes , 46.9 seconds* |
| *8* | *7 hours 6 minutes 55.1 seconds* | *1 hours 44 minutes 28.5 seconds* | *16 minutes, 54.6 seconds* |
| *9* | *9 hours 57 minutes 29.4 seconds* | *3 hours 58 minutes 51.7 seconds* | *18 minutes 4.7 seconds* |
| *10* | *7 hours 12 minutes 55.2 seconds* | *1 hours 53 minutes 3.8 seconds* | *27 minutes 3.5 seconds* |
| *Total* | *36 hours, 33 minutes, and 20.6 seconds.* | *20 hours, 32 minutes, and 13 seconds* | *1 hour, 36 minutes, and 32.5 seconds.* |

**^#^**SMP Processors: Each worker uses 36 SMP processors, enabling shared-memory parallelism within each node.

Linda Workers: Linda enables distributed-memory parallelism across nodes, combining shared-memory parallelism within nodes.

Comparison

***ωB97XD Functional full optimization*: 86 hours, 49 minutes, and 8.1 seconds.**

**B3LYP Functional full optimization and ωB97XD Functional single point energy calculation: 48 hours, 39 minutes, and 37.3 seconds**

Basis set used: LANL2DZ basis set for Te atom and 6-311++G(d,p) for all other atoms.

**13. Simulated electronic absorptions spectra [3]TH and [7]TH in DCM, and experimentally reported UV-Visible absorption spectra of 1 [7]TH and 4 [3]TH in DCM**

**S7a S7b**


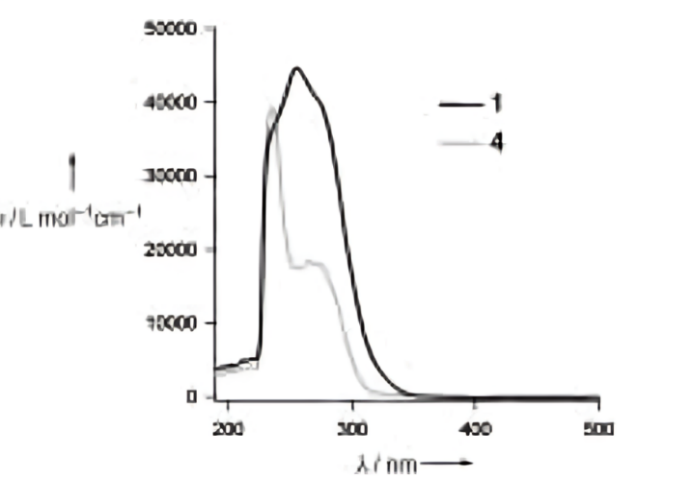


**S7c**

**Figure S7**. a) Simulated electronic absorptions spectra [3]TH in DCM, b) Simulated electronic absorptions spectra [3]TH in DCM, and c) experimentally reported UV-Visible absorption spectra of 1 [7]TH and 4 [3]TH in DCM^2^

|  | **Absorption maxima values (λ_max_ in nm)** | | | |
| --- | --- | --- | --- | --- |
| **Systems** | **LC-ωPBE** | **ω-B97XD** | **CAM-B3LYP** | **Experimental values** |
| [3]TH | 204, 230 | 214, 242 | 223, 246 | 236, 266 |
| [7]TH | 227, 237 | 239, 248 | 230, 255 | 235, 256 |

**Methods:** CAM-B3LYP, ω-B97XD, and LC-ωPBE functionals are used to calculate the UV-Vis spectra for [3]TH and [7]TH applying TDDFT procedure.

**14.** **TDDFT calculations of neutral tellurophene-based [n]helicene (n=1-10) in DCM solvent**

**Table S16**. Selected absorption bands (λ_max_, in nm) with corresponding oscillator strength (*f*) extracted from UV-Vis spectral data (50 lowest electronic transitions) of tellurophene-based [n]helicenes, [n]TeH (n=1-5) and corresponding radical cations, [n]TeH^•+^ in DCM solvent.

| **n** | **λ_max_ (nm) and *f*** | **Electronic Transitions^a^ in UV region** | **n** | **λ_max_ (nm) and *f*** | **Electronic Transitions^a^ in UV region** |
| --- | --- | --- | --- | --- | --- |
| 1 | 288, (0.129) | H → L (0.68) | 6 | 322, (0.189) | H-1 → L (-0.23), H-1 → L+2 (0.39) |
|  | 260, (0.170) | H → L+2 (0.68) |  | 296, (0.128) | H-2 → L+2 (-0.22), H → L+8 (0.24) |
| 2 | 313, (0.190) | H → L+1 (0.67) | 7 | 332, (0.112) | H-1 → L+1 (0.38), H → L+2 (-0.35) |
|  | 276, (0.041) | H-1 → L+1 (0.67) |  | 321, (0.097) | H-1 → L+7 (0.42), H-1 → L+8 (0.29) |
|  | 240, (0.580) | H-1 → L+3 (0.63) |  | 288, (0.186) | H-4 → L+3 (0.29), H-1 → L+7 (-0.24) |
| 3 | 316, (0.133) | H-1 → L (0.45), H → L+1 (0.41) | 8 | 325, (0.109) | H →L+3 (0.24), H → L+6 (0.42) |
|  | 306, (0.078) | H → L+2 (0.51) |  | 284, (0.282) | H-3 →L+2 (0.21), H → L+11 (0.23) |
|  | 292, (0.234) | H → L+3 (0.51) |  | 273, (0.251) | H-4 →L (-0.23), H-4 →L+2 (0.25) |
|  | 272, (0.132) | H-1 → L+3 (0.44) |  | 266, (0.057) | H-3 →L+2 (-0.21), H →L+11 (0.21) |
| 4 | 322, (0.121) | H-1 → L+1 (0.43), H → L (-0.39) | 9 | 341, (0.022) | H-1 → L+2 (0.38), H → L (0.38) |
|  | 301, (0.132) | H → L+3 (0.29), H → L+2 (-0.38) |  | 332, (0.109) | H-2 → L+1 (-0.31), H → L+1 (0.38) |
|  | 261, (0.155) | H-2 → L+2 (0.42), H → L+8 (0.28) |  | 282, (0.358) | H-6 → L+3 (0.24), H-4 → L+4 (0.20) |
| 5 | 354, (0.068) | H → L+2 (0.63) | 10 | 340, (0.015) | H-1 → L+3 (0.24), H → L+2 (0.38) |
|  | 320, (0.202) | H-1 → L (0.32), H-1 → L+12 (0.33) |  | 330, (0.088) | H-1 → L+3 (0.34), H-1 → L+7 (-0.27) |
|  | 296, (0.207) | H-2 → L+1 (-0.25), H → L+6 (0.34) |  | 282, (0.233) | H-3 → L+4 (0.27), H-4 → L+2 (0.20) |

TD-CAM-B3LYP functional with 6-311++G(d,p) basis set for H, C, Br, Si, atoms and lanl2dz basis set is considered for Te atoms.

**^a^**H = Highest occupied molecular orbital (HOMO) & L = Lowest unoccupied molecular orbital (LUMO).

Values in the braces show the relative contribution of the transition for the optical band. Transitions with major contributions are only shown.

**Table S17**. Selected absorption bands (λ_max_, in nm) with corresponding oscillator strength (*f*) extracted from UV-Vis spectral data (50 lowest electronic transitions) of tellurophene-based [n]helicenes radical cations, [n]TeH^•+^ in DCM solvent.

| **n** | **λ_max_ (nm) and *f*** | **Electronic Transitions^a^ in**  **UV region** | **n** | **λ_max_ (nm) and *f)*** | **Electronic Transitions^b^ in visible and IR regions** |
| --- | --- | --- | --- | --- | --- |
| 1 | 1174, (0.001) | H → L (0.95) | 6 | 1186, (0.188) | H-1 → L (0.89) |
|  | 445, (0.051) | H-2 → L (0.88) |  | 887, (0.074) | H-2 → L (0.88) |
| 2 | 1614, (0.029) | H → L (0.94) | 7 | 2104, (0.088) | H-1 → L (0.99) |
|  | 1045, (0.102) | H-1 → L (0.92) |  | 1488, (0.132) | H-2 → L (0.92) |
|  | 500, (0.070) | H-3 → L (0.93) |  | 1068, (0.089) | H-3→ L (0.92) |
| 3 | 2085, (0.011) | H-1 → L (0.89) | 8 | 1384, (0.142) | H-2 → L (0.98) |
|  | 1047, (0.130) | H-2 → L (0.88) |  | 1135, (0.207) | H-1 → L (0.65) |
|  | 793, (0.029) | H-3 → L (0.96) |  | 806, (0.042) | H-5 → L (0.90) |
|  | 593, (0.068) | H-1 → L (0.89) |  | 571, (0.039) | H-6 → L (0.97) |
| 4 | 1127, (0.196) | H-1 → L (0.93) | 9 | 1557, (0.053) | H-2 → L (0.73) |
|  | 677, (0.052) | H-3 → L (0.92) |  | 1413, (0.149) | H-3 → L (0.96) |
|  | 575, (0.075) | H-4 → L (0.91) |  | 972, (0.010) | H-2 → L (0.58) |
| 5 | 1185, (0.240) | H-1 → L (0.96) | 10 | 1683, (0.044) | H-3 → L (0.80) |
|  | 756, (0.054) | H-2 → L (0.74) |  | 1574, (0.107) | H-2 → L (0.74) |
|  | 539, (0.035) | H-4 → L (0.80) |  | 809, (0.086) | H-6 → L (0.80) |

TD-CAM-B3LYP functional with 6-311++G(d,p) basis set for H, C, Br, Si, atoms and lanl2dz basis set is considered for Te atoms. The SMD model is used to incorporate effect of DCM solvent.

**^a^**H = Highest doubly occupied molecular orbital (HDOMO) & L = Lowest singly occupied molecular orbital (LSOMO).

Values in the braces show the relative contribution of the transition for the optical band.

Transitions with major contributions are only shown.

**15. UV-Vis spectra for telluro[n]helicenes (n=1-10) and their corresponding radical cations**

**S8a S8b**

**S8c S8d**

**Figure S8**. Simulated electronic absorptions spectra, a) [n]TeH, n=1-5,b) [n]TeH, n=6-10 c) comparison of electronic absorptions spectra [5]TeH and d) [7]TeH in DCM solvent using CAM-B3LYP and ω-B97XD functionals. Basis set used: LANL2DZ basis set for Te atoms and 6-311++G(d,p) basis set for all other atoms. Solvent effect in DCM is accounted by using SMD model.

**S9a S9b**

**9c**

**Figure S9**. Simulated electronic absorptions spectra, a) [n]TeH^•+^, n=1-5 and b) [n]TeH^•+^, n=6-10 and c) comparison of electronic absorptions spectra [7]TeH^•+^calculated in DCM solvent using CAM-B3LYP and ω-B97XD Functionals. Basis set used: LANL2DZ basis set for Te atoms and 6-311++G(d,p) basis set for all other atoms. Solvent effect in DCM is accounted by using SMD model.

**16** **Molecular orbital plots with contour cutoff = 0.025 a.u. for the most stable structure of end substituted telluro[n]helicenes radical cation calculated at B3LYP-D functional with 6-311++G(d,p) basis set for H, C, Si, and Br atoms and lanl2dz basis set for Te atoms in DCM solvent.**

1. **[1]TeH^●+^**

**
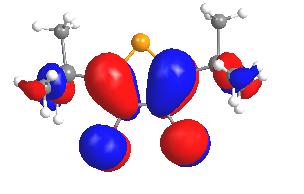

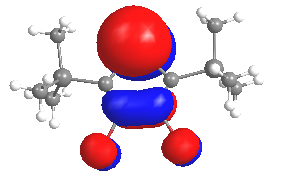
**

**S10a1(H) S10a2(L)**

**
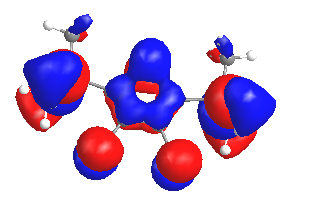
**

**S10a3(H-2)**

1. **[2]TeH^●+^**

**
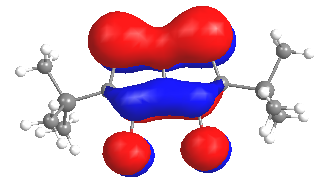

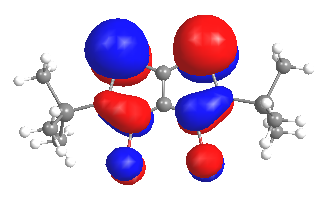
**

**S10b1 (H-1) S10b2 (L)**

**
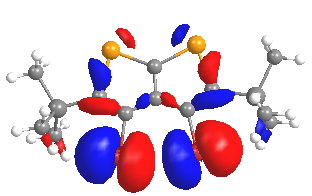
**

**S9b3 (H-2)**

1. **[3]TeH^●+^**

**
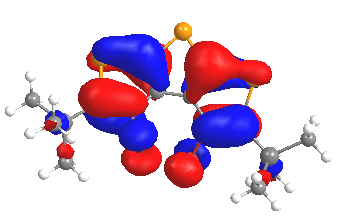

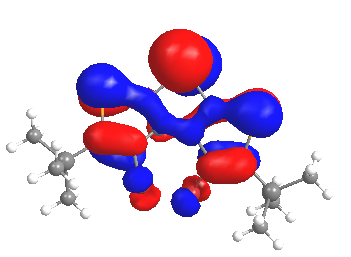
**

**S10c1 (H-1) S10c2 (L)**

**
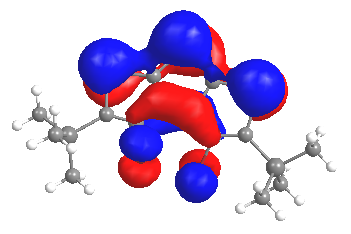

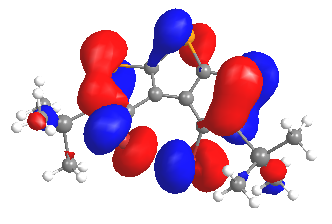
**

**S10c3(H-2) S10c4(H-3)**

1. **[4]TeH^●+^**

**
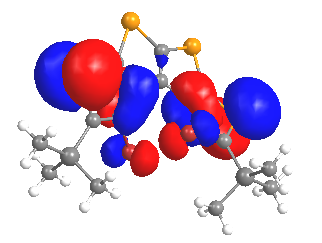

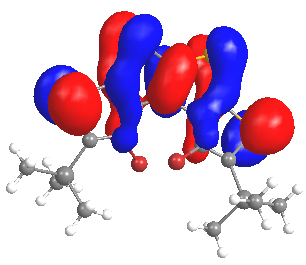
**

**S10d1(H-1) S10d2(L)**

**
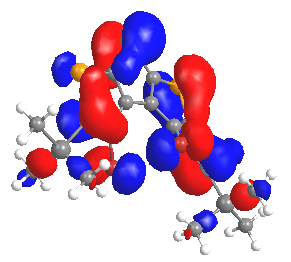

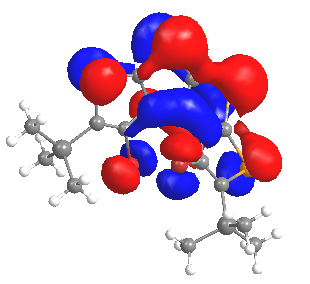
**

**S10d3(H-3) S10d4(H-4)**

1. **[5]TeH^●+^**

**
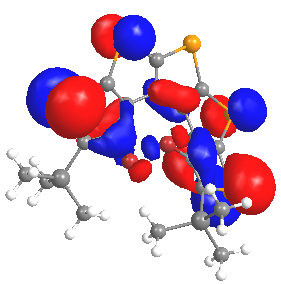

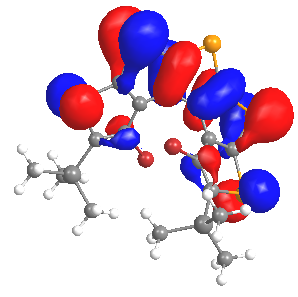
**

**S10e1(H-1) S10e2(L)**

**
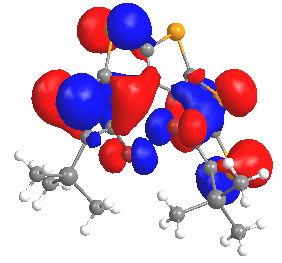

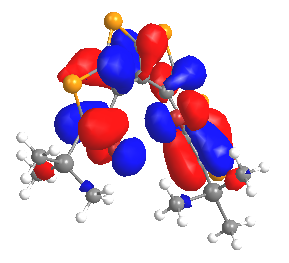
**

**S10e3(H-2) S10e4(H-3)**

1. **[6]TeH^●+^**

**
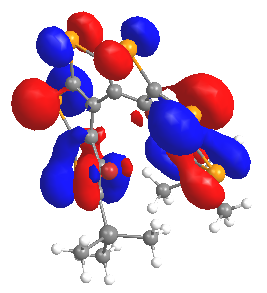

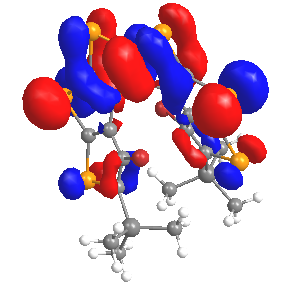
**

**S10f1(H-1) S10f2(L)**

**
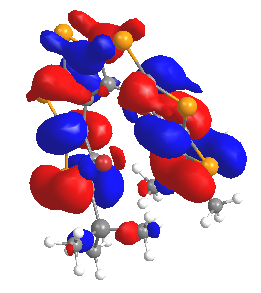

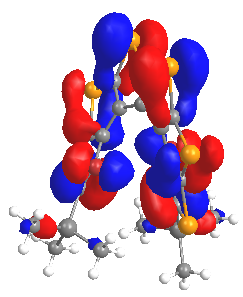
**

**S10f3(H-2) S10f4(H-5)**

1. **[7]TeH^●+^**

**
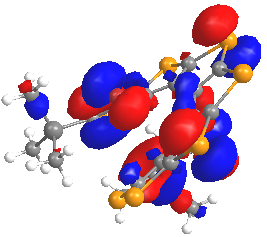
**

**S10g1(H-5)**

1. **[8]TeH^●+^**

**
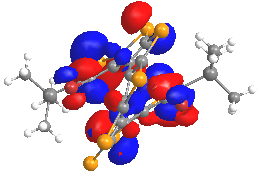

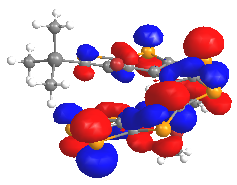
**

**S10h1(H-1) S10h2(L)**

**
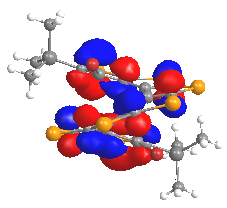

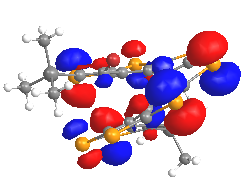
**

**S10h3(H-2) S10h4(H-5)**

1. **[9]TeH^●+^**

**
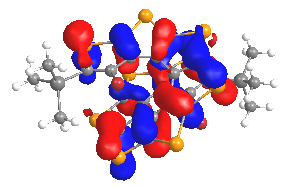

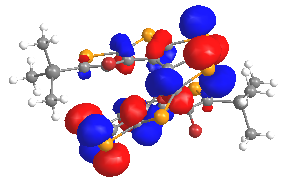
**

**S10i1(H-2) S10i2(L)**

**
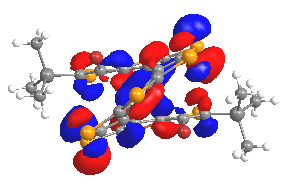

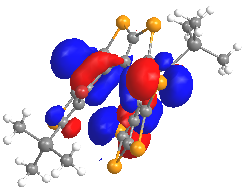
**

**S10i2(H-3) S10i1(H-4)**

1. **[10]TeH^●+^**

**
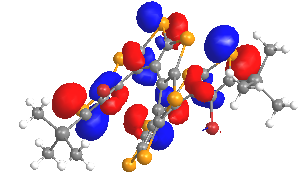

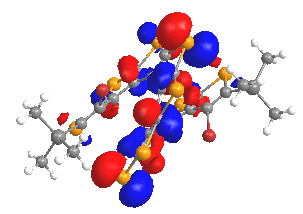
**

**S10j1(H-2) S10j2(L)**

**
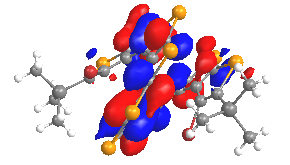

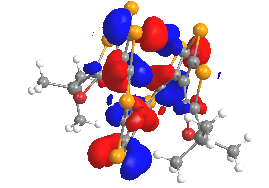
**

**S10j1(H-3) S10j1(H-5)**

**Figure S10** Molecular orbital plot for telluro[n]helicenes radical cation, [n]TeH^•+^, n=1-10, S7a-S7j calculated using B3LYP-D as functional with 6-311++G(d,p) basis set for H, C, Si, and Br atoms and lanl2dz basis set for Te atoms in DCM solvent with contour cutoff= 0.03 a.u.

**References:**

(1) Vikramaditya, T.; Chai, J. Da; Lin, S. T. Impact of Non-Empirically Tuning the Range-Separation Parameter of Long-Range Corrected Hybrid Functionals on Ionization Potentials, Electron Affinities, and Fundamental Gaps. *J. Comput. Chem.* **2018**, *39* (28), 2378–2384. https://doi.org/10.1002/jcc.25575.

(2) Rajca, A.; Wang, H.; Pink, M.; Rajca, S. Annelated Heptathiophene: A Fragment of a Carbon–Sulfur Helix. *Angew. Chem. Int. Ed.* **2000**, *39* (24), 4655–4657.
